# The Acheulian and Early Middle Paleolithic in Latium (Italy): Stability and Innovation

**DOI:** 10.1371/journal.pone.0160516

**Published:** 2016-08-15

**Authors:** Paola Villa, Sylvain Soriano, Rainer Grün, Fabrizio Marra, Sebastien Nomade, Alison Pereira, Giovanni Boschian, Luca Pollarolo, Fang Fang, Jean-Jacques Bahain

**Affiliations:** 1 University of Colorado Museum, Boulder, Colorado, 80309–0265, United States of America; 2 Istituto Italiano di Paleontologia Umana, Rome, Italy; 3 School of Geography, Archaeology and Environmental Studies, University of the Witwatersrand, Johannesburg, South Africa; 4 ArScAn, AnTET, CNRS, Université Paris Ouest, 92023, Nanterre, France; 5 Research School of Earth Sciences, The Australian National University, Canberra, ACT, 2601, Australia; 6 Research Centre of Human Evolution, Griffith University, Nathan, QLD, 4111, Australia; 7 Istituto Nazionale di Geofisica e Vulcanologia, 00147, Roma, Italy; 8 Laboratoire des Sciences du climat et de l’environnement, CEA Saclay, UMR 8212, UVSQ et Université de Paris-Saclay, 91198, Gif-sur-Yvette, France; 9 Département de Préhistoire, Museum National d’Histoire Naturelle, UMR 7194, 75013, Paris, France; 10 Sezione di Scienze Preistoriche e Antropologiche, Dipartimento di Studi Umanistici, Università di Ferrara, Ferrara, Italy; 11 Ecole française, Roma, Italy; 12 Dipartimento di Biologia, Università di Pisa, 56126, Pisa, Italy; 13 Laboratoire Archéologie et Peuplement de l’Afrique, Department of Genetics and Evolution, University of Geneva, 1211, Geneva, Switzerland; Universidade do Algarve, PORTUGAL

## Abstract

We present here the results of a technological and typological analysis of the Acheulian and early Middle Paleolithic assemblages from Torre in Pietra (Latium, Italy) together with comparisons with the Acheulian small tools of Castel di Guido. The assemblages were never chronometrically dated before. We have now ^40^Ar/^39^Ar dates and ESR-U-series dates, within a geomorphological framework, which support correlations to marine isotope stages. The Acheulian (previously correlated to MIS 9) is now dated to MIS 10 while the Middle Paleolithic is dated to MIS 7. Lithic analyses are preceded by taphonomic evaluations. The Levallois method of the Middle Paleolithic assemblage is an innovation characterized by the production of thin flake blanks without cortex. In contrast, the small tool blanks of the Acheulian were either pebbles or thick flakes with some cortex. They provided a relatively easy manual prehension. The choice of Levallois thin flake blanks in the Middle Paleolithic assemblage suggest that the new technology is most likely related to the emergence of hafting. Accordingly, the oldest direct evidence of hafting technology is from the site of Campitello Quarry in Tuscany (Central Italy) where birch-bark tar, found on the proximal part of two flint flakes, is dated to the end of MIS 7. Nevertheless, a peculiar feature of the Middle Paleolithic at Torre in Pietra is the continuous presence of small tool blanks on pebbles and cores and on thick flake albeit at a much lower frequency than in the older Acheulian industries. The adoption of the new technology is thus characterized by innovation combined with a degree of stability. The persistence of these habits in spite of the introduction of an innovative technique underlies the importance of cultural transmission and conformity in the behavior of Neandertals.

## Introduction

**Fig 1:**
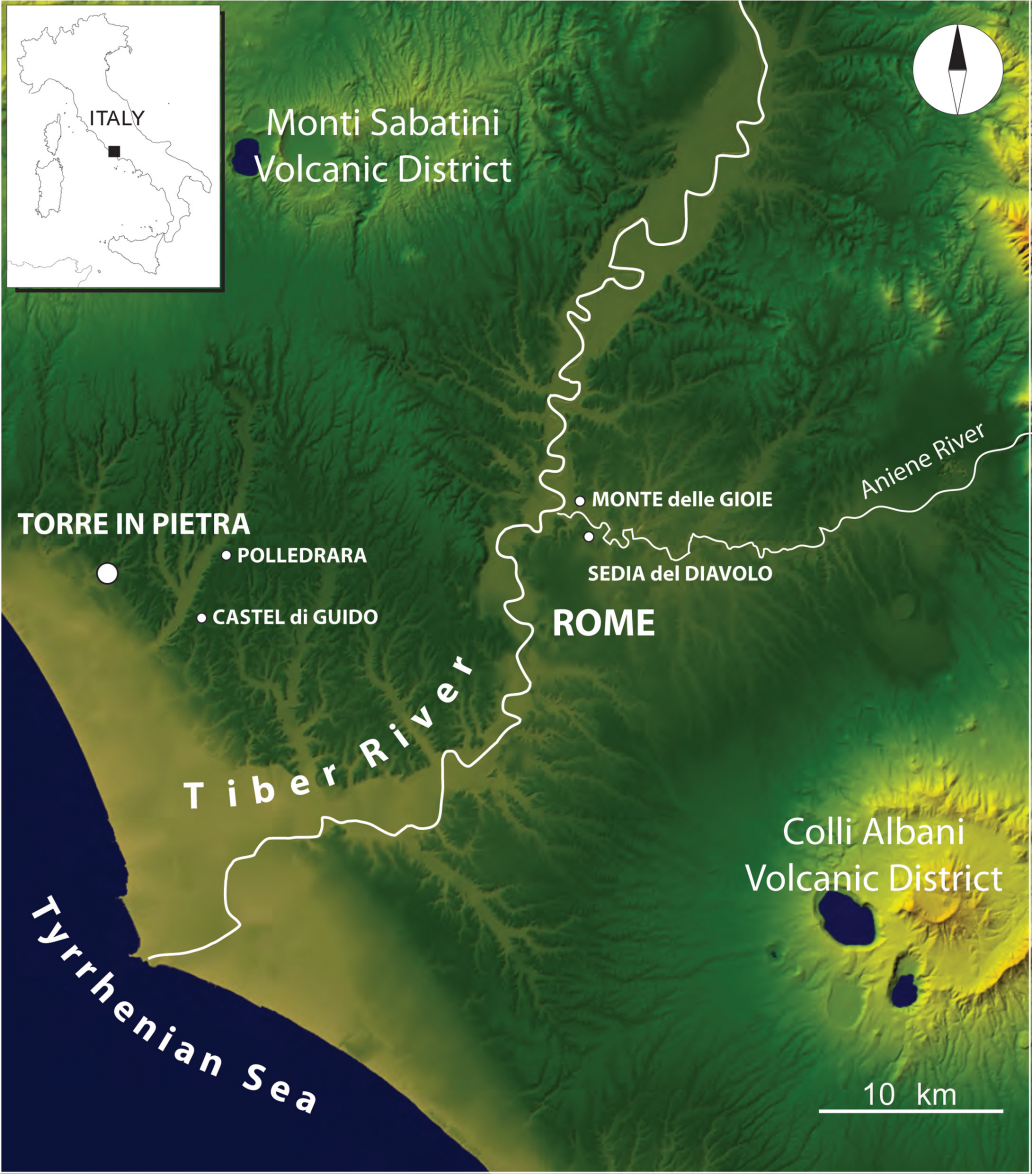
Map of Latium with location of sites mentioned in the text. Modified after Digital Elevation Map (DEM) TINITALY/01 square WA 6570, used with permission by the Istituto Nazionale di Geofisica e Vulcanologia, Rome (S4 File).

In 1954 the discovery of Acheulian artifacts and mammal remains within fluvio-lacustrine sediments cropping out at the foot of a hill in locality Torre del Pagliaccetto, 26 km northwest of Rome and 6 km from the Tyrrhenian Sea, prompted the start of excavations by AC Blanc. The site was excavated in 1954–1957, in 1963–1964 and in 1977 (Fig 1) and was more frequently reported in the literature with the alternative name of Torre in Pietra, derived from the nearby village.

In 1978 a series of papers were published in the journal *Quaternaria* under the direction of A. Malatesta covering the geology, stratigraphy, vertebrate, invertebrate and botanical remains, and the stone artifacts from the Acheulian and Middle Paleolithic deposits. In the section published by Malatesta [1] layers 12n and 11m contained Acheulian artifacts and layer 4d contained Middle Paleolithic artifacts. The size of the excavated area was 200 and 40 m^2^ respectively (Figs A-C in S1 File). The sequence of deposits with the Acheulian level at the base was referred to as the Aurelian Formation [1]. The upper deposits with the Middle Paleolithic artifacts were later related to the Vitinia Formation [2].

At the time the chronological context was provided by correlation of the local geological terms with the Alpine sequence of glacial and interglacial periods [1]. The Acheulian was estimated to be of Riss age and the Middle Paleolithic of level *d* was attributed to the Last Interglacial. Since 1980 intensive research on the area in and around Rome, based on paleomagnetism, ^40^Ar/^39^Ar geochronology, tectonic studies and reconstructions of the structural setting of the region indicates that the area has been characterized by pulses of tectonic uplift and intense volcanic activity starting at about 600 ka with a long series of eruptions from the Monti Sabatini, north of Rome, and the Alban Hills, south of Rome. The volcanic events (ignimbrites, ash-falls) interbedded with fluvial-lacustrine sections have been dated by ^40^Ar/^39^Ar. The dates show that fining-upward aggradational sequence of deposits occurred rapidly in response to sea-level changes due to glacial melting thus allowing correlations with marine isotopic stages [2–8]. At the same time paleontological studies led to the definition of new faunal units correlated to isotopic stages [9–14]. As part of a project on the Middle and Upper Pleistocene archaeological record of the Latium region (National Science Foundation Award no. 1118143) we reopened the site in 2012 (Fig 1).

**Fig 2:**
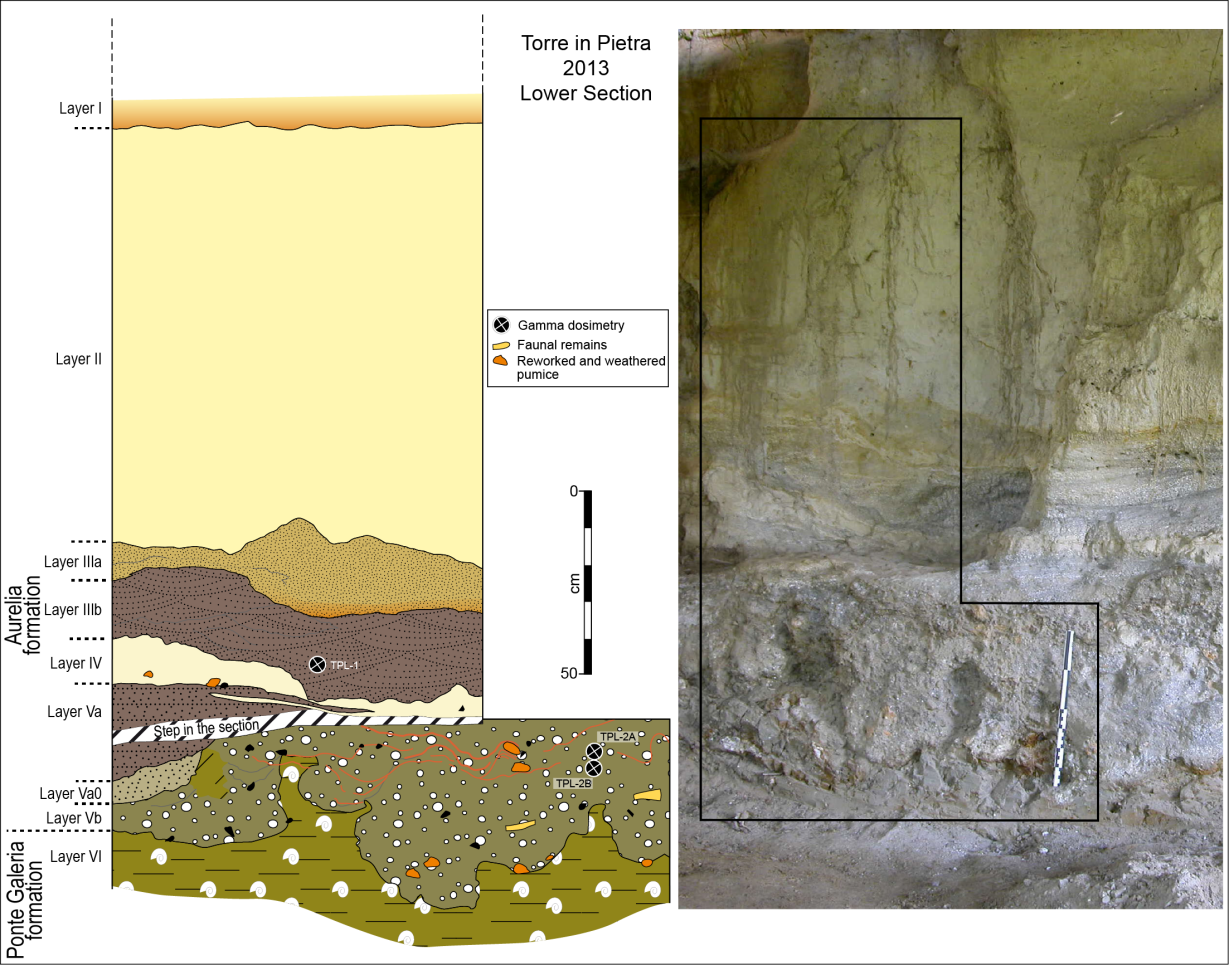
Photo and drawing of Torre in Pietra Lower Section. Photo and drawing show the refreshed section with layers labeled according to the present study.

**Fig 3:**
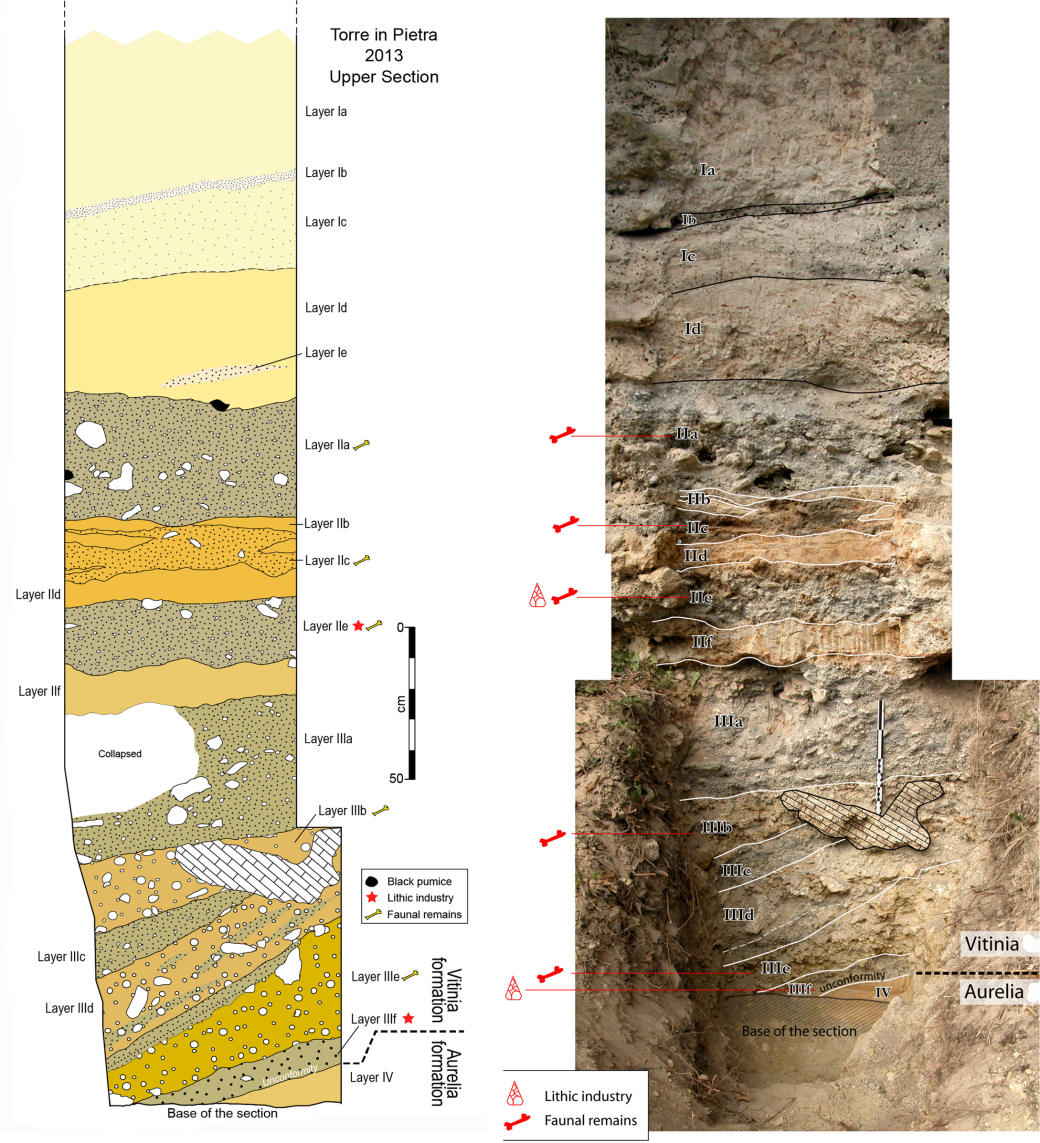
Photo and drawing of Torre in Pietra Upper Section. Photo and drawing show the refreshed section with layers labeled according to the present study.

Two stratigraphic sections called Lower and Upper, corresponding to the Aurelia and the Vitinia Formations, were cleaned (Figs 2 and 3; Fig D in S1 File), sediment and faunal materials were collected for dating and new lithic analyses were done on collections stored in the Pigorini Museum (Rome) and in the Italian Institute of Human Paleontology (Anagni and Rome).

**Fig 4:**
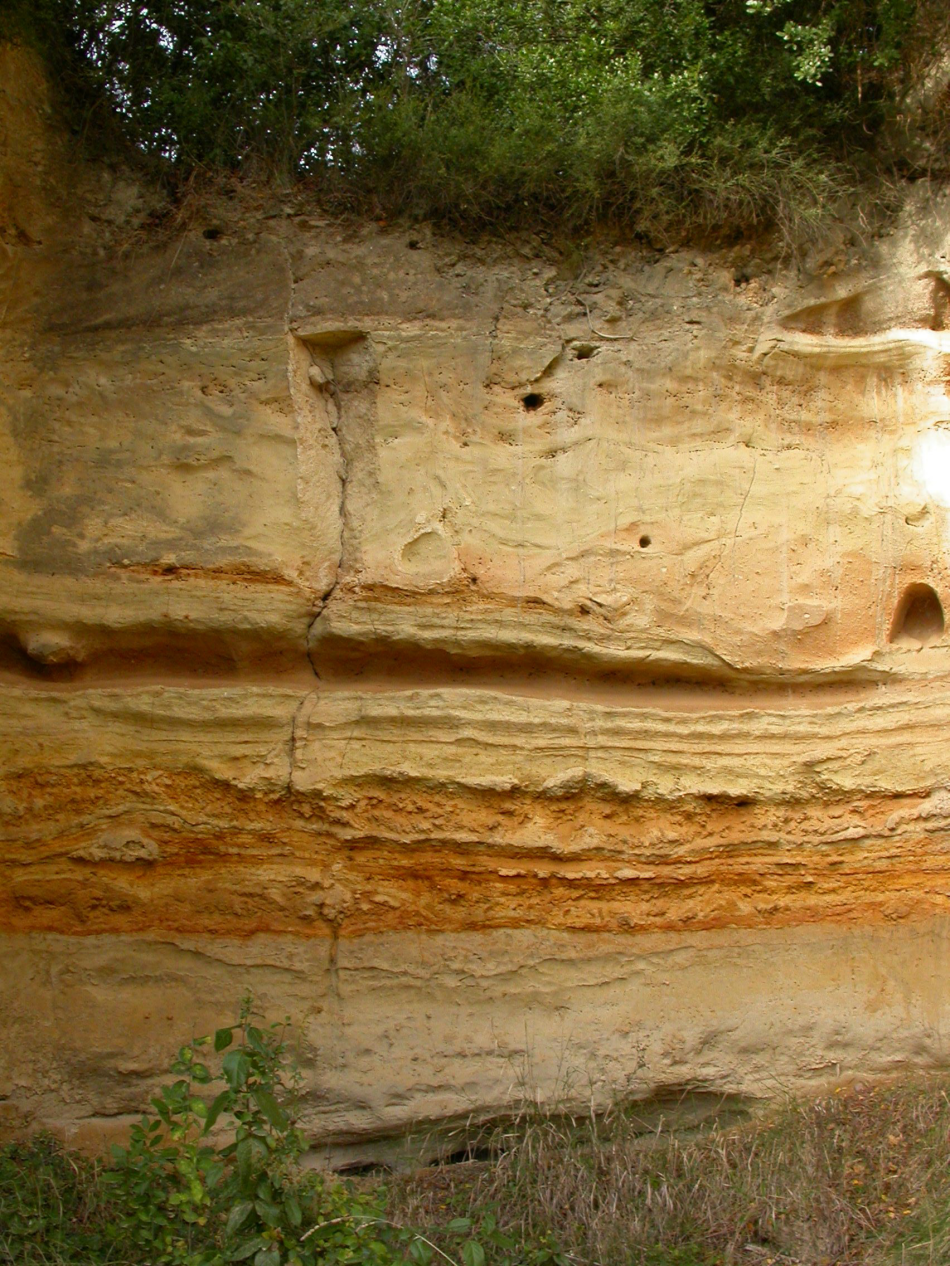
Upper part of the Aurelia Formation and base of the Vitinia Formation at the top.

The upper part of the Aurelia Formation with the base of the Vitinia Formation at the top is illustrated in Fig 4.

Since 1980 the Aurelia Formation at Torre in Pietra was correlated to MIS 9 and the Vitinia Formation to MIS 7. Nevertheless the type section of Torre in Pietra lacked geochronological constraints supporting these correlations. We have now radiometric dates and a geochronological framework that refine these correlations. Based on field observations, U-series-ESR and ^40^Ar/^39^Ar dating and geomorphologic data we present here a review of the stratigraphy, age and lithic technology of the Torre in Pietra sequence within the framework of established volcanic stratigraphy and sequence of glacio-eustatically controlled fluvio-lacustrine deposits in the Latium region.

The goals of our paper are: (a) to place the Torre in Pietra Acheulian and Middle Paleolithic in the chronostratigraphic context of the Rome region based on recently acquired geochronological data; (b) to present the results of a detailed taphonomic and technological analysis of the two lithic assemblages for a more precise characterization of their features; (c) to contribute to the general problem of cultural continuity or discontinuity between the Acheulian and the Middle Paleolithic by adopting a comparative perspective on the mode of production of small tools. The analysis of the Castel di Guido small tools served this purpose.

This paper is part of a larger study of several Lower and Middle Paleolithic assemblages in the Latium region whose aim is to build an overview of Neandertal lithic technology and its changes throughout the whole period of their presence in the region.

## Materials and Methods

### Permits and repositories

The Torre in Pietra collections are housed in the Pigorini National Museum of Prehistory and Ethnography in Rome and in the Italian Institute of Human Paleontology in the town of Anagni, Italy. Permits to study and take photos of the materials were obtained from the Soprintendenza of the Pigorini Museum (Prot. N. 212, 25.02.03/15, MBAC-S-MNPE Pigorini Cl. 25-02-04/5 and Cl. 23.03.02/1) and from Fabio Parenti, President of the Italian Institute of Human Paleontology. Permits to clean the stratigraphic sections at Torre in Pietra and to take sediment samples for dating were issued by the Soprintendenza of Etruria Meridionale, Rome (Prot.MBAC-SBA-EM N. 5374). Faunal materials for ESR and U-series dating were kindly provided by Antonio Tagliacozzo of the Pigorini Museum. The Castel di Guido collections are housed in the laboratory of Giovanni Boschian at the University of Pisa. Permissions from copyright holders of a few figures are provided in the S4 File.

### Sorting and sampling

We followed the sorting procedures used by us in the analysis of other assemblages. We select all cores, core fragments, tools, tool fragments and complete or broken flakes preserving the platform. Retouched pieces and cores are assigned individual catalog numbers, unless already assigned by the Museum or the excavator, and are individually bagged in reusable zipper bags (Minigrips) with pre-printed labels. Our sorting procedures exclude flake fragments (broken flakes without the platform) flakes < 15 mm and chunks from technological analysis; these materials are bagged by large categories and remain available for specific studies. Further details on sorting are provided in the taphonomy section of each assemblage.

### Methods

**Fig 5:**
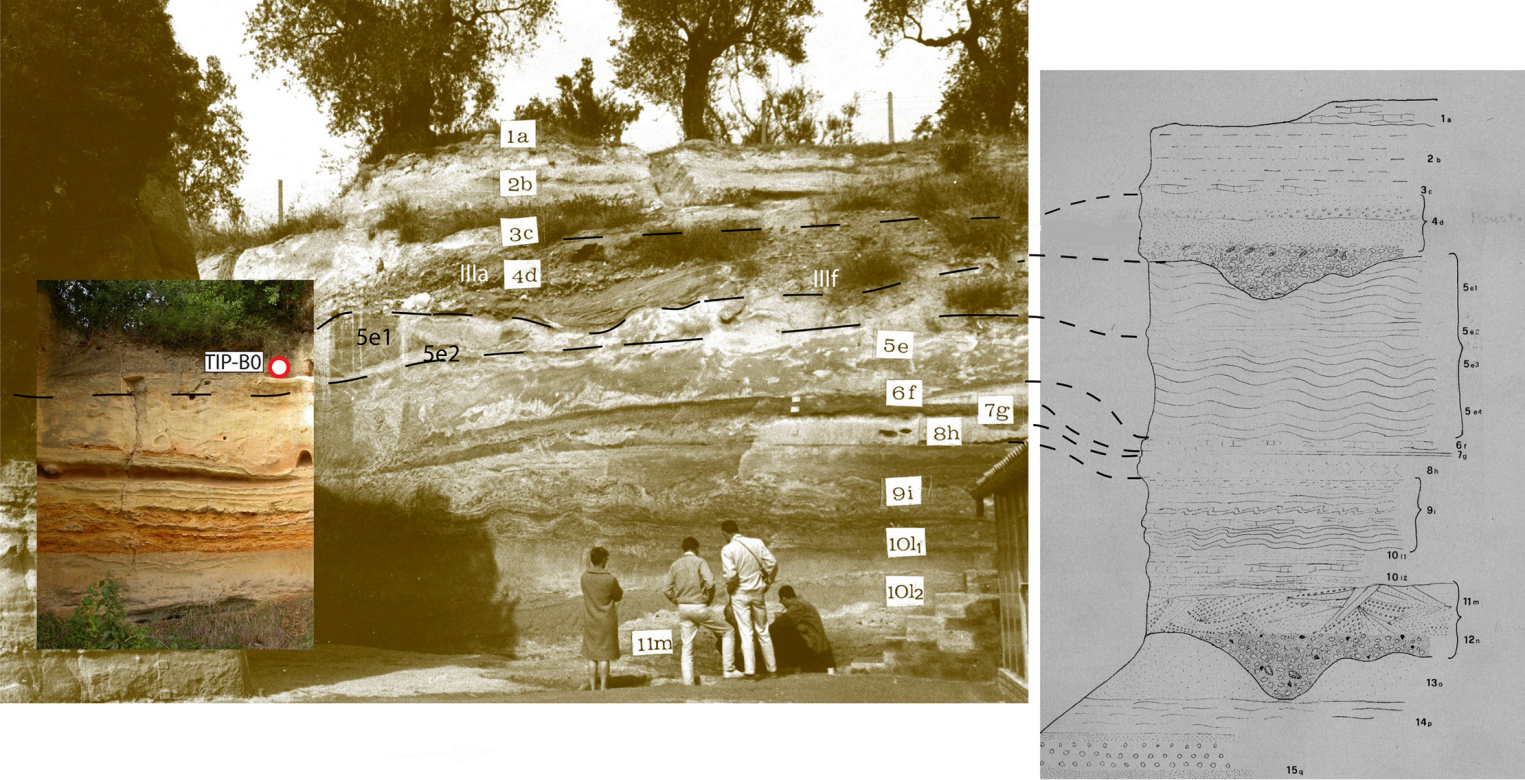
Stratigraphy of the main Torre in Pietra section. Center: the main section of excavations carried out between 1955 and 1964; (left) upper part of the Aurelia Formation with position of dating sample TiP-B0; (right) correlation with layers as drawn by [1]. The center and right panels of the figure are reprinted from [1] with permission by the Italian Institute of Human Paleontology (S4 File).

**Fig 6:**
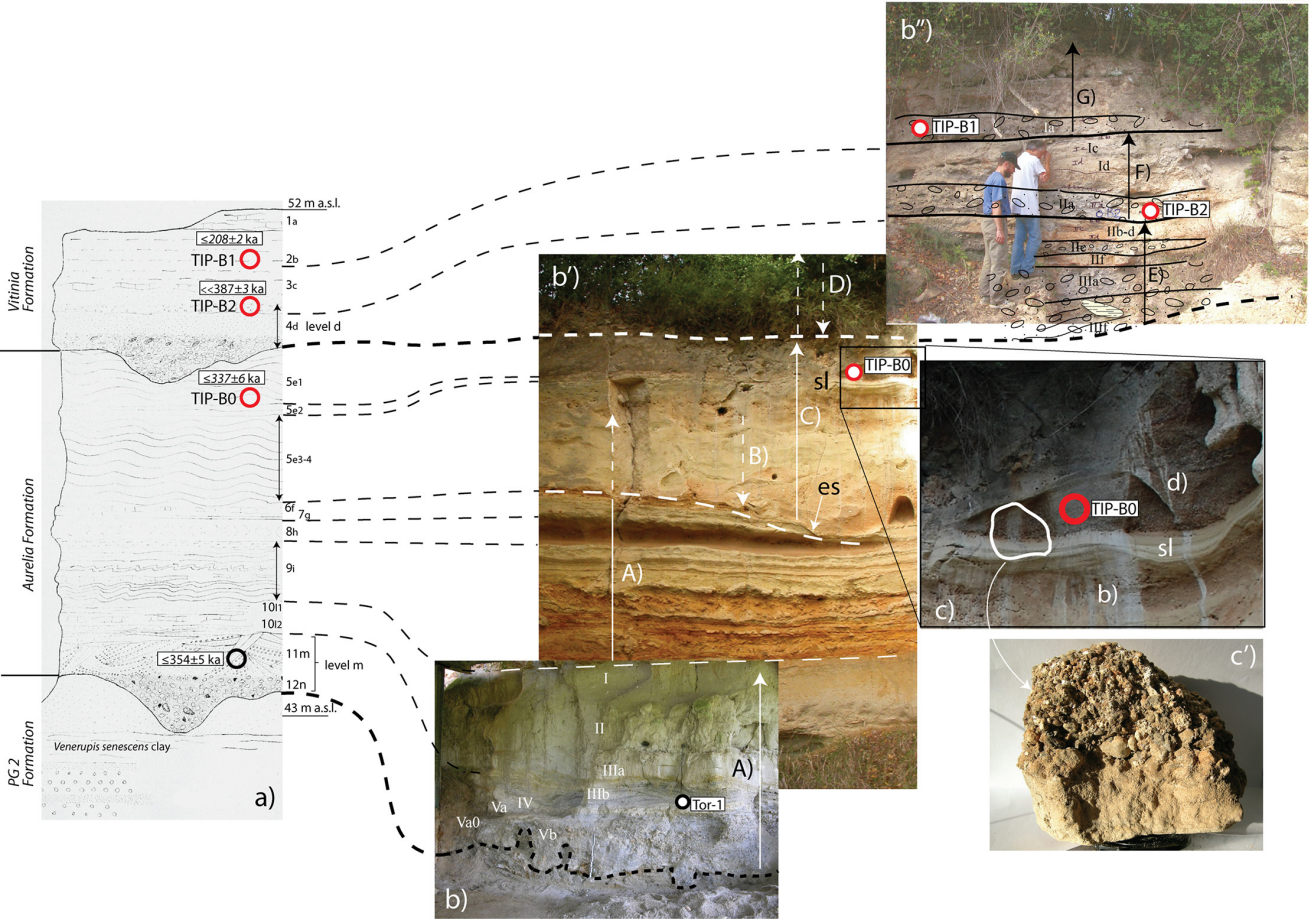
Composite stratigraphy of the Torre in Pietra sections. Photographs show the refreshed Upper and Lower sections with layers labeled according to the present study and with position of the samples dated by ^40^Ar/^39^Ar method (red circles: this work; black circle: [24]). Layers as labeled in [1] are reported besides the stratigraphic scheme, along with the revised elevations, as inferred from the topographic survey in [29] (Fig C in S1 File). Capital letters indicate phases of sediment aggradation (A, C, E, F, G) and intervening regressive phases (B, D), corresponding to as many sea-level oscillations. The left panel of the figure is modified after [1] with permission by the Italian Institute of Human Paleontology (S4 File).

The study of lithic assemblages is based on quantitative and qualitative analyses including metrical, technological and typological attributes combined with particular attention to the sequence of manufacture. It is preceded by a discussion of the site context and taphonomy. Figs 2, 3, 5 and 6 show the refreshed sections with layers labeled according to the present study and location of dating samples. Analytical details of dating methods are provided in S2 and S3 Files. The analytical criteria used in the technological and taphonomic analyses of the lithic artifacts are described in the S5 File.

## Results

### Stratigraphy of the Torre in Pietra site

At Torre in Pietra the late Middle Pleistocene stratigraphic sequence is about 9 m thick resting unconformably on the Ponte Galeria Formation (PGF). The Ponte Galeria Formation [2] represents the delta system of the ancient Tiber (Paleotiber) River. It includes the PG1, PG2 and Santa Cecilia Formations which are correlated to MIS 19, MIS 17and to MIS 15. The upper part of the PGF, directly under the Acheulian layer (Fig 2) is made of clays with littoral shells (*Cerastoderma* and *Venerupis senescens*) reflecting lagoonal deposits. Elevation of this clay section between 40 and 43 m above sea level (a.s.l.) corresponds to that of the clays with *Venerupis senescens* in the PG 2 Formation that are correlated with MIS 17 by combined paleomagnetism and ^40^Ar/^39^Ar [5–6, 15–16].

Since deposition of the PGF, other six major aggradational successions deposited in the coastal area during periods of sea-level rise in response to deglaciations during MIS 13 through MIS 1, plus a minor succession correlated with sub-stage MIS 8.5 [17]. These aggradational successions are designated by the formation names of Valle Giulia (MIS 13), San Paolo (MIS 11), Aurelia (MIS 9), Vitinia (MIS 7), and Epi-Tyrrhenian (MIS 5), whereas that correlated with MIS 8.5 is informally named Via Mascagni succession ([18], and references therein).

Since deposition of the Vitinia Formation, about 60 m of uplift occurred in the coastal area, followed by erosion [19]. As a consequence of this uplift, the marine terraces of MIS 7 and MIS 5 are found around 56 m a.s.l. and around 36 m a.s.l., respectively, along the coast of Latium north of the Tiber mouth [18].

At Torre in Pietra, the Aurelia Formation overlies PG2 with an erosional contact (Fig 2). At its base a sandy conglomerate and the cross-bedded sands above it contain Acheulian bifaces and other lithic and faunal remains, including bivalves from the underlying levels (Fig E in S1 File). The sandy conglomerate also contains reworked and very weathered pumices which were interpreted as deriving from the Red Tuff with black scoria (Tufo Rosso a Scorie Nere). The Red Tuff is an ignimbrite representing an eruptive event of the Monti Sabatini north of Rome, dated to 449 ± 1 by ^40^Ar/^39^Ar [20] in good agreement with previous results [21, 22]. The Red Tuff outcrops about 1 km north of the site, at slightly higher elevation, see geologic map in [23]. The reworked pumices clearly date the eruptive event, not the younger archaeological level. However, a more recent age determination on a sample collected in the volcanoclastic cross-bedded sands above the sandy conglomerate was performed by the ^40^Ar/^39^Ar method on sanidine single crystals allowing to identify a youngest population of 3 crystals yielding a weighted mean age of 354±5 ka (2 standard deviations (SD) analytical uncertainty) [24] (see section “^40^Argon/^39^Argon dating”) against an older population of 7 crystals yielding 453±4 ka (2 SD analytical uncertainty). Besides indicating a possible oldest age within MIS 10, the lack of crystals younger than 354±5 ka within this sample suggests that deposition of the sediment containing the archeological findings occurred not much later, otherwise reworking and incorporation of younger eruptive products would have been probable.

Above the cross-bedded sands of layer 11 *m* there are about five meters of silts and clays with diatoms, fresh-water gastropods and ostracods indicating a lacustrine to marshy deposit, without any artifacts (Figs 4 and 5). According to a supposed age not much younger than 354±5 ka, the sedimentary cycle is referred to as the Aurelia Formation which is correlated with MIS 9 [23, 24] and represents the type-section for the "Torre in Pietra" faunal unit [25].

The faunal association of Torre in Pietra is found in broadly contemporaneous sites such as Castel di Guido and La Polledrara [9]. However, in this last location the faunal assemblages and the lithic industry occur within a sin-eruptive lahar deposit dated 325±2 ka (2 SD analytical uncertainty) and overlying fluvial sands containing reworked volcanoclastic material providing a youngest age population of 359 ± 6 ka (2 SD analytical uncertainty) [24, 26]. Therefore, the lithic assemblage is about 30 ka younger than that recovered at Torre in Pietra and occurs in a different depositional context. The lithic assemblage of Castel di Guido is found in a stratigraphic-depositional context showing similarity to that of La Polledrara ([27, 28], ongoing research]).

Within the Aurelia Formation a poorly marked erosional surface occurs above layer 6f of [1] ('es' in Fig 6B') and is followed by a massive sandy clay deposit (layer 5e3-4) comprising coarse clastic material, mainly fragments of calcareous sediment, likely eroded from the underlying strata of the Aurelia Formation, suggesting the occurrence of a minor sea-level fall (B, in Fig 6B') prior to its deposition. A ca. 10 cm thick, laminated white silt layer (layer 5e2; sl in Fig 6B') occurs above the massive deposit, indicating conditions of very still water, possibly corresponding to a period of stasis following the new sea-level rise (C, in Fig 6B').

Above this fine-grained layer is a 50 cm-thick, clino-stratified deposit (layer 5e1) entirely composed of mm- to cm-sized dark grey volcanic scoriae, subordinate, mm-sized white pumices, sparse leucite (often analcimized) and sanidine crystals. We collected one sample (TIP-B0) for ^40^Ar/^39^Ar dating in this layer (Fig 6C-c').

A major unconformity above this volcanoclastic level marks the passage to a new aggradational cycle, constituted by a repetition of three fining-upward successions with a coarse gravel layer at the base of each one (layer Ia, IIa, IIIa/f in Fig 6B"), suggesting the occurrence of a marked sea-level fall (D in Fig 6B'), followed by as many sea-level oscillations (E, F and G, in Fig 6B").

Overall, these deposits represent a second aggradational succession above that correlated with the Aurelia Formation, and were attributed to the MIS 7 Vitinia Formation [9]. We collected two other samples (TIP-B1, TIP-B2) for ^40^Ar/^39^Ar dating from thin layers enriched in volcanic material occurring within the uppermost two gravel layers (Ia and IIa, Fig 6B"). An early Middle Paleolithic industry occurs mostly at the base of the deposits of the Vitinia Formation [29] between layers IIIf and IIe of Fig 3.

### Geomorphological setting of Torre in Pietra

**Fig 7:**
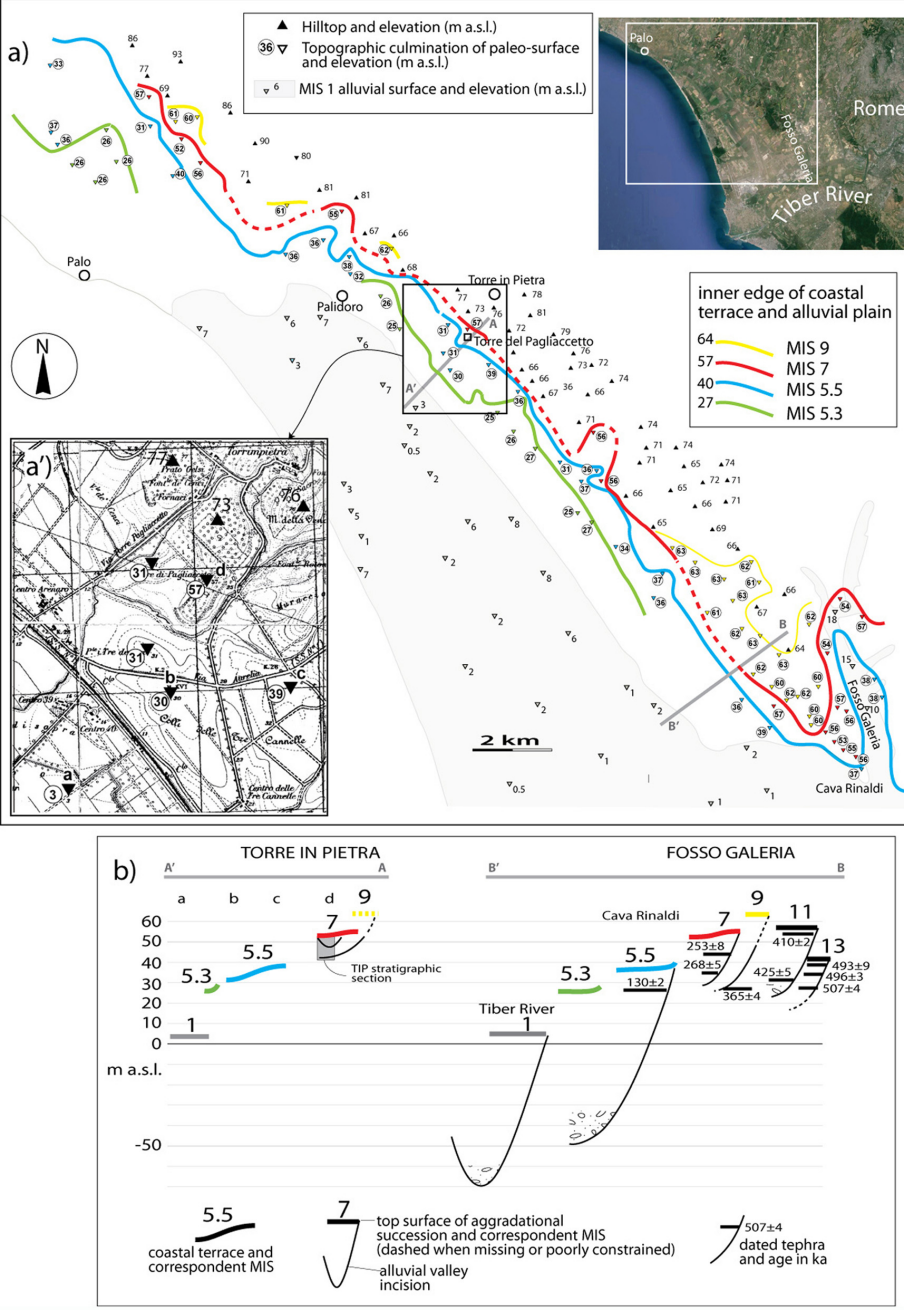
Reconstruction of the coastal terraces and their inner edges in the sector north of the Tiber mouth. a) The reconstruction is based on mapping of a set of flat surfaces identified by topographic points (triangles) selected on the 1:25.000 topographic base, as in the detail of inset a') and following criteria described in the text. b) Cross section A-A' shows the morpho-stratigraphic setting in Torre del Pagliaccetto through the coastal plain with elevation of the terraced surfaces correlated with the high stands of marine isotopic stages (MIS). Composite cross-section B-B' shows the complete suite of aggradational successions and related terraced surfaces in the Fosso Galeria area, whose correlation with the different MISs is provided by ^40^Ar/^39^Ar ages of intercalated tephra layers and morpho-stratigraphic relationships [5, 18, 31]. The inset top right is a satellite image from Google Earth. Inset a' is modified from the 1:25 000 topographic map of the Istituto Geografico Militare d’Italia, Foglio 149, survey of 1949.

Recently acquired geochronological and stratigraphic data allowed Marra et al.[18] to recognize the occurrence of the MIS 5 aggradational succession forming a fluvial terrace ca. 37 m a.s.l. at Cava Rinaldi, in the Fosso Galeria valley near the coast of Rome (Fig 7). The authors correlated this terrace with a paleo-surface occurring around 40 m a.s.l. along the coast and identified it as the marine terrace of MIS 5.5, revising previous attribution to MIS 7 [19, 30].

In the present work we use the geochronologically constrained terraced deposit of Cava Rinaldi as a reference point to re-assess the chronologic relationships among the different terraced surfaces correlated to highstands of MIS 5, MIS 7 and MIS 9 in the coastal area between Cava Rinaldi and Torre del Pagliaccetto (Fig 7). We mapped coastal terraces following a simple geomorphological approach, based on the identification of a set of flat surfaces characterized by elevation lower than that of the hilltops bordering the coastal plain to the NE (black triangles in Fig 7A'), and ranging few meters around a mean value. The selected topographic culminations of the reconstructed terraced surfaces include all the hilltops (i.e. each elevation point within a closed, 5 m spaced isoline), and other quasi-equivalent points within almost closed isolines bordering plateau-like sectors, as in the example of inset a' in Fig 7A, showing the Torre in Pietra area. The inner (landward) edges of the coastal terraces have been also reported in Fig 7A. Using geomorphologic and geochronological data acquired in the present study, we reconstruct a cross-section perpendicular to the coastline (A-A') in Torre del Pagliaccetto, to compare the better geochronologically constrained, similar cross-section in Fosso Galeria (Fig 7B). We reconstruct this second composite cross-section by projecting on a NE-SW transect (B-B') the suite of aggradational successions and their top surfaces identified based on stratigraphic and geochronological data [14, 18, 19, 24].

### Chronometric dating and marine isotope correlations

#### ^40^Argon /^39^Argon dating

**Fig 8:**
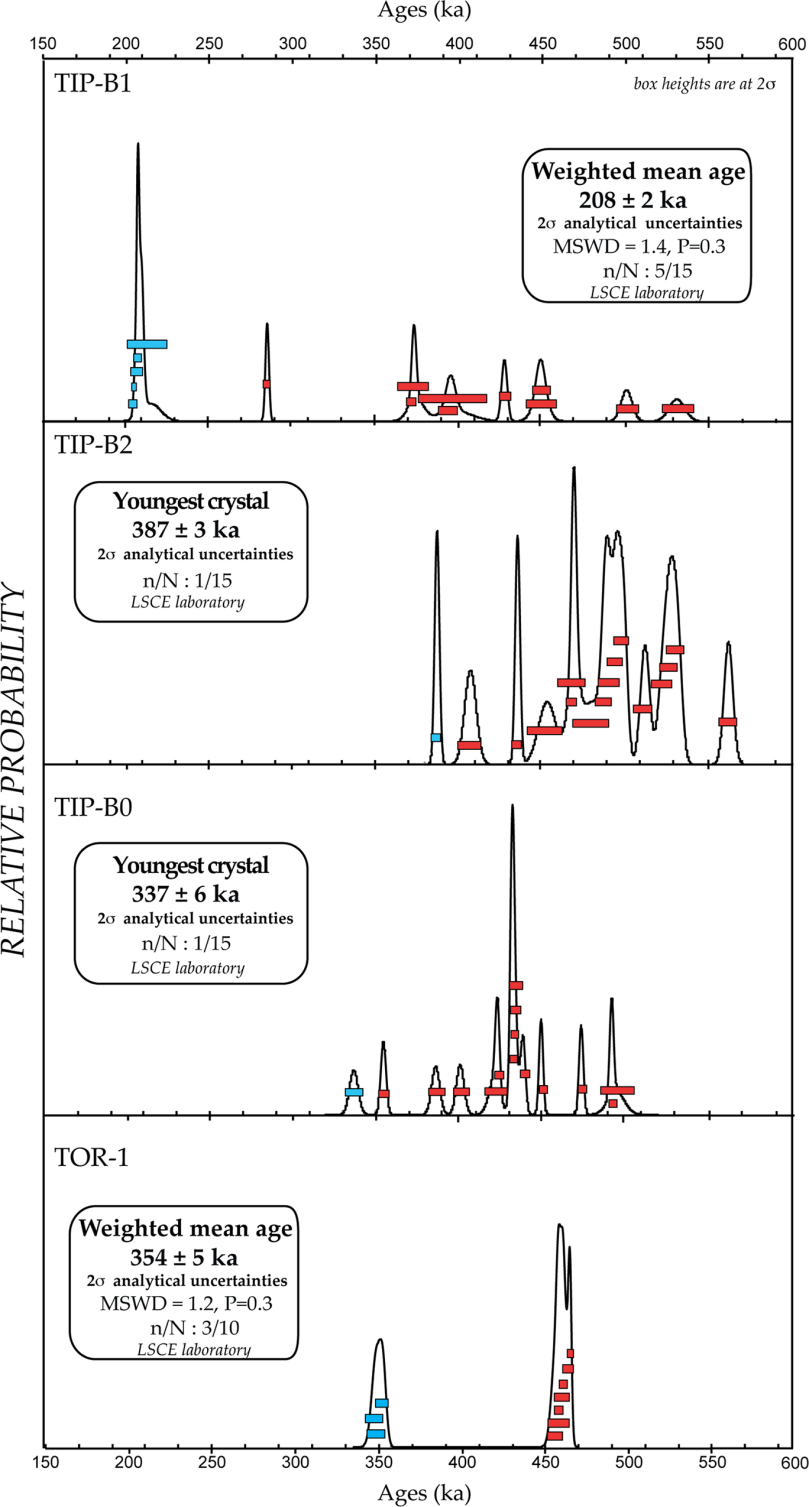
^40^Ar/^39^Ar dating. Results are presented as probability diagrams [32] for levels TOR-1, TIP-B0, TIP-B2 and TIP-B1. Each bar corresponds to the age obtained for an individual dated crystal with the corresponding analytical uncertainties at 95% of probability. MSWD = Mean Square Weighted Deviation, P = Probability fit, n/N = number of grains taken into account to calculate the weighted mean age on the total number of dated grains.

Full analytical details for individual crystals from each level are given in the supplementary dataset (S2 File). In Fig 8 results are presented for each level as probability diagrams [32]. For reworked volcanic material, the youngest crystal population corresponds to the age of the last volcanic eruption recorded in the deposit or to the last source of reworked material. A homogeneous population is considered relevant when the weighted mean of these crystals has the following statistical characteristics: MSWD < 1.5, Probability fit ≥ 0.1. The weighted average ages are calculated using IsoPlot 3.00 [33] and given at 2 SD (95% of probability) including J uncertainty throughout the text. As the ^40^Ar/^39^Ar ages will be compared to another method we also indicate the full external uncertainties within brackets hereafter.

TOR-1. Ten crystals were analyzed for level TOR-1 that corresponds stratigraphically to the archaeological level “*m*”. The probability diagram obtained demonstrates two main crystal populations. The oldest, constituted by seven crystals, is dated around 453 ka and the youngest one provides a weighted mean age of 354 ± 5 ka (7 ka), MSWD = 1.2 and P = 0.3.

TIP-B0. Fifteen crystals were analyzed for this unit. The probability diagram obtained is multimodal, as expected for this kind of fluvial layer, showing an important reworking of the included volcanic material. The ages range from 473 ± 3 ka to 337 ± 6 ka (Fig 8). Only one crystal is dated at 337 ka. This result is thus preliminary. More analyses are needed to confirm the presence of a volcanic eruption dated around 335 ka in this layer.

TIP-B2. Sixteen crystals were analyzed for level TIP-B2. The probability diagram is multimodal as expected for reworked volcanic material. At least nine different eruptions are recorded in this level and the youngest one is represented by only one sanidine crystal dated at 387 ± 3 ka.

TIP-B1. Fifteen crystals were measured for TIP B1. The probability diagram is multimodal, as eight different crystal populations are highlighted. The youngest population recorded constituted by five crystals, is centered at a weighted mean age of 208 ± 3 ka (6 ka, MSWD = 1.4 and P = 0.3), (Fig 8). The ^40^Ar/^36^Ar initial intercept calculated using this population is not precise (303 ± 6, Table C in S2 File) but still equivalent within uncertainties to the atmospheric ratio of 298.56.

**Fig 9:**
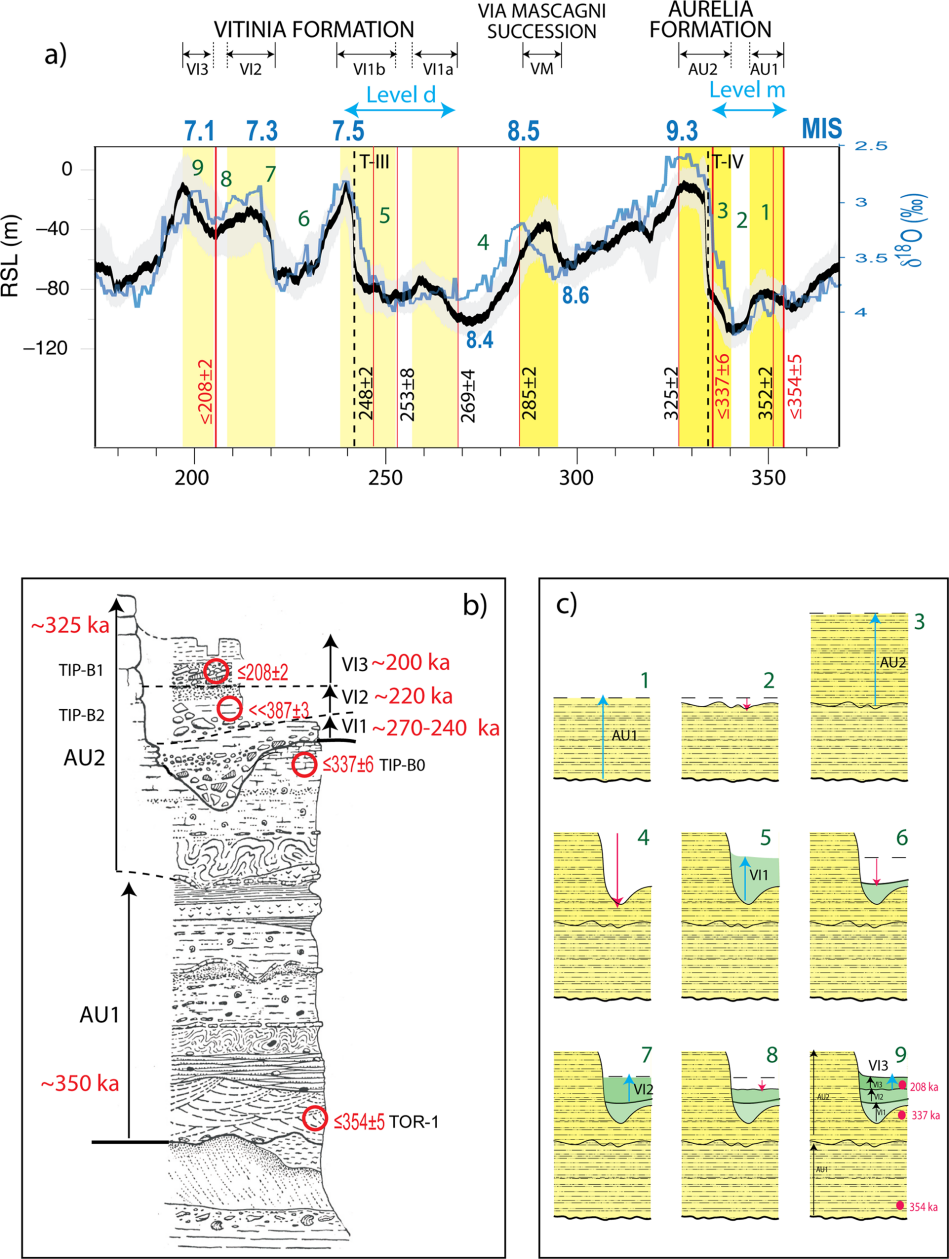
The Torre in Pietra succession within its geochronological framework. a) Phases of aggradation in the coastal area of Latium (yellow boxes) with their geochronological constraints (red vertical bars representing the weighted mean ^40^Ar/^39^Ar ages of volcanic deposits intercalated within the sedimentary successions, ([4, 24, 26] and this work) are compared with independently dated phases of sea-level rise (RLS curve by [39] and with an astrochronologically calibrated δ^18^O curve [40]. b) Based on the age constraints provided by samples dated in this work and previous work (red circles), and according to [24] who defined early aggradation phases preceding glacial termination four (T-IV) for the Aurelia Fm (AU1), we interpret the two aggradational cycles below the major unconformity as the complete sedimentary record of the Aurelia Formation, with the second cycle (AU2) matching the sea-level rise during glacial termination IV. Similarly, we correlate the three fining-upward successions with gravel at the base recognized in the Torre in Pietra section (VI1,VI2,VI3) with as many sea-level oscillations leading to the deposition of the aggradational successions of the Vitinia Formation. Stratigraphic section from [41]. c) Reconstruction of the aggradational and erosional phases leading to the deposition of the different members of the sedimentary succession of Torre in Pietra. See description in the text; numbers also indicate corresponding phases of sea-level fluctuation as detected by RSL curve in Fig 9A.

Discussion. ^40^Ar/^39^Ar dating is performed on volcanic crystals which provide direct age for the sedimentary strata only if they are the juvenile fraction of a primary deposit. Otherwise the youngest crystal population provides a post-quem age. A very poor fraction of evidently reworked volcanic crystals is present in level *d* and just above (sample TiP B2). In contrast, volcanoclastic materials appear to be less reworked in samples TiP B0 and TiP B1 which are below and above level *d*, respectively (Figs 6A and 9B). Level *d* is thus constrained by a date directly below and one above it, which, combined with comparisons of the aggradational history of the succession and the oxygen isotopes timescale provide an excellent age constraint (see below “Geochronological and morpho-stratigraphic constraints”).

### Uranium-series and ESR dating

Samples. Seven teeth (lab numbers 3448 to 3454) were analyzed by U-series and ESR and an additional tusk fragment (3455) by U-series. Samples 3448, 3451, 3452, 3453, 3454 are from level *d* and samples 3449, 3450 and 3455 were from level *m*. Analytical details for individual samples from each level are provided in the S3 File, Tables A-C. In Tables B and C of the S3 File age estimates in agreement with ^40^Ar/^39^Ar results and other independent lines of chronological evidence, discussed in the text, are indicated in bold green.

Uranium-series dating. U-series results on bones have generally been regarded as minimum age estimates. This derived from the fact that modern bones and teeth are virtually U-free while fossils contain elevated U concentrations. There are two models that account for U-uptake, the diffusion-adsorption (DA) model [34] and the diffusion-adsorption-decay (DAD) model of [35]. The difference is that the former is based on closed system age calculations while the latter assumes a continuous U-diffusion. While the age difference in young samples (<150 ka) are minor (Table B in S3 File), for older samples the age differences increase. A consequence of the DAD assumptions is that any ^230^Th/^234^U ratio of >1 would indicate leaching (Table B in S3 File). However, we have observed that virtually all older samples with ^234^U/^238^U ratios of >1 have also ^230^Th/^234^U ratios of >1 yielding finite closed system results, but implying leaching under DAD conditions. This is a strong indication that it is unlikely that continuous U-diffusion persists over several hundreds of thousands of years. Nevertheless, both open system models cannot recognize a longer period of little U-uptake followed by a more pronounced U-uptake, as it often seems to be the case for open air sites. Several samples show clear signs of U-leaching: e.g., the cement layer of sample 3449 and the dentine of sample 3454. While most analytical spots of the other samples yield finite close system age estimates, many age calculations based on continuous diffusion assumptions would indicate leaching. As we regard it unlikely that the dental tissues remained an open system for more than 200 ka, we only discuss the closed system age results. In contrast to bones, it is not possible to apply DA or DAD calculations to the U-series data sets because the diffusion directions cannot be established. Variations in the analytical results from spot to spot are most likely due to U-micro diffusion processes [36]. To account for this, we calculated integrated ages for the various dental tissues (lower rows in the individual data tables). To gain further insights into U-migration and the meaning of the calculated U-series age estimates, the results obtained on the various skeletal tissues are compared with the best independent age estimates for layer *d* (240 to 270 ka) and *m* (around 350 ka).

Level *d*. Sample 3448: The dentine is close to equilibrium. The closed system assumptions yield an age of 380±48 ka while open system assumptions indicate leaching. There is a possibility that some leaching has occurred (analysis #18). Due to a much delayed U-uptake into enamel, these results are significantly younger than the dentine results (108 to 147 ka). When compared to the best age estimate for layer *d*, the dentine has clearly experienced leaching overall while the diffusion into the enamel was delayed.

Sample 3451: One dentine analysis (#4) indicates leaching. Both dentine and cement yield closed system ages of around 250 ka (in agreement with the independent age estimates) the enamel ages are, as expected, significantly younger (108 to 127 ka)

Sample 3452: Both cement layers and one dentine section yield results of around 270 ka, while another dentine section an age nearly 100 ka older. The enamel sections yielded ages of around 136 to 164 ka.

Sample 3453: The dentine yielded 254±15 and enamel 135±22 ka.

Sample 3454: The dentine shows massive leaching while the enamel yielded an age of 235±23 ka.

Without independent age control it is difficult to ascertain which dental tissues show leaching and which ones do not. Based on the analytical results, none of the enamel layers show any signs of leaching, thus these results can be regarded as minimum age estimates. If all teeth are contemporaneous with the deposition of level *d*, then the enamel of 3454 would indicate a minimum age of around 235 ka. This is indeed close to the independent age estimate for layer d. Furthermore, most of the other dental tissues that show no indication of leaching, the cement and dentine of 3451, the cement of 3452, and the dentine of 3453 show closed system age results that are also in agreement with the independent age control, implying a fast uptake period followed by closed system behavior.

Level *m*. Sample 3449: The outer cement layer shows leaching. The dentine yielded 240 ka and the enamel had such low U-concentrations that the U-series ages are not meaningful.

Sample 3450: The dentine yielded 216 ka and the enamel had such low U-concentrations that the U-series ages are not meaningful.

Sample 3455: This fragment possibly of an Elephas tusk, shows some secondary overprint on spots #1 to #3. The other data yield higher results. The averages excluding the outer portion as well as for the whole bone agree with the independent age estimate for layer *m*.

The teeth yield very similar results to those from level *d* with dentine ages of around 240 ka. This points to a phase of U-mobilization around 240 ka where the main U-accumulation into the dental tissues of both layers took place. This is probably related to a pluvial period during MIS7.

ESR dating. ESR and U-series data were combined to calculate U-series-ESR (US-ESR) age estimates [37] and closed-system U-series/ESR (CSUS ESR) age estimates [38]. In addition, we also calculated early uptake age estimates (these provide a minimum age estimates). The data are shown in S3 File, Table C.

All samples from level *d* did not yield US-ESR age estimates, because the U-series results were older than the ESR estimates. For level *m*, sample 3449 yielded 374 +46/-36 ka and sample 3450 402 +30/-22 ka. The ESR data are peculiar. The dose values of most samples from level *d*, except 3451, are around 1000 Gy, while the samples from level *m* yield dose values around 1700 Gy, as could be expected if both sample sets had similar total dose rate values. However, the younger samples have significantly higher U-concentration in the enamel than the older samples. This results in high internal dose rates, which do not allow any solutions for the US-ESR model. When calculating closed system U-series ages, this model yields the oldest possible age for a given U-series/ESR data set, only sample 3451 yields a sensible result while the other yield nonsensical results, some even negative ages. In this case, the dose calculated from the U-series data is larger than the one measured in the sample. This points to serious problems, which are probably related to (i) the high U-concentrations in the enamel (e.g. the alpha efficiency could have changed) and (ii) to leaching processes. The EU age calculations give minimum ESR age estimations. Four of the samples give surprisingly close results around 160 ka whilst 3451 yields a reasonable result of 225 ka. The ESR results imply an age of > 160 ka for layer *d* (EU age calculations). However, the EU calculations do not take account of the measured ^230^Th/^234^U ratios.

Discussion. Generally, the combination of U-series and ESR yields the best age estimates for teeth. Indeed, where these calculations are possible (samples 3449 and 3450), the results agree within error with the independent age estimates. For the rest of the samples for which these calculations were not possible, the closed system age calculations should yield minimum age estimates for the respective layers, which holds true for all samples. However, while sample 3451 approximates the expected age range, all other results are significantly younger.

#### Geochronological and morpho-stratigraphic constraints

The reworked feature of the volcanoclastic material sampled within layer *m* is evidenced by the occurrence of two crystal populations yielding ages of 453±4 ka and 354±5 ka, respectively. Consistent with occurrence of materials spanning the 450–350 ka interval, ESR dating of two samples collected from layer *m* yielded 402 +30/-22 ka and 374 +46/-36 ka (S3 File, Table C). However, the combined ^40^Ar/^39^Ar and Uranium-series dating support an age close to 350 ka for the deposit of level *m* at the base of the Torre in Pietra succession. These results provide correlation with the aggradational phase occurring during glacial termination IV at the onset of MIS 9 that lead to deposition of the Aurelia Formation (AU1 in Fig 9B).

Consistent to this observation, an early aggradation of the Aurelia Formation since 352±2 ka has been suggested by geochronological constraints provided to the sedimentary succession in Rome [24], whereas an ante-quem is provided by the age of 325±2 ka (6 ka) for the sin-eruptive lahar deposit that overlies the fluvial deposits correlated with the Aurelia Formation in Polledrara [26]. Therefore, in Fig 9 we have constrained the aggradational phase of the Aurelia Formation between 354±5 and 325±2 ka (6 ka) and we suggest an age comprised between 354±5 (7 ka) and 334 ka (age for glacial termination IV based on the RSL curve by [39] Fig 9A) for level *m*.

The age distribution of sample TIP-B0 collected below the basal layer of the second aggradational succession at Torre in Pietra, with a youngest crystal yielding 337±6 ka, remarkably corresponding to that of glacial termination IV (Fig 9A), may be suggestive of correlation with the main aggradational phase of the Aurelia Formation during MIS 9, thus providing evidence of a two-stepped sea-level rise occurring since 352±2 ka, as suggested in [24].

The age of the youngest crystal population from sample TIP-B1 evidences that the upper portion of the second aggradational succession must be emplaced after 208 ± 2 ka (6 ka) (Fig 9), providing a good match with the last and more pronounced peak of sea-level during MIS 7 (i.e. MIS 7.1, [39], Fig 9A). Combined with the lack of any marked unconformity within the upper pack of sediments, despite the occurrence of three small fining-upward sequences with coarse deposits at the base (VI1, VI2, VI3 in 9b), the young age of the uppermost one of these sequences suggests that they are deposited in response to as many, closely spaced and nearly equivalent oscillations of the sea-level such as those occurring during MIS 7, marked by isotopic sub-stages 7.5, 7.3, and 7.1 (Fig 9A). Therefore, we suggest an age ranging 270–240 ka for the archaeological level *d*, which comprehends the layers IIIf-IIIa and IIe.

Remarkably, U-series ages on dentine of three samples collected within layer *d* span ~270–250 ka, with sample 3451 providing a reasonable ESR age of ~225 ka (S3 File, Table B), in good agreement with the inferred age based on correlation with the δ^18^O chronology.

Attribution to MIS 7 of the entire pack of sediments between 48 and 52 m a.s.l. above the marked unconformity at the top of the Aurelia Formation, is consistent with the morphostratigraphic setting reconstructed in this area (Fig 7B), accounting for a MIS 7 coastal terrace around 56 m a.s.l. Moreover, the stratigraphic scheme by Biddittu et al. [41] in Fig 9B shows that the upper portion of the sedimentary cycle we correlate with the final stages of aggradation of the Aurelia Formation during MIS 9 reaches higher elevation with respect to the top of the Vitinia Formation, in agreement with coastal terrace reconstruction in Fig 7B. Finally, elevation of the sedimentary succession attributed to the Vitinia Formation well above the coastal terrace of MIS 5.5, whose inner edge occurs 40 m a.s.l., rules out any possible correlation with the younger Epi-Tyrrhenian Formation (MIS 5.5) [18]. Consistent to this evidence, U-series ages on dentine from level *d* span 270–335 ka, while ESR results imply an age >160 ka, therefore also excluding correlation with MIS 5 (130–80 ka).

### The Acheulian industry of Torre in Pietra

#### Taphonomy and sampling of the level *m* artifacts

A certain number of bifaces (18 on a total of 51) were kept on display at the Pigorini Museum in Rome and were unavailable for study. We excluded from analysis other large tools that were too abraded or broken for correct diagnosis, that is eight cobbles and natural pieces with natural damage or only few retouch scars and two probable biface roughouts. We counted 18 biface shaping or possibly shaping flakes.

The distribution plan of artifacts [29] contains only part of the materials since smaller lithic pieces and bone fragments were not given coordinates. The artifacts were found scattered within a sandy and gravel level 80 cm thick, corresponding to layers 12 *n* and 11 *m* i.e. the gravel conglomerate and cross-bedded sands described by [1] (Fig 2; Fig A in S1 File). However incomplete, the record of individual provenience written on artifacts and faunal remains indicates that the great majority of materials come from the cross-bedded sands of 11*m*.

Their state of preservation (Tables 1–3) and the scarcity of unretouched flakes (n = 88) clearly indicate selective removal by water current and short-distance transport movement of the artifacts. Long-distance fluvial transport is limited since a good number of pieces are fresh or only slightly abraded. The presence of three bifaces (TP 23, TP 31 and TP 34; Figs G, I, J in S1 File) with length of 10 to 16 cm showing different states of abrasion (very abraded on one face, fresh or only slightly abraded on the other face) suggests that some large pieces were partly buried in sands and the exposed upper face was abraded in situ by the flow of sandy water. One quite abraded biface of limestone (TP 39; Fig K in S1 File) has some fresh scars at the tip and base. Not surprisingly, resistance to abrasion is, at least in part, function of the toughness of the rock, so that all flint pieces are fresh and most limestone pieces are abraded or slightly abraded. Most faunal remains show traces of rounding [1].

**Table 1:** Counts of biface by raw materials and state of preservation^a^.

State of preservation	Flint	Limestone	Siliceous Limestone	Quartzite	Sandstone	Total
Fresh	5	3	1	1	1	11
Slightly abraded	0	6	2	2	0	10
Abraded	0	5	0	0	0	5
Very abraded	0	1	1	0	0	2
Differential abrasion	0	3	1	0	0	4
Total	5	18	5	3	1	32

^a^ “Flint” includes one biface of coarsely textured, opaque, microcrystalline silica (sometimes called chert). Limestone is fine-grained and includes lithographic and micritic varieties. “Slightly abraded” and “abraded” describe pieces with a moderate or high degree of rounding, some pieces also showing evidence of fluvial polish and striations [42]. The physical state of preservation of 19 pieces could not be observed and are excluded from this table.

**Table 2:** Counts of small tools by state of preservation^a^.

State of preservation	N	%
Fresh	27	49.1
Slightly abraded	23	41.8
Abraded	5	9.1
Total	55	100

^a^One piece is covered by concretions and could not be diagnosed. Eight pieces have differential abrasion, showing fresh retouch on an abraded or slightly abraded blank; five of these have double patina, indicating reuse of an older tool.

**Table 3:** Counts of cores by raw material and state of preservation.

State of preservation	Flint	Siliceous limestone	Limestone	Total	%
Fresh	7	-	1	8	47.1
Slightly abraded	6	1	-	7	41.2
Abraded	2	-	-	2	11.8
Total	15	1	1	17	100

The small tools in our sample are 64. Not included in our analysis are 11 pieces on display in the Pigorini Museum, 5 pieces too abraded for identification and 88 flakes of various raw materials (mainly limestone) with only natural damage. A flake considered by Piperno and Biddittu (fig 52:1, [29]) a possible Levallois flake is in fact a biface shaping flake according to descriptions and definition of shaping flakes [43,44]. It has a very typical V-shape (a consequence of low transverse convexities on the worked surface, as usual on bifaces), a narrow platform with a tiny lip, a distal curvature of the profile, and on the dorsal face a distal negative coming from the opposed edge of the biface. There are 13 small flakes from the retouch of small tools. All retouched pieces are of flint, their state of preservation is indicated in Table 2.

There are 19 cores; two have been subsequently retouched and are treated as small tools.

This is clearly an incomplete assemblage. The scarcity of flakes, compared to the number of bifaces and cores, is indicative of fluvial winnowing. The sedimentary context by itself does not give a clear indication whether the material derives from a single or from multiple occupation episodes. Undoubtedly part of the material has been subjected to some transport and redeposition while differential abrasion and double patina suggest that at least some material was deposited by human activity at the excavated site, possibly in different episodes of occupation. However, as noted by [29], the large number of artifacts and the similarity between fresh and abraded pieces support the idea that this is an aggregate of closely related artifacts representative of the industry to which it relates. Our analysis shows that they share a large number of technical and typological features in recurrent association.

#### A structural model of Acheulian biface variability in Western Europe

According to E. Boëda [45–47] two types of bifacial pieces can be distinguished in the biface industries of the Lower and Middle Paleolithic. The first type, called “biface—tool” (*biface-outil* in French) is characterized by a shaping planned to achieve the making of a specific product, a tool with a bifacial working edge. The design concept is guided by a unique purpose, making a tool whose parts are synergistically associated. The shaping of the tool volume and its cutting edge are done in a continuous and complementary succession of knapping strikes. The interactive structure of these bifaces allows only a limited reworking of the edge; extensive reworking or repair will require total restructuring of the tool volume.

The second type-called”bifacial tool blank” (*biface support d’outil* in French) presents a different design concept. Bifacial flaking is done for the purpose of obtaining a piece of a predetermined shape (similar in this to a Levallois flake) which can receive different tool edges and can be resharpened several times. This is made possible by producing a volume with a hierarchical and asymmetric structure, which is often plano-convex [48].

A recent study by [49] suggests that the bifaces of level *m* at Torre in Pietra correspond to the definition of “biface-tool” and can be compared to other broadly contemporaneous biface assemblages in northern France such as of Soucy 1, Soucy P3 and Cagny-l’Epinette II. The suggestion does not rest on explicit documentation. Thus our study of the layer *m* bifaces was directed to test in a systematic manner the adequacy and consistency of this interpretation and to explore the effect of raw material on design.

The bifaces are documented by detailed outline drawings somewhat different from those proposed by [49]. They illustrate the order of removals using symbolic conventions with scar direction lines, the profile of removals (flat, convex or concave) and the presence or absence of negative bulbs. The presence of negative bulbs is indicated by a dot at the end of the arrow. On these schematic drawings we have taken care to distinguish: a) the negatives of shaping flakes (filled in gray) which the knapper used to build the volume of the bifacial piece. When possible we order the negatives from the oldest to the most recent to follow the chronology of removals on each face and from one face to the other. This allows us to understand how shaping is related to the biface volume. b) the negatives of retouch flakes (filled in yellow, orange or red, from the oldest to the youngest) which have sharpened and finished the biface cutting edges. This graphic representation illustrates the extent and the chronology of retouching.

**Fig 10:**
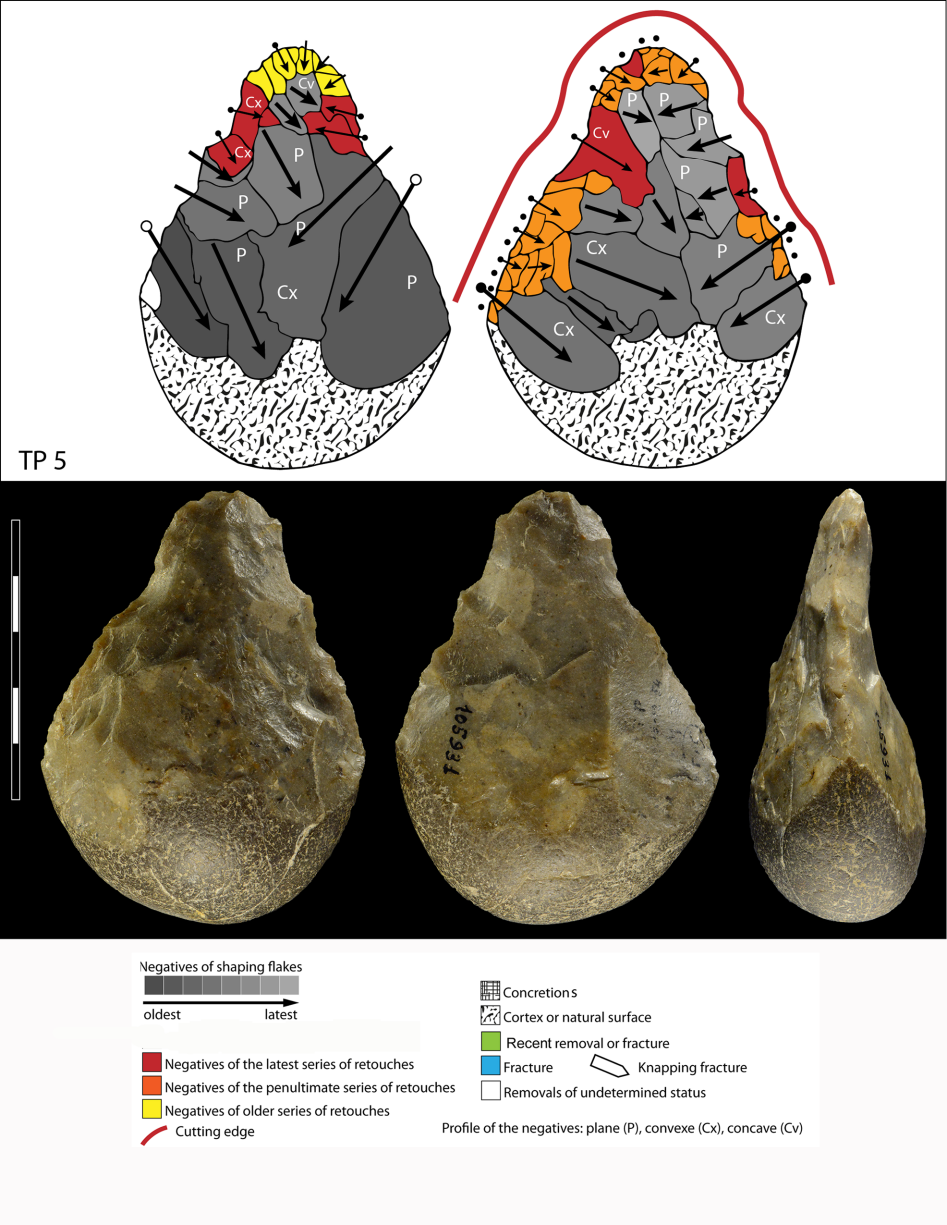
Flint biface TP 5, fresh.

**Fig 11:**
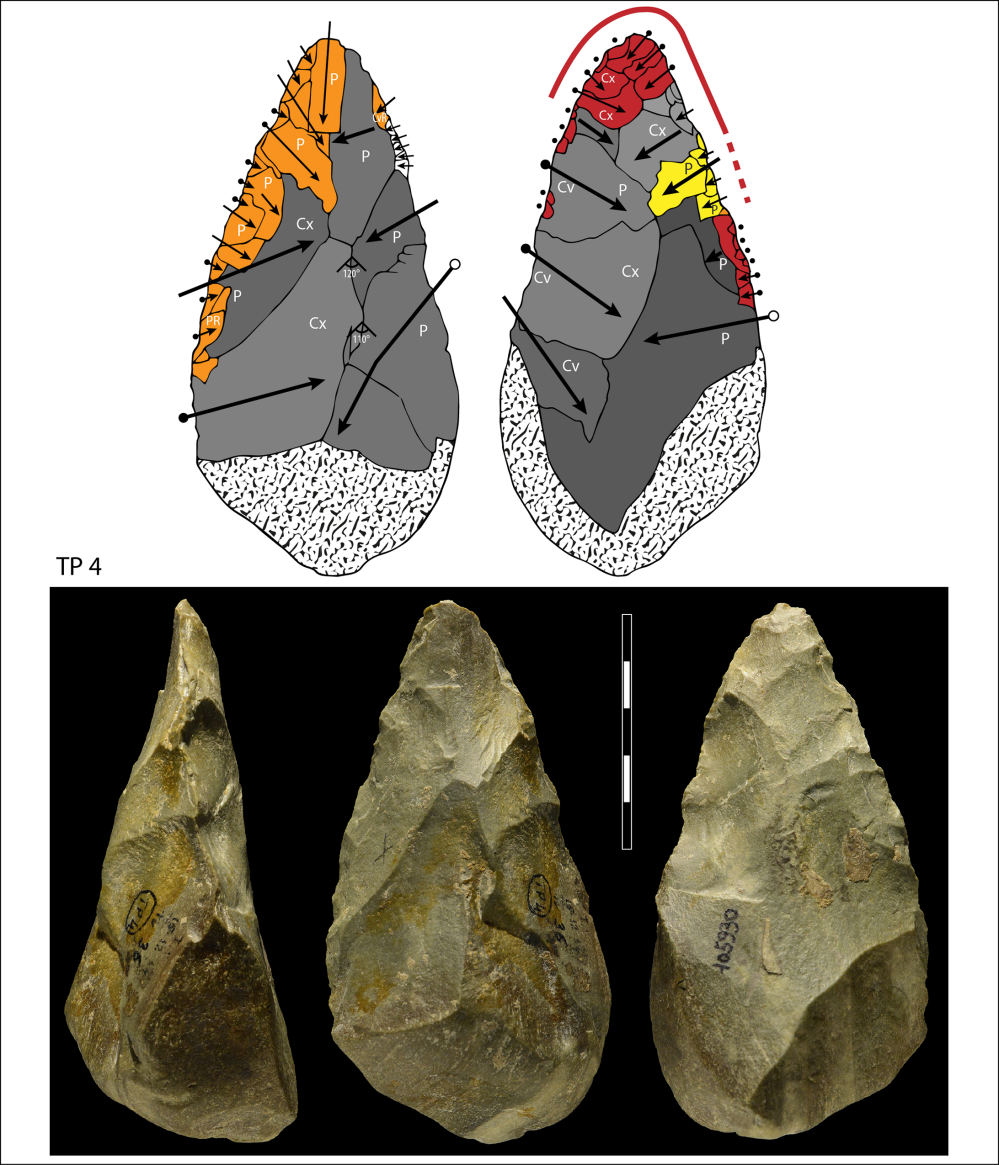
TP4, siliceous limestone. Symbols in drawing as in Fig 10.

**Fig 12:**
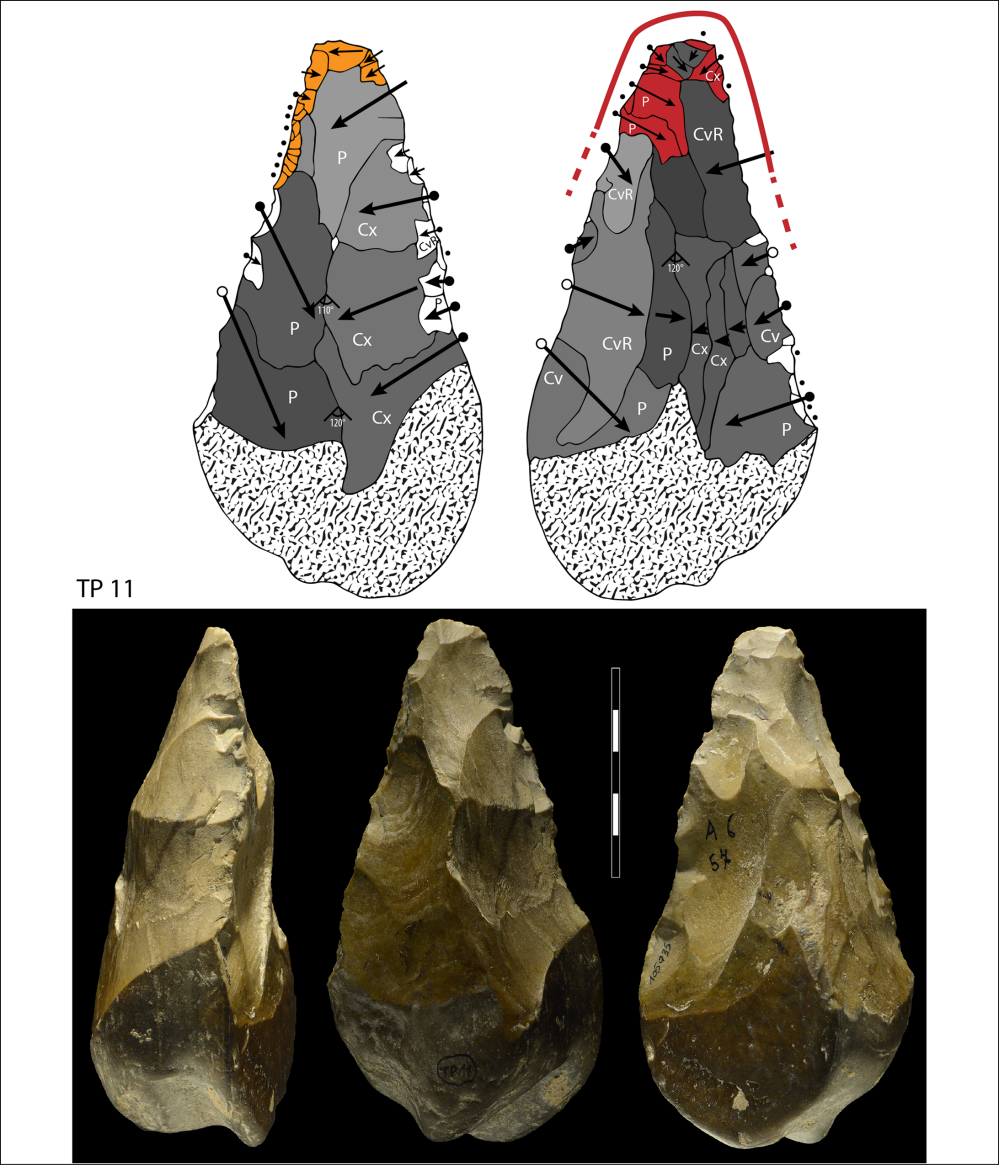
Flint biface TP 11. Symbols in drawing as in Fig 10.

**Fig 13:**
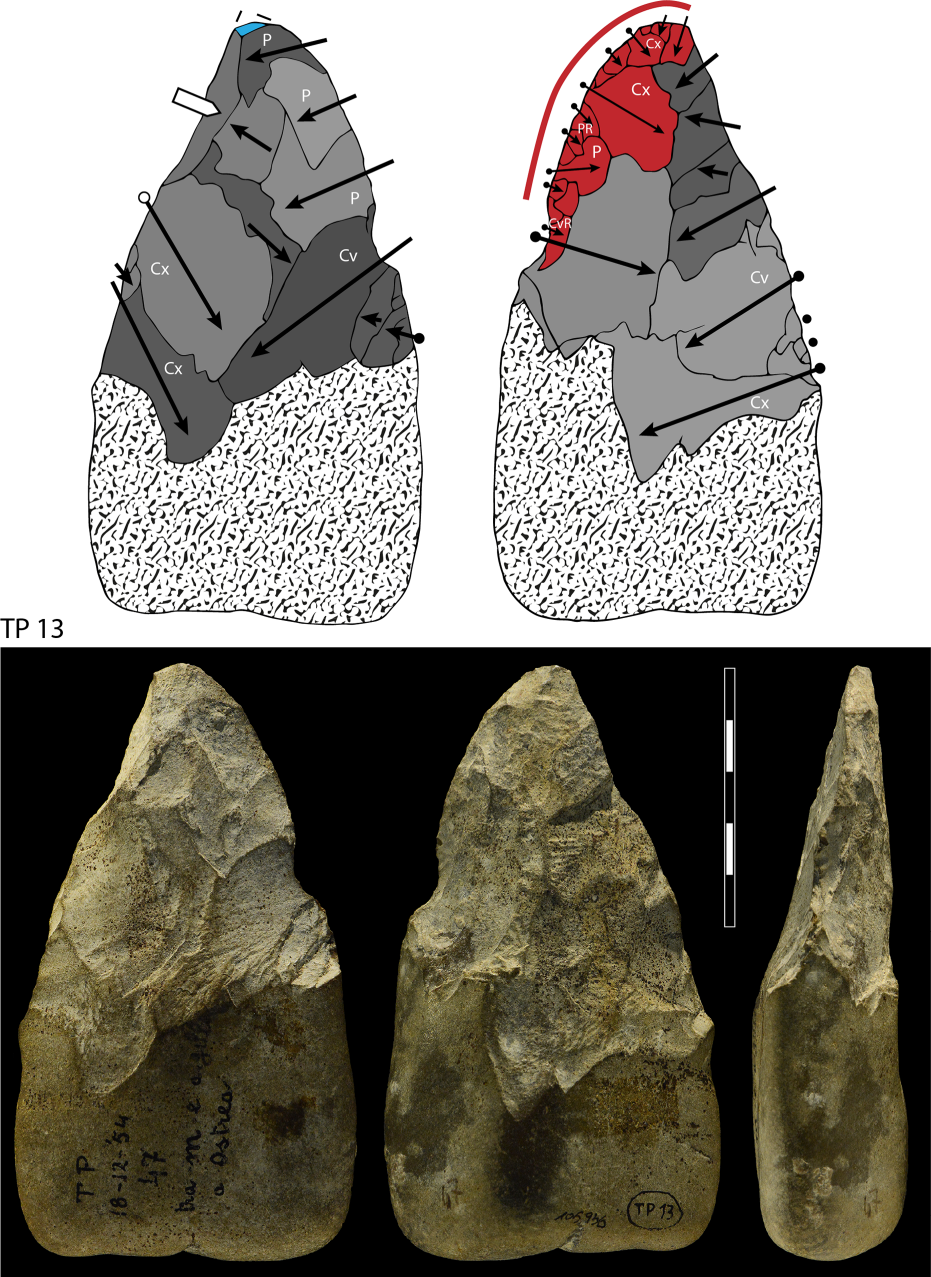
TP 13, limestone. Symbols in drawing as in Fig 10.

**Fig 14:**
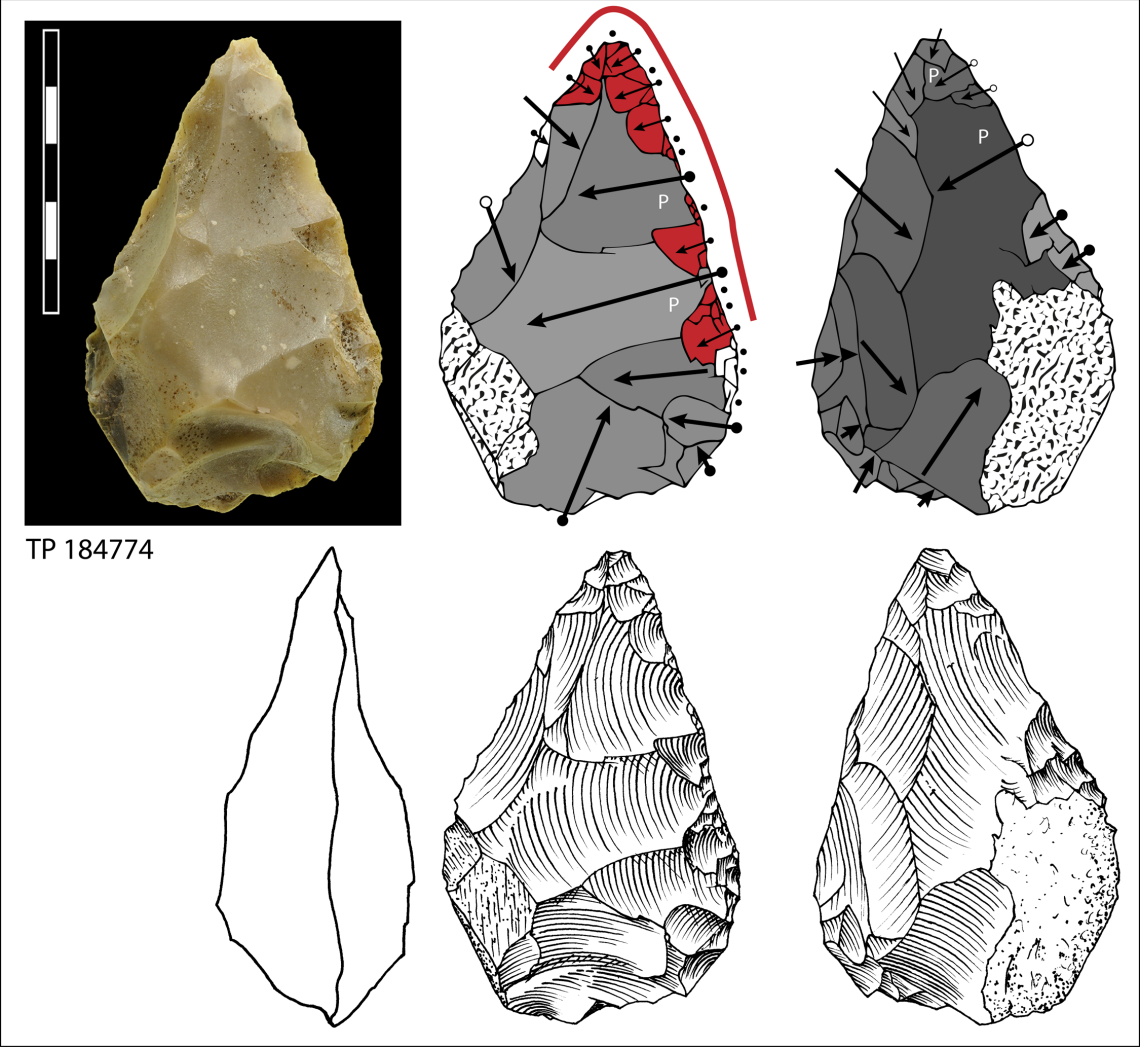
Limestone biface, Pigorini Museum catalog number 184774. Symbols in drawing as in Fig 10.

The quality and precision of our reading depends from the degree of alteration of the biface surfaces. On pieces abraded by fluvial transport it was sometime difficult to observe the shaping chronology since irregular retouch due to clast collision [42] mask or remove the man-made retouch. Schematic drawing and photos of six bifaces are in Figs 9–14. Schematic drawing and photos of other bifaces in our sample are Figs F-R in the S1 File.

When the chronology of shaping removals can be observed, we can see that they are not organized hierarchically (contrast Fig K in S1 File of [50]). Removals on bifaces TP 4,TP 5,TP 11 (Figs 10–12) alternate from one surface to the other or from one edge to the other without a clear organization. Removals on one face or a portion of one face may have the same or varying profiles. The reduction sequences seem rather short if we consider the extent of residual cortical portions, their orientation and the number of large scars.

Two different configurations of volume can be seen. The majority of bifaces are characterized by a relatively symmetrical transverse section because shaping has been done with similar removals from the two edges (e.g. TP4, TP5, TP11,TP13,184769; Figs 10–14; Fig O in S1 File). The maximum thickness is generally in the middle part of faces, at least for distal and mesial portions (e.g. TP 3, Fig N in S1 File). Excluding knapping accidents (TP 13) the retouch scars which form the cutting edges occur at the tip and on the two sides of each biface.

Nevertheless, several bifaces, at least in their distal part, have a clear asymmetric transverse section so that the maximum thickness is displaced toward one edge (184774, 19-8-57, 3-11-55, 12-11- 55, TP 12; Figs 13 and 14; Fig F in S1 File). The retouch scars that form the cutting edges occur on the apex and on the less thick edge. This kind of structure, defined as backed biface, is known in the French Acheulian (fig 17:1, [51]) (fig 7:4, [52] (figs 16.5 and 91.2, [53]) and younger Middle Paleolithic industries [54]. In some cases (like in TP 12; Fig F in S1 File) shaping is limited to the distal part and the rest of the edge is unworked (with cortex or a natural surface). Most likely it is the blank configuration (a narrow blank with a thick edge) which imposes an asymmetrical structure.

In rare cases shaping and finishing scars are not different (TP 42; Fig O in S1 File). In other words, after a few shaping removals that have not really modified the pebble volume, the edges have been formed by deep retouch scars made by internal percussion with a hard hammer. In profile the edges have a sinuous delineation.

In sum, at Torre in Pietra biface shaping does not result in a structure with asymmetrical, hierarchical, plano-convex sections. This type of section allows series of resharpening without requiring a reshaping of the biface volume [55–56, 47, 50]. When several phases of retouch can be observed, their function is to retouch the same cutting edge alternating between faces or between sides. This is the case of TP 4,TP 5,TP 11. There is no hierarchical ranking of scars making the two faces of a cutting edge, as it has been observed in the late Acheulian (e.g. Gouzeaucourt or Barbas level C [56–57] or even on Still Bay bifacial points [50].

Signs of resharpening or reduction are rare or minor. On TP 5 (Fig 10) there are three phases of retouch. The first two series (yellow, orange) complement each other and were probably done in succession. They formed a long cutting edge at the tip and distal sides of the biface. The third series (red) consists of alternating discontinuous retouch scars done by internal percussion and forming notches. This kind of reworking which modifies the outline has been described on bifaces [57–59] and even on Blombos Still Bay points [60] and may be the last resharpening before recycling. The same history can be observed on biface 184769 (Fig P in S1 File) with deep notches which alter the previous edge. This kind of notching can be extensive thus modifying the piece structure. For example on TP 33, a short and thick biface, scars on the right side of face B (covered by concretions but still recognizable) have created deep notches on the other face (Fig S in S1 File). As a consequence of this reworking, which affects only face A, the cutting edge in profile is twisted, as noted by Roe (fig 3:6–7, plate 21[61]) on English bifaces. Aside from this example, instances of important reworking of volume and new sharpening of cutting edges are extremely rare. This might be the case of TP 4 1957 (Fig R in S1 File) where the last shaping removals have clear negative bulbs. This is unusual and could be a sign of a restructuring of the biface volume. The effect of raw material on shaping shows on bifaces with a clear asymmetric transverse section. This organisation of bifacial shaping, continuous on one edge but restricted to the distal portion of the opposite edge, might have been unavoidable to perform successful shaping of biface on thick and especially narrow cobbles. Our technological analysis shows that most of the Torre in Pietra bifaces were shaped using procedures planned to obtain a specific purpose, a tool conceived as a whole and whose structure did not permit multiple resharpening, as it can be observed in the classic Acheulian of Western Europe [49]. They fall into the category of “bifacial tools” described by [47] and observed by [49] in broadly contemporaneous Acheulian industries, like Soucy in the Paris Basin [62].

**Fig 15:**
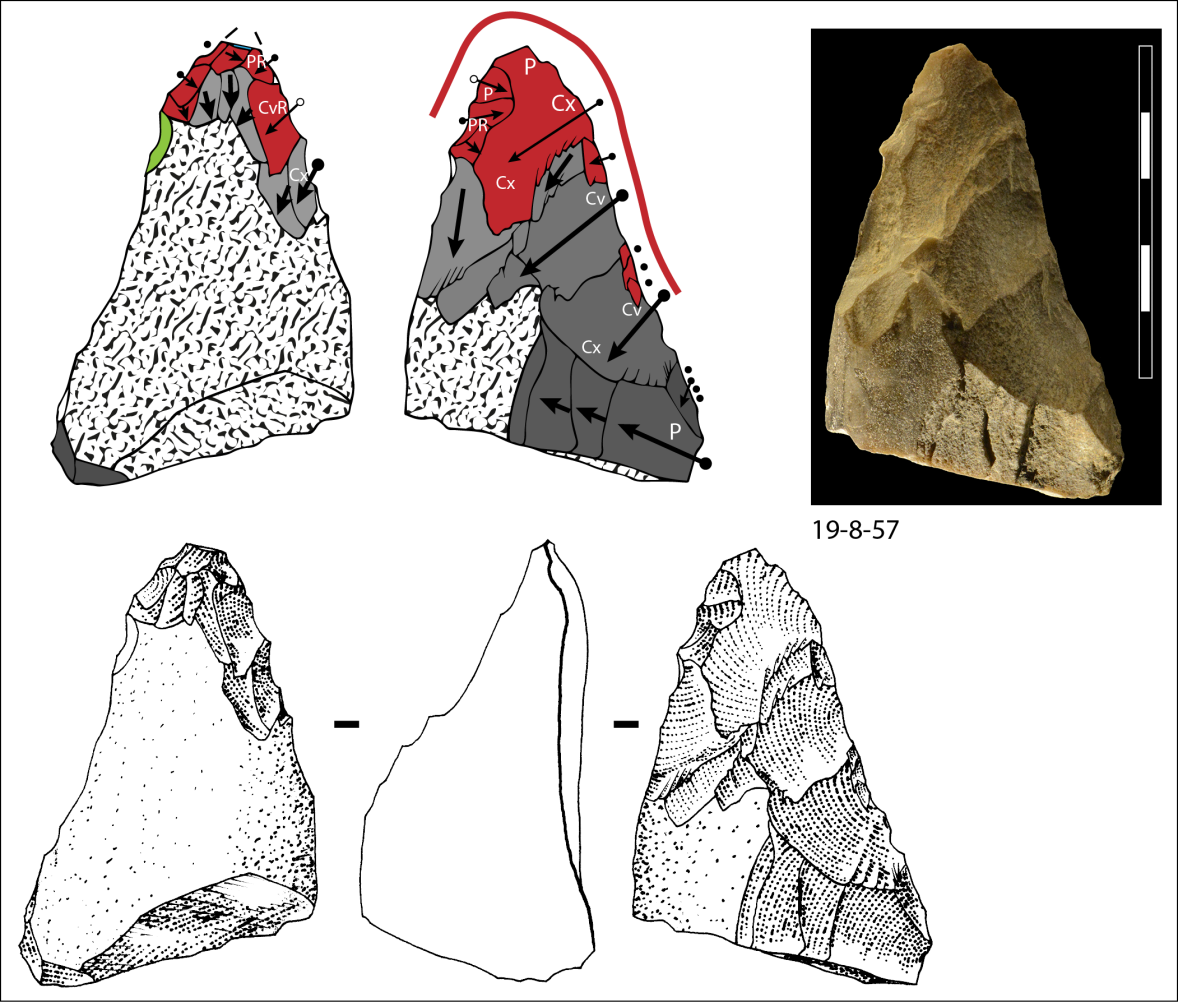
Bifacial piece of quartzite, number 19-8-57. Symbols in drawing as in Fig 10.

Finally we note that the preferred biface shape is pointed. The majority of bifaces have a fully (n = 24) or partly (n = 3) cortical base, a cortical back, that is, a proximal side with some cortex (n = 3; Fig 14) or a base with a thick and flat fracture surface (n = 2; Fig 15). Only 13 (of 45) have a base entirely worked around the edge. It is clear that the base was controlled by prehension. The knappers selected as blanks cobbles of an oval shape which offered an easy prehension and allowed limited shaping. In other words, it was the volume more than the kind of raw material that influenced the choice of knappers.

Discussion. Understanding variability in form, size, shape, types and design of bifaces has often been eluding us. Boëda provided us with a clear and elegant model that allow us to frame and empirically ground explanations. The two types he describes are not completely mutually exclusive, as the Torre in Pietra analysis suggests; the same co-existence is documented by the bifaces of layer 2 at the Petit-Bost site (SW France) dated to the end of MIS 9 [63]. Like all models, Boëda’s model is a simplified representation of the real world but it has predictive value serving as a guide to analytical observations and making complex questions more tractable.

The second Boëda’s type is well-represented in French assemblages with bifaces dated to MIS 7–6 (e.g. Pech de l’Azé II, Combe Grenal) and in the later Mousterian of Acheulian Tradition, dated between 70 and 40 ka (figs 5–11, [64]) (figs 41, 43, 44, [65]) [59,66–68].

In contrast to French sites, from the end of MIS 9 onward, biface industries disappear in the Latium region. The first evidence of non-biface industries in Latium is from the sites of La Polledrara, now dated to about 325 ± 2 ka, [24, 69], Sedia del Diavolo and Monte delle Gioie in the Aniene valley, dated to MIS 8.5 (Fig 9). At Torre in Pietra level *d* is an early Middle Paleolithic dated to MIS 7, without bifaces and with Levallois debitage. However, elements of continuity with the older level *m* are present in the configuration of small tools (see Conclusions).

#### Cores of level *m*

**Fig 16:**
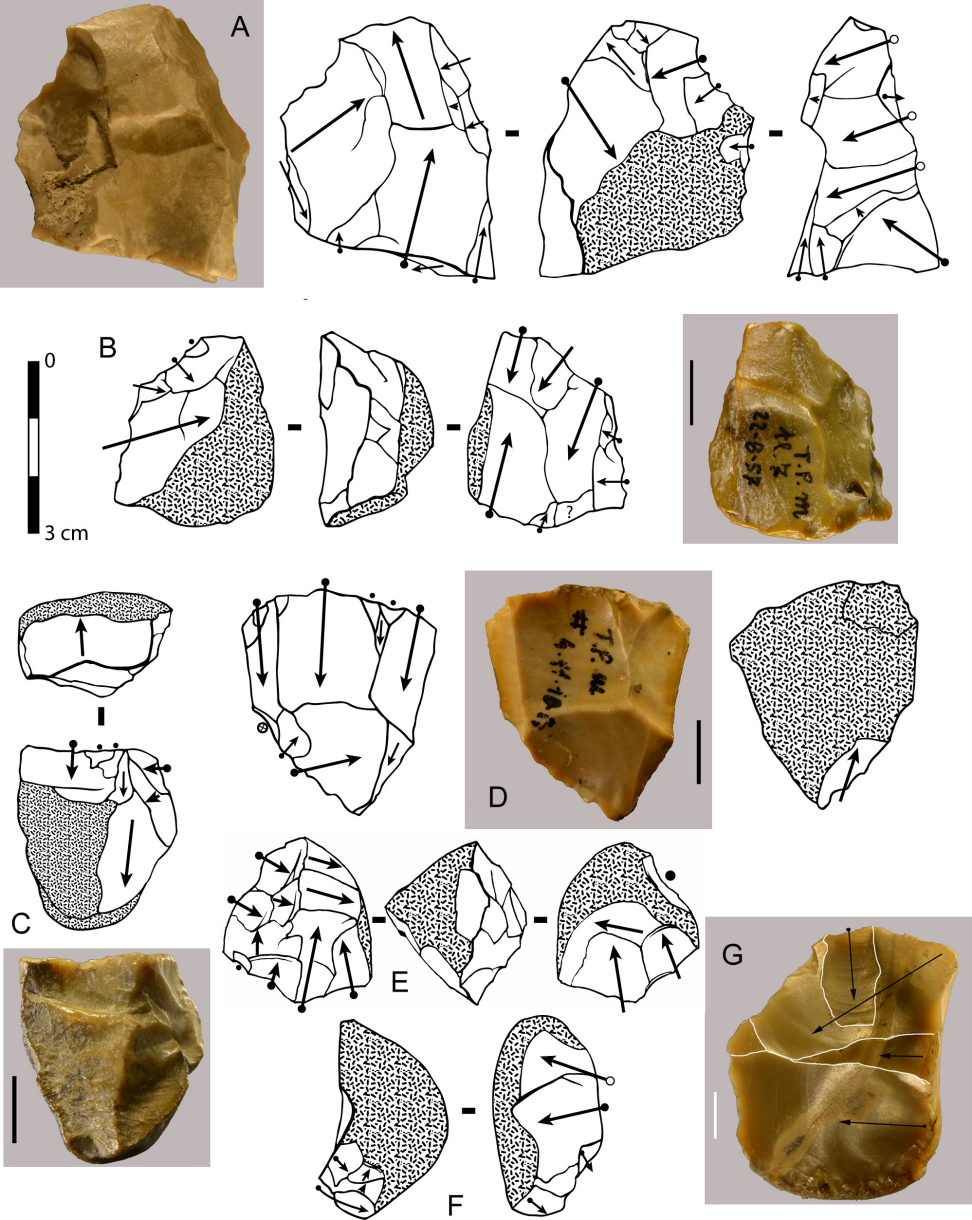
Torre in Pietra, level *m* cores, all flint. (A) no. 8, multidirectional, three debitage surfaces, fresh. (B) no. 20, centripetal but not Levallois, abraded. (C) no. 16 unidirectional one debitage surface, abraded. (D) no. 14, bidirectional, one debitage surface, slightly abraded. (E) no. 11, two debitage surfaces, abraded. (F) no. 4, one debitage surface, fresh. (G) no. 3, broken, one debitage surface, bidirectional, fresh. Scale bar in photos = 1 cm.

Fifteen of the 17 cores are of flint. One of siliceous limestone is a fragment with only one scar; another is a cobble of limestone with one scar only. The flint cores can have one or more debitage surfaces with a certain prevalence of unidirectional removals but there is no repeating pattern in terms of size and shape and the Levallois technique is completely absent (Fig 16). Most blanks are relatively small pebbles, three others are on blocks with a alluvial cortex. The lack of repeating features is also evident in the flint flakes, the flake shape is variable and irregular.

#### Small tools of level *m*

**Fig 17:**
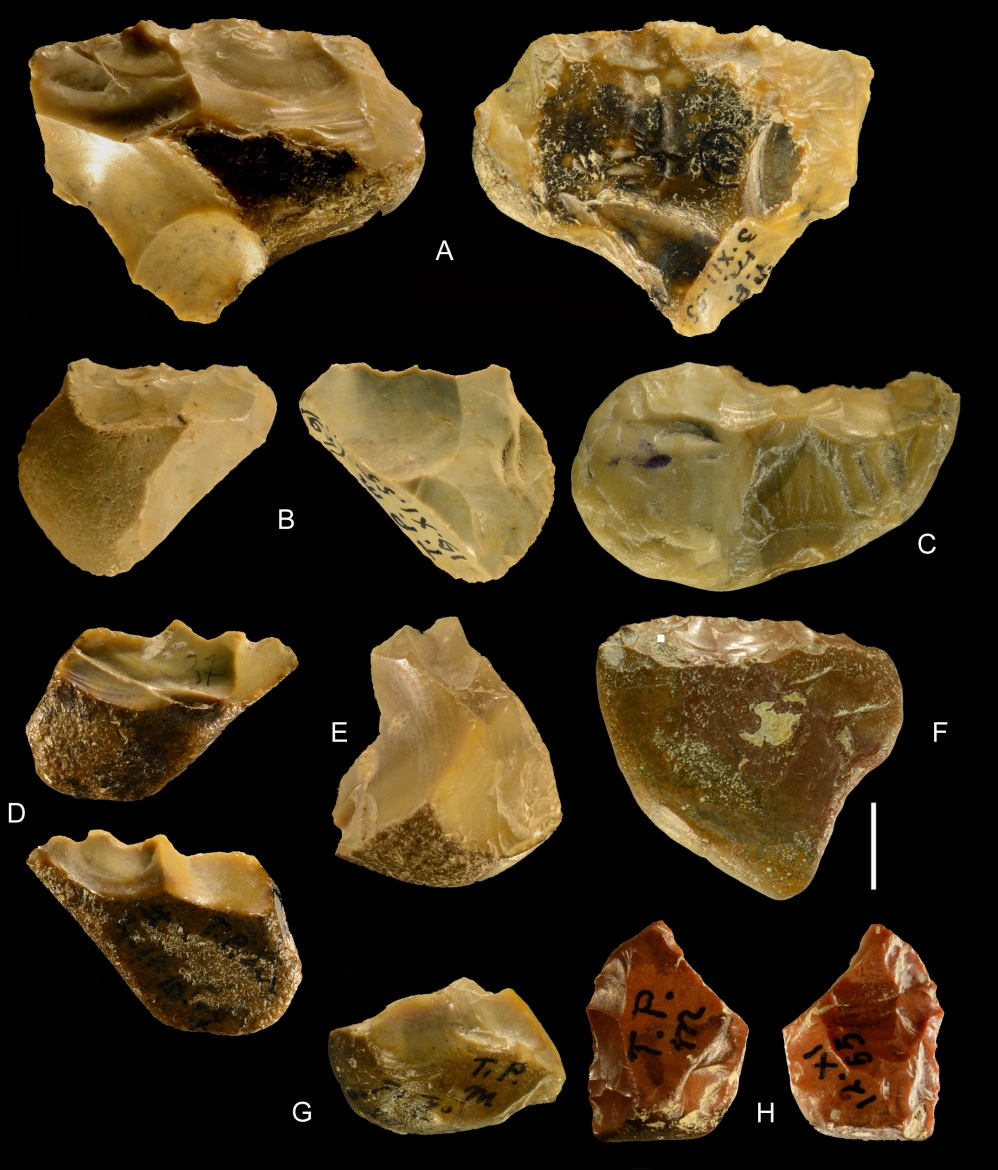
Torre in Pietra, level *m*. Small tools on pebbles with bifacial (A, D, TG, H) and unifacial retouch (C, F); small tools on core or core fragment (B, E). Catalog numbers: 32, 47, 34, 2, 49, 52, 3, 14. Scale 1 cm.

**Fig 18:**
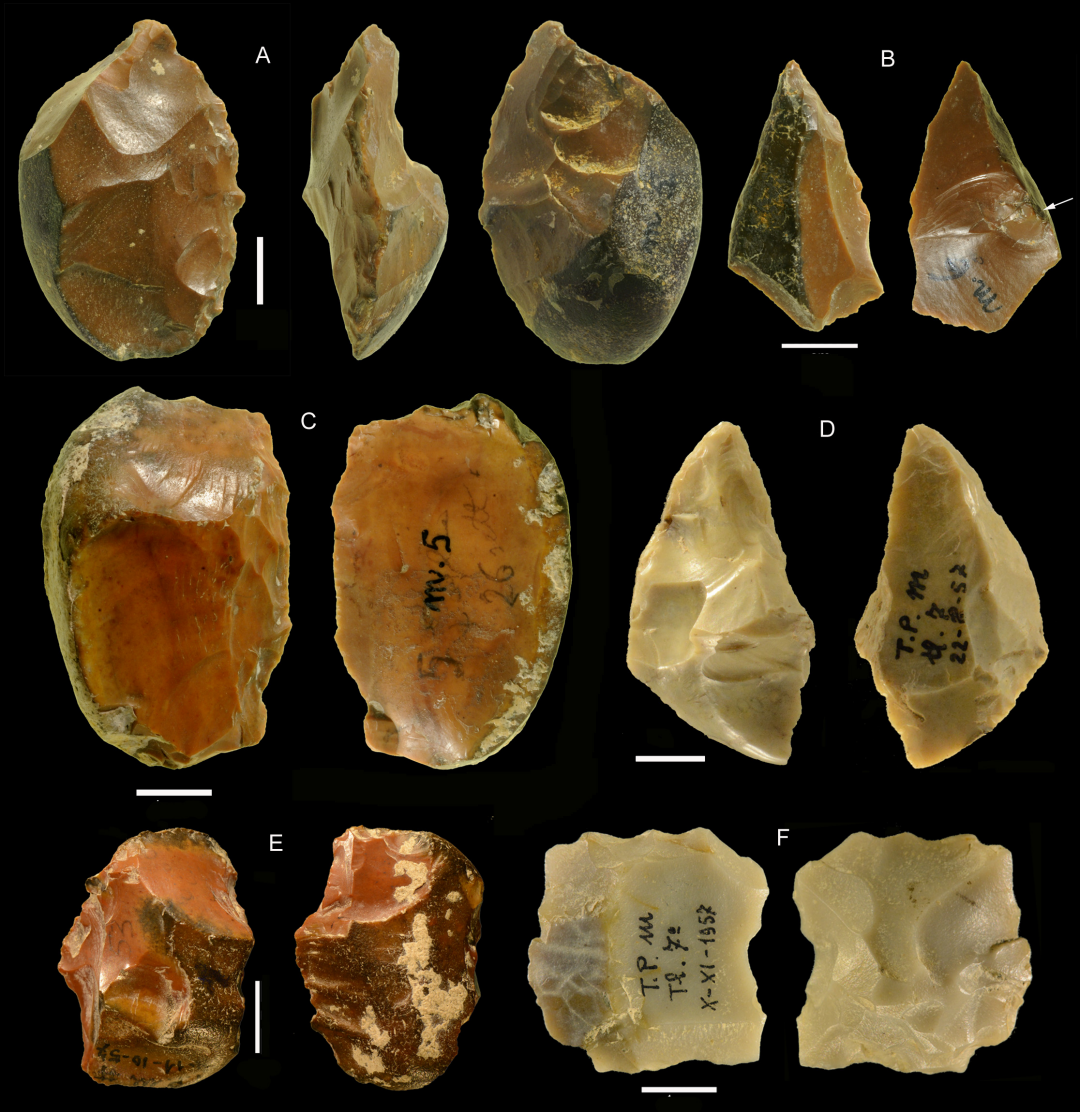
Torre in Pietra, level *m*. (A) Small bifacial piece with a cortical back and a denticulate edge; (B) Déjeté scraper on flake; (C) side scraper on a flake with cortical back; (D) Broken bifacial point recycled with scraper retouch; (E) denticulate on pebble; (F) denticulate on core. Catalog numbers: S.N.1, S.N. 5, S.N. 3, 22-8-57, 44, 17. Scale 1 cm.

All small tools are of flint. Tools on flakes are about 63% of the total but a significant number of small tools are on pebbles or on core (Table 4; Fig 17; Fig 18E and 18F). Biface shaping flakes (Fig T in S1 File) were not sought after as blanks for retouched pieces. Initial shaping flakes are difficult to separate from initial debitage flakes but the raw material indicates that the use of shaping flakes was minimal, if at all. First, all small tools are of flint and 15 of 17 cores are also of flint while only 4 bifaces (4/50) are of flint (two others are of coarse flint). Second, two thirds of the flakes used as blanks are cortical or partly cortical so they cannot be considered as advanced or final shaping flakes.

**Table 4:** Blanks of small tools^a^.

Categories	N	%
Flakes (31) and flake fragments (6)	37	60.6
Pebbles	13	21.3
Core, core fragment, chunk	9	14.8
Recycled tool fragment	2	3.3
Total	61	100

^**a**^(indeterminate blanks are excluded).

The preference for thick blanks is underlined by the fact that 41% of the flake blanks (12 of 29) have a thickness/length ratio ≥ 0.4, twice as much as in the younger level *d* where only 18.8% (18 of 95) have a thickness/length ratio of flake blanks ≥ 0.4.

Whether on flake or on pebbles and cores, the most common edge types are side scrapers (Figs 17F, 18B, 18C and 18D) and denticulates (Fig 17B and 17D; Fig 18A, 18E and 18F); irregular edges are common.

**Fig 19:**
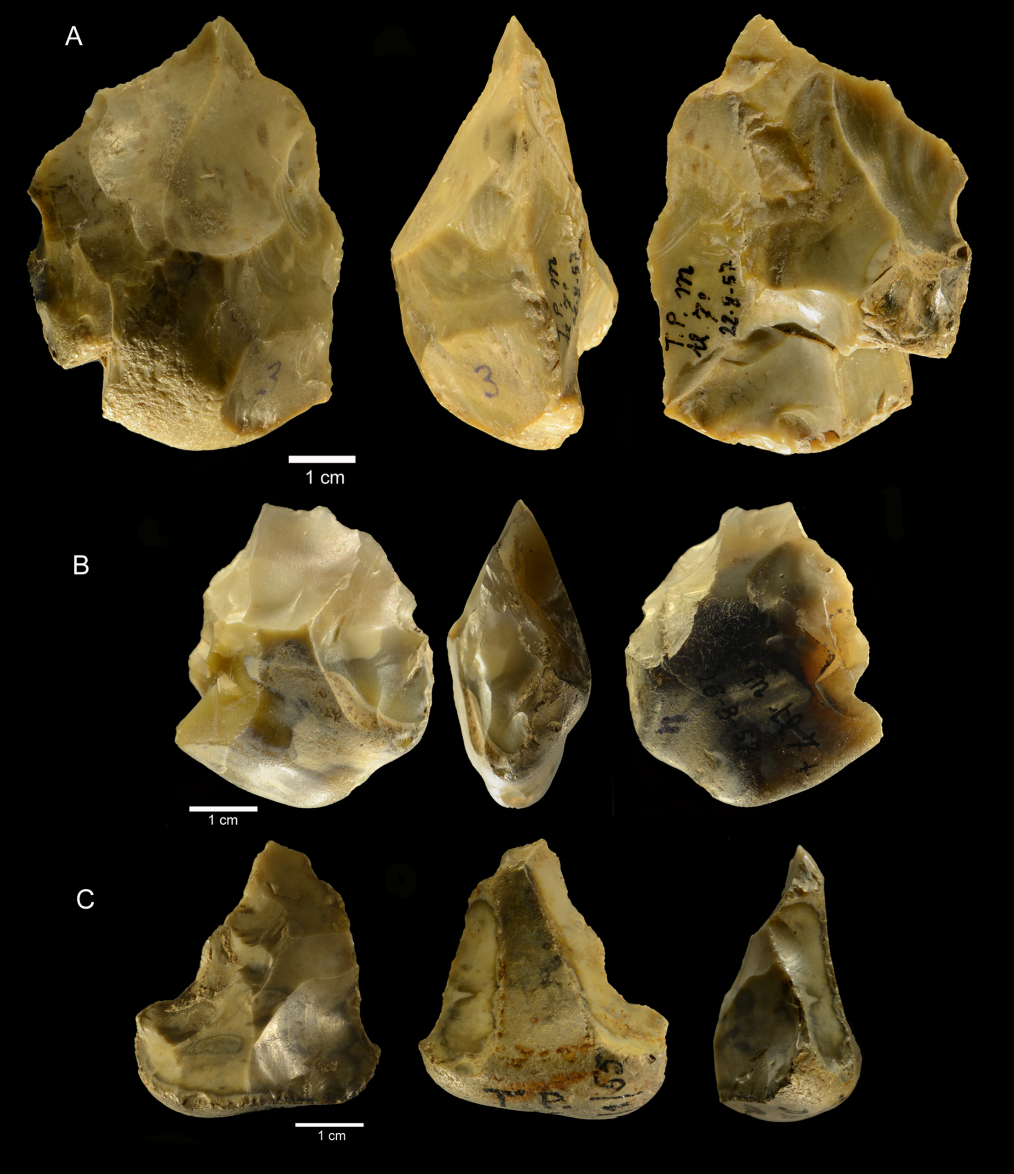
Torre in Pietra, level *m*. **Small bifacial pieces with a scraper or denticulate edge.** Catalog numbers; 53, 54, 55. Scale 1 cm.

Bifacial shaping and retouch is relatively common, not surprising in an assemblage dominated by bifaces. Some flint pebbles have been bifacially shaped but they cannot be regarded as cores (Fig 19). The organisation of removals is oriented toward the shaping of cutting edges composed of notches. The use of adjacent notches to create a pointed bifacial tool (Fig 19A) was already observed in the Acheulian from Gouzeaucourt G [57]. More generally a few small bifacial tools could be encountered in Acheulian industries as at Cagny-l’Epinette (fig 10:2, [70]). However they cannot be considered as true bifaces due to their morphology and their short chaîne opératoire.

#### Concluding remarks on level *m*

The mode of production of small tools based on the use of small flint cobbles can be observed again in the assemblages of Castel di Guido, Sedia del Diavolo, Monte delle Gioie and Torre in Pietra level *d*. The latter three assemblages span the transition from Lower to Middle Paleolithic; they are variable and different enough that they should not be lumped into the same industry. But the similarities in certain modes of production suggest a common body of technological knowledge, thus a degree of transmission of technical habits, specifically a common preference for thick blanks. In this paper we limit our comparisons to the Castel di Guido and Torre in Pietra level *d* small tools. The bifaces of Castel di Guido will be treated in a separate paper.

### Castel di Guido

Castel di Guido is an Acheulian site about 20 km WNW of Rome (Fig 1), excavated between 1980 and 1991 on an area of 1100 sq. m. [71]. U/Th and ESR dates indicate an age between 327 and 260 ka [72]. The deposits are considered part of the Aurelia Formation. The faunal and lithic remains were distributed on a single surface but geological and taphonomic studies suggest that the site is a palimpsest of several episodes of human use with partial reworking, some removal of the fine fraction and association on the same surface of remains originally belonging to separate phases of use [71, 27, 28].

As at Torre in Pietra double patina occurs on some small flint tools (5 of 64 at Torre in Pietra, 14 of 94 at Castel di Guido) indicating reuse of a desired raw material. The site is well known for its bifaces and other tools on elephant bone diaphyses [73–74]. The bifaces, partial bifaces and unifaces are done on cobbles of various raw materials, mainly chert, limestone and siltstone.

### The small tools of Castel di Guido

Sorting and sampling. We have excluded from analysis flake fragments, chunks, natural pieces and the very few unretouched flakes (n = 15). After checking a database by Giovanni Boschian and the monograph catalog [71] we also excluded from our database a fairly large number of pieces (88 pieces) for one or more of the following reasons: pieces too rolled, too altered or too damaged for correct identification, various pieces from the site of Malagrotta that had been mixed with Castel di Guido by visiting researchers and pieces without catalog number.

Taphonomy. The small tools of Castel di Guido (n = 94) are all of flint or other form of microcrystalline silica, namely what many archaeologist would call “chert” distinguishing flint as characterized by a fine, homogeneous, glossy texture with smooth fracture surfaces and chert as being coarser-textured and opaque. Their state of preservation is indicated in Table 5. Chert pebbles and cobbles were used for tools of small and large size, including choppers and chopping tools and their length distribution is continuous from 3 to 13 cm. In contrast all Castel di Guido flake tools are smaller than 6 cm; this is also the true in the Torre in Pietra level *m*. Therefore we place in the small tool category retouched pieces on pebble smaller than 6 cm (Table 6).

**Table 5:** Counts of small tools by state of preservation^a^.

State of preservation	Flint	Chert	Total	%
Fresh	43	9	52	62.7
Slightly abraded	21	6	27	32.5
Abraded	2	2	4	4.8
Total	66	17	83	100

^a^Indeterminate cases are excluded.

**Table 6:** Blanks of small tools^a^.

Categories	N	%
Flakes (34) and flake fragments (5)	39	47.0
Pebbles (3 are rolled blocks)	25	30.1
Core, core fragment, chunk	19	22.9
Total	83	100

^a^indeterminate blanks are excluded).

**Fig 20:**
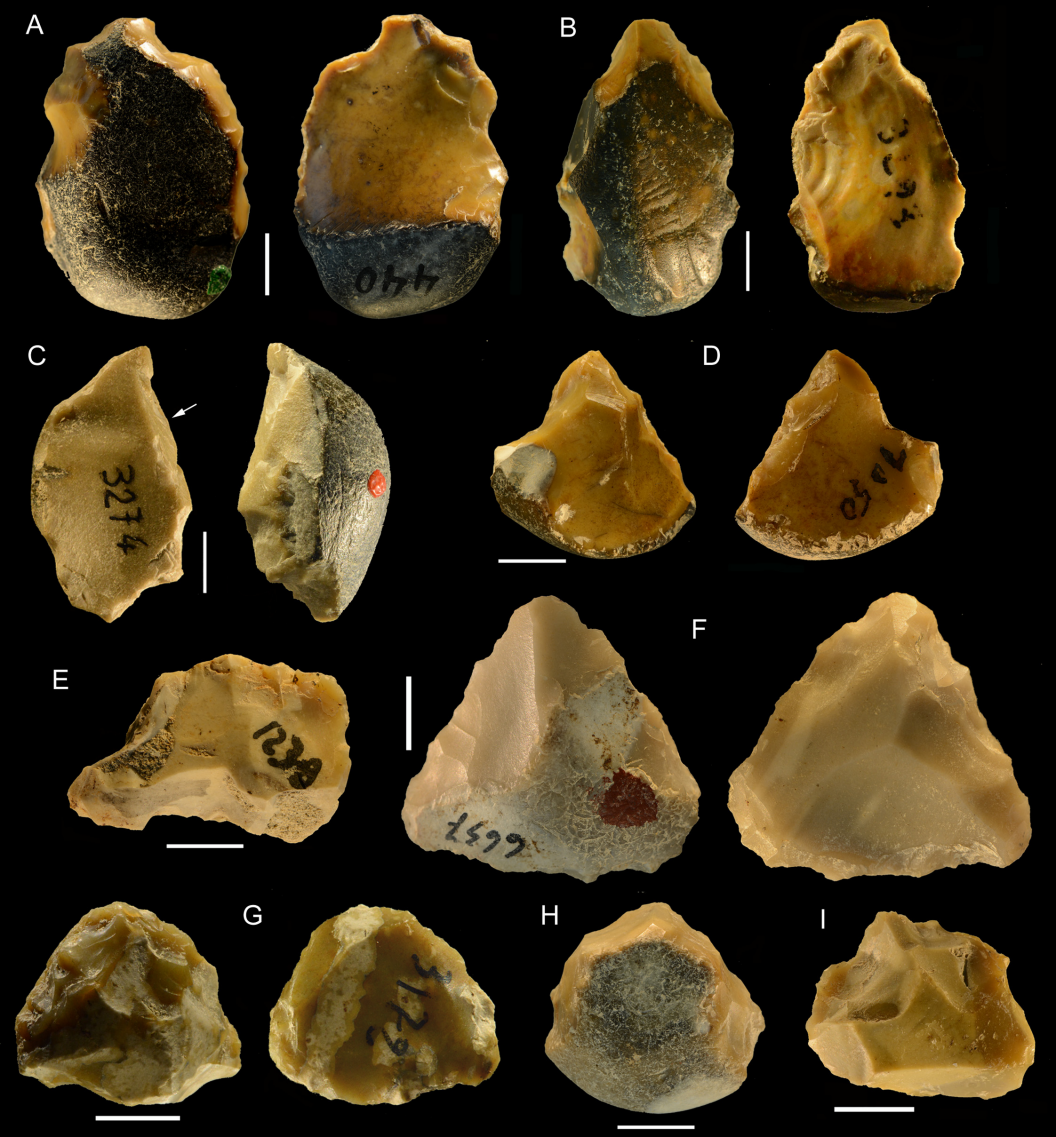
**Castel di Guido flint small tools on negative blanks (A,B,C), on flake (D,F, H) and on core (G, I).** (A) denticulate scraper; (B) convergent denticulate; (C) negative blank with some retouch, the arrow indicates a break prior to retouch; (D) bec on a thick flake; (E) retouched notch on core, note the double patina; (F) convergent denticulate on the ventral face of a thick flake; (G) end-scraper on core; (H) end-scraper on a thick flake; (I) denticulate on a rolled block; a side scraper on the other face was modified by the denticulate. Catalog numbers 440, 3293, 3274, 1050, 1238, 6637, 3172, 3564, 2136. Scale 1 cm.

Split pebbles and the bipolar technique. A few small tools at Castel di Guido are on a particular kind of “core”. Direct hard hammer percussion was used to split a pebble in half, with the resulting “flake” as large as the “core” which has only one large scar. We call the flake “positive blank” and the core “negative blank”. The large surface of the removal which shows the negative of a bulbar scar was used as a striking platform for retouch (Fig 20A–20C). In other cases a pebble is not split in half but has a single smaller removal forming the striking platform for retouch. Negative and positive blanks are also present at Torre in Pietra, in level *m*, in level *d* dated to MIS 7, in the older assemblages of Sedia del Diavolo and Monte delle Gioie, dated to MIS 8.5 as the Via Mascagni succession (Fig 9) [18] and even at Grotta dei Moscerini (Fig W in S1 File).

A variant of the procedure for splitting the pebble in two blanks consists in striking the pebble resting on a soft anvil (e.g. wood or soft limestone) or simply resting the pebble on the ground and maintaining in a vertical position [75]. Flakes produced with this technique (often confused with the bipolar technique) are characterized by a non-conchoidal fracture, a flat ventral face, a concave or crushed bulb of percussion but no measurable platform nor an opposing bulb or shattering of the distal end (which is typical of the bipolar technique). We call them “flat” flakes, using a terminology first used at the Acheulian site of Terra Amata [65].

This variant is particularly adapted to the use of small oval pebbles, to initiate a flaking sequence or to obtain a flake with a sharp edge and no distal damage. The ventral face of these flat flakes and the flat scars of “cores” offered a conveniently uniform knapping surface. This technique is typical of the later Upper Pleistocene assemblages of the Latium region (called Pontinian; [76–78]) but a few cases are also present in older assemblages, especially at Torre in Pietra level *d*.

**Fig 21:**
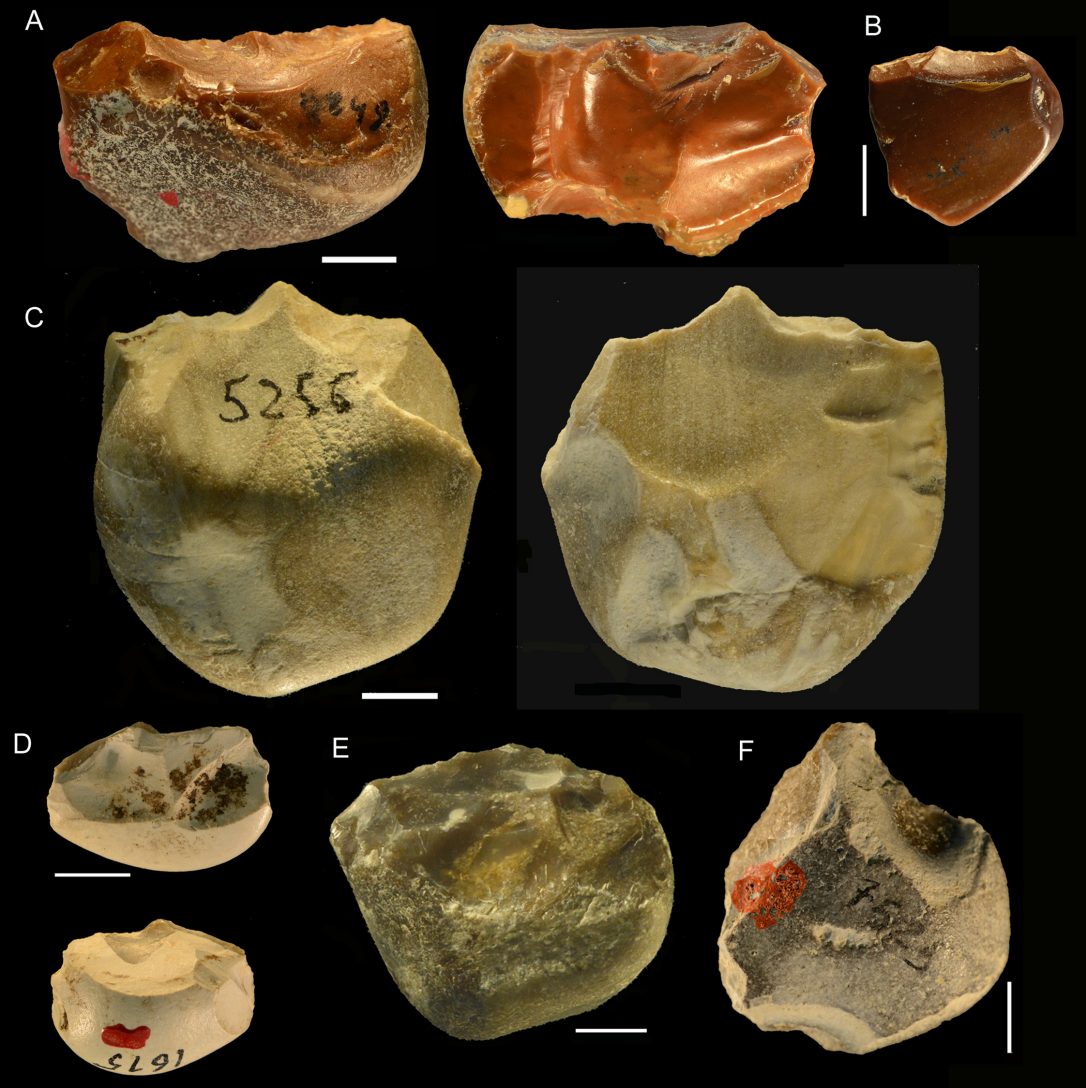
**Castel di Guido small tools on cores and pebbles, A, B, D, E of flint, C and F of chert.** (A) scraper on core, (B) pebble with unifacial retouch, (C) bec on pebble, two large removals form the striking platform for retouch, (D, E) bifacially flaked pebbles with secondary retouch; (F) denticulate on pebble. Catalogue number: 4399, 4398, 5256, 1615, 1192, 7525. Scale 1 cm.

The number of small tools on pebble or core blanks is even higher than at Torre in Pietra level *m*. 60% of the flake blanks (21 of 35) are quite thick, with a thickness/length ratio ≥ 0.4. In several cases it seems that pieces were used as cores and then retouched with scraper or denticulate retouch. This may be the case of Fig 21A, 21D and 21E. The same is in level *m* of Torre in Pietra (Fig 18E and 18F).

Like at Torre in Pietra *m*, the size and shape of cores is quite variable (Fig U in S1 File; Table 7). They can have one or more debitage surfaces with a certain prevalence of unidirectional removals but there is no repeating pattern. As at Torre in Pietra the Levallois technique is completely absent; cores are unpatterned, the flake removal is opportunistic. The lack of repeating features is also evident in the flakes, the flake shape is variable and irregular. The fact that more than 80% of the flakes (retouched and unretouched) exhibit some cortex indicates that reduction sequences are short. Since all the initial blanks of cores and retouched pieces are pebbles, we have classified as cores flaked pebbles that do not carry secondary retouch or almost no retouch (e.g. Fig U: D-G in S1 File).

**Table 7:** Counts of cores by state of preservation and raw material^a^.

State of preservation	Flint	Chert	Quartzite	Total	%
Fresh	14	11	0	25	51.0
Slightly abraded	19	4	1	24	49.0
Total	33	15	1	49	100

^a^Indeterminate cases are excluded.

#### Comparison of Torre in Pietra level *m* and Castel di Guido

The depositional context of Torre in Pietra level *m* and Castel di Guido are different. Both are fluvially disturbed but erosion, removal and weathering, more than accumulation and redeposition, have been the main component of context at Castel di Guido. The behavioral context is also different. There are much fewer bifaces at Castel di Guido, the core tool assemblage is dominated by choppers, unifaces and partial bifaces, and some of the raw material used were siltstone and leucitic lava, which tend to weather to an extreme degree. Bone tools are absent at Torre in Pietra.

The hominins at Torre in Pietra and Castel di Guido had a clear understanding of the specific qualities of flint which came in the form of rather small pebbles and was deemed desirable for the making of small tools. The dates proposed for Castel di Guido are as yet too imprecise compared to the dates now known for Torre in Pietra so that the two assemblages can only be considered as “broadly” contemporaneous. But the almost exclusive preference for flint in the presence of other raw materials, and the preference for thick blanks including negative blanks imply a technology that was shared knowledge and formed part of the transmitted technological repertory of Acheulian craftsmen in the region.

### The early Middle Paleolithic of Torre in Pietra

#### Introduction

In 1978 Piperno and Biddittu [29] published a typological analysis of level *m* and *d* supported by numerous drawings and detailed description of all the artifacts based on Bordes’ method. At the time the assemblage was thought to be of Last Interglacial age. The level *d* assemblage was defined as a Pre-Mousterian industry, a definition that was current at the time for all typologically Mousterian industries older than the Last Glaciation [79–80]. According to [29] the assemblage was characterized by the presence of typical Levallois flakes and a high index of platform faceting. Cores were mostly described as discoidal. The authors also noted a significant component of small tools on flint pebbles (“light-duty choppers”), a feature already seen in level *m*.

According to the technological analysis of Grimaldi [81] the Levallois debitage of level *d* was predominantly of the recurrent unidirectional method with a minor component of products by the recurrent centripetal variant. The unidirectional method without preparation of distal and lateral convexities was, according to him, an adaptation to the morphology of the local raw material, a way of preserving the maximum possible size of products in relation to the small size of flint pebbles.

Our study of the level *d* artifacts is thus directed to an evaluation of the importance of the Levallois debitage, of the effect of raw material on design, of the occurrence of small tools on pebbles and of the possible evidence for cultural continuity in the presence of an innovation like the use of the Levallois method.

#### Taphonomy and assemblage composition of level *d*

The deposits of the Vitinia formation excavated in 1954–1955 (Fig 3; Figs C, D in S1 File) show an alternation of fluviatile sediments of varying granulometry. They consist of sands (essentially pyroclastic in origin) gravels and blocks, the latter deriving from the erosion of the massive travertine of Torre del Pagliaccetto [41] not visible in the site section. The industry and the faunal remains of level *d* excavated on a surface of 40 sq m were scattered in the sandy gravels. There are no records of spatial and vertical distribution of the artifacts but they are said to come mainly from the base of the deposits. During the 2012 fieldwork, some lithic artifacts were found in layers IIIf and IIe of the frontal section (Fig 3) but also in the lateral section. A Mousterian point was found (Fig X in S1 File) at the base of a layer filling a channel incised down to layer IIIe and probably equivalent to layers IIf/IIe.

Table 8 shows the classification of the level *d* assemblage into major artifact categories. Chunks, flake < 1.5 cm and flake fragments (flakes without the platform) are listed but are not included in the analysis. Three pieces in the Pigorini Museum catalog were not available for study. All artifact are of flint, except for two retouched pieces on flakes of coarse flint and one of jasper.

**Table 8:** Torre in Pietra level *d*. Assemblage composition.

Categories	N	%
Cores and core fragments (including 35 Levallois cores)	96	17
Small tools (including 38 retouched Levallois flakes)	214	38
Unretouched flakes, complete or broken but preserving the platform (including 55 unretouched Levallois flakes)	254	45
Total	564	
Percussor	1	
Chunks, flakes < 15 mm, flake fragments	183	

Differences in kinds of raw materials, in their hardness and resistance to weathering and in representation of unretouched flakes prevent a general comparison of the state of preservation of artifacts at Torre in Pietra and Castel di Guido. It is only possible to do such a comparison for the small tools and cores since they are all done on flint or chert. If we compare Tables 2 and 3 (level *m*) with Table 9 (level *d*) for cores and small tools we can see that in both assemblages fresh artifacts are common, with frequencies between 46 and 50%. Castel di Guido has even slightly higher frequencies of fresh flint artifacts, although it is also the assemblage most affected by the selective removal of small pieces by water.

**Table 9:** Counts of major categories by state of preservation^a^.

State of preservation	Cores	%	Small tools	%	Unretouched flakes	%	Total	% of total
Fresh	37	50.0	97	46.0	122	48.4	247	48.9
Slightly abraded	25	33.8	58	27.5	90	35.7	150	29.7
Abraded	12	16.2	56	26.5	40	15.9	108	21.4
Total	74	--	211	--	252	--	505	100

^a^Core fragments and indeterminate pieces excluded. A few very abraded pieces are included in the “Abraded” state of preservation.

#### Formal tools of level *d*

**Fig 22:**
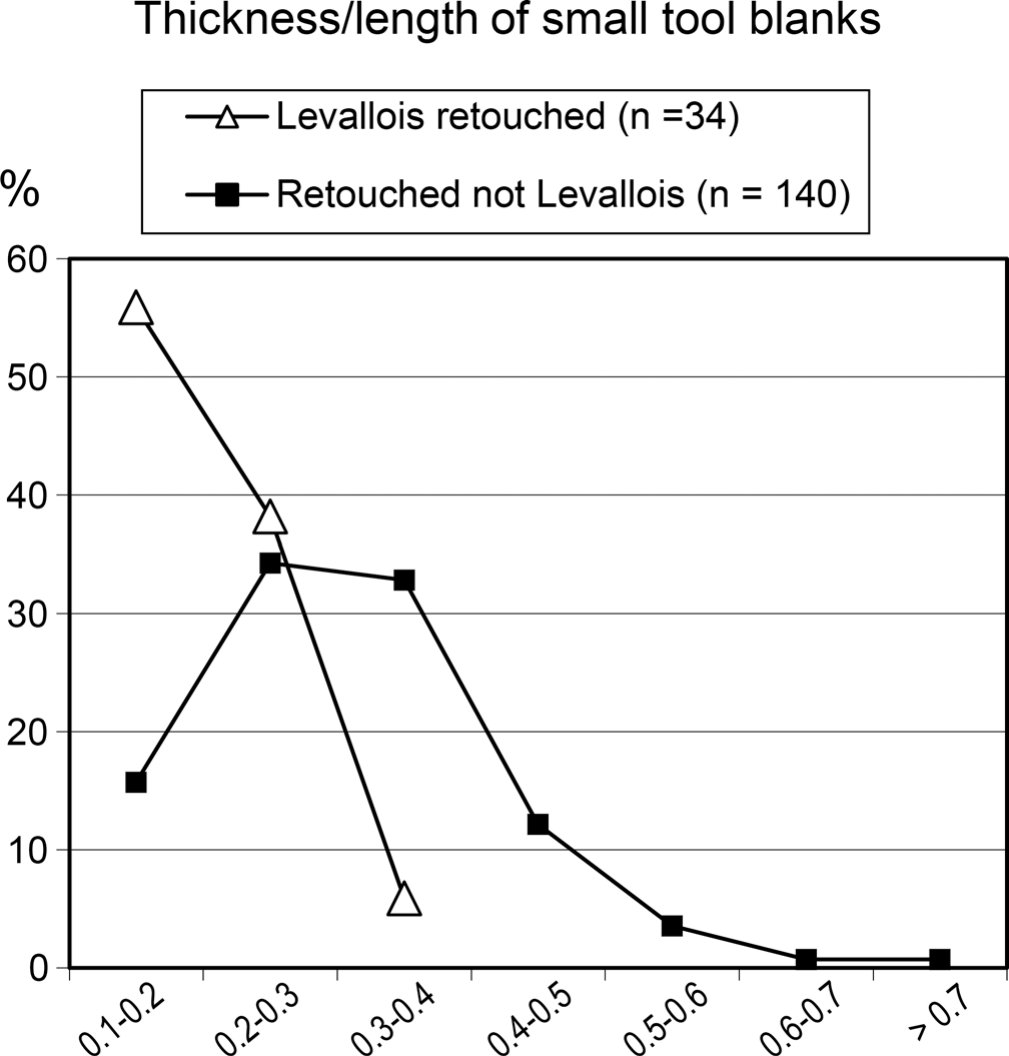
Frequency distribution of thickness/length ratios of Levallois retouched flakes and of small tools on ordinary flakes and on pebbles and cores.

The small tools of level *d* are characterized by a predominance of flake blanks (Table 10) over pebble or core blanks. The mean thickness/length of tools on pebbles and cores at Torre in Pietra level *m* and Castel di Guido is 0.4 thus we take the 0.4 as a reference point and related data is presented in Table 11. The prevalence of thin blanks is even more evident in Fig 22.

**Table 10:** Blanks of small tools^a^.

Categories	N	%
Flakes (157) and flake fragments (13)	170	80.2
Pebbles	21	9.9
Core, core fragments, chunks	21	9.9
Total	212	100

^a^Two indeterminate cases are excluded

**Table 11:** Thickness/length of small tool blanks at Torre in Pietra and Castel di Guido.

Assemblages	% Flake blanks with Th/L ≥ 0.4	% Pebble or core blanks with Th/L ≥ 0.4
Torre in Pietra level *m*	41.4	76.5
Castel di Guido	60.0	81.4
Torre in Pietra level *d*	15.5	50.0

Small tool blanks in perspective. It could be argued that the frequencies of small tools on thick blanks such as pebbles, core or flakes with thickness/length ratio ≥ 0.4 at Torre in Pietra level *m* and at Castel di Guido (Table 11) is biased by selective removal of light, thin pieces at both sites. Undoubtedly both sites were controlled by fluvial processes so the compared percentages should not be taken as absolute values. Nevertheless the level *d* artifacts were also scattered in fluviatile sediments of varying granulometry yet thin retouched flakes are very common (Fig 22). Thus we consider that the frquencies of thick blanks in the Acheulian assemblages in Latium are sufficiently great for consideration and may have a particular meaning, as indicated below.

Hafting. The choice of thin flake blanks (Fig 22) is a significant feature which is due to the predominance of the Levallois technology and most likely related to the emergence of hafting. To date the oldest direct evidence of hafting technology is from the site of Campitello Quarry in Tuscany (Central Italy) where birch-bark tar was found on the proximal part of two flint flakes associated with remains of *Paleoloxodon antiquus* and several micromammals. According to Mazza et al. [82–83] the rodent fauna dates the tool- and elephant-bearing bed of Campitello to a time shortly preceding the end of the Middle Pleistocene, a cool stadial episode before isotope stage 6. This implies most likely MIS 7.2, a cool-but not very cold- period within MIS 7, dated to 206–201 ka [84].

The small tool blanks of the Acheulian were thick and provided a relatively easy manual prehension; the flakes produced by the short reduction sequences of cores were almost all with some cortex which also facilitate manual prehension [85]. This is particularly evident at Castel di Guido where 91% of the flake blanks have cortex and partial bifaces with a cortical base are predominant. In level *m* 67% of the flake blanks have cortex; bifaces with a completely worked base are only 28.9% (13 of 45). The rest of the bifaces have a cortical base (n = 24) a cortical back (n = 3), a base by a natural thick fracture surface (n = 2) or a base only partly in cortex (n = 3).

In sum, the Levallois method produces thin and regular flakes that can be hafted easily, The biface shaping flakes were also thin but there is no evidence in the Acheulian of Torre in Pietra and Castel di Guido that they were retouched as small tools. Also in the Acheulian of Guado San Nicola (Molise, Italy) bifacial shaping flakes were rarely used as blanks [86]. It has been suggested that prior to hafting an important factor was manual prehension [85] and this would explain the abundance of bifaces with cortical bases, the preference for small tools on pebbles or thick flakes in the Latium Acheulian and the shift to Levallois technology and its rapid spread after hafting became important.

Most discussions on the origins of the Levallois technology have emphasized the timing, and whether and where it emerged from the Acheulian technology. The why remains less explored. One interesting explanation for its success was suggested by White et al. [87]. For them the Levallois was a method to produce regular multiple cutting edges in a more efficient use of lithic resources. Our hypothesis, based on a quantitative analysis of flake thickness- is not exclusive of White et al. suggestion.

**Fig 23:**
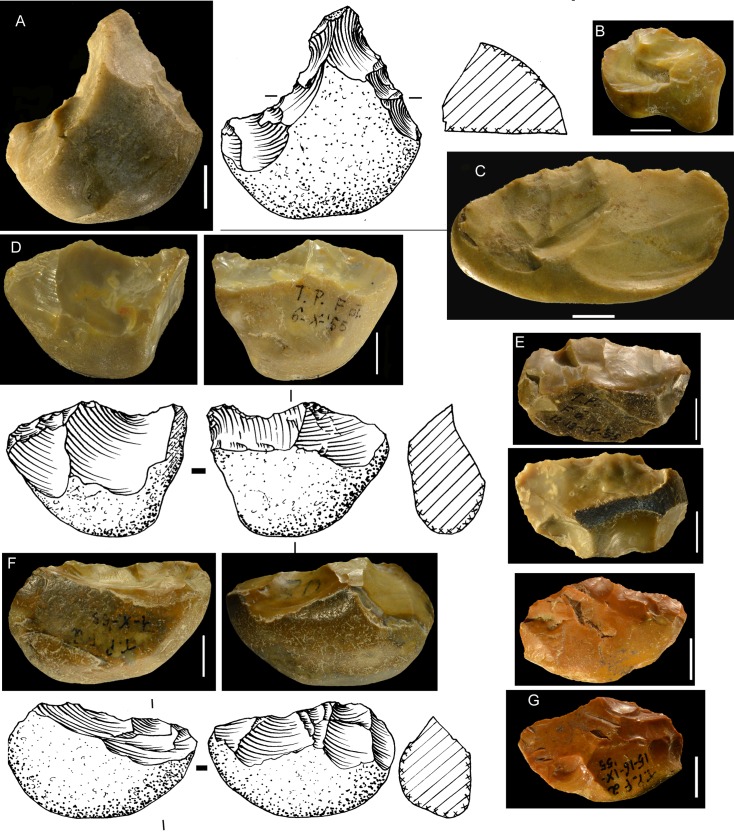
Torre in Pietra, level *d*, small tools on flint pebbles. (A) Convergent denticulate on pebble; the lower face is a natural fracture surface; (B, C) Unifacial retouch, C may have first functioned as a core; (D, F) Alternate bifacial retouch; (E) The denticulate edge is made using a single large removal on the opposite face as a striking platform; (G) Alternate scraper retouch on a pebble fragment with natural fracture surfaces on the lower face. Catalogue numbers: 85, 67, 59, 96, 56, 54, 9. Scale bar = 1 cm.

**Fig 24:**
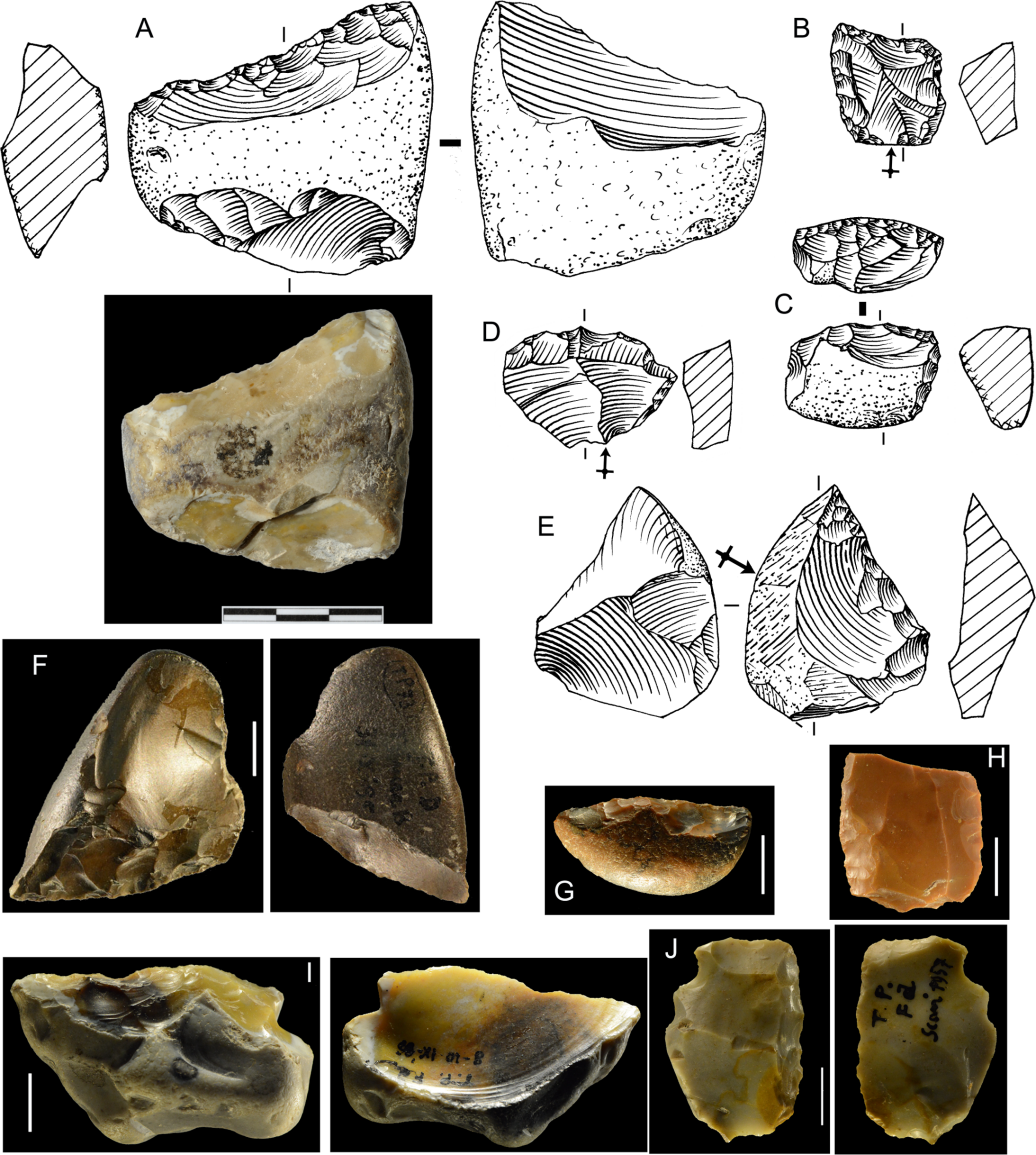
Torre in Pietra, level *d*, small tools on flakes and pebbles. (A, F) Scraper on pebble with a single narrow removal used as a striking platform for retouch (negative base); (B-E) are scrapers and denticulates on thick flakes with Th/L ≥ 0.4. B is convergent déjeté scraper, C is a transverse scraper, D is a denticulate and E is a side scraper converted to core afterward-unless the removals on the ventral face are for thinning and facilitating prehension; (I) scraper on a split pebble; the lower face is a flake, i.e. a positive blank, complement of a negative base (see text on Castel di Guido blanks of small tools); (G) scraper on a cortical flake; (H) broken scraper on a Levallois flake; (L) scraper with a denticulate opposite side and inverse distal retouch, on a Levallois flake. Catalog numbers: Pigorini 106017, 98, 106, 93, 10, 1, 147, 134, 23, 127. Scale bar = 1 cm.

Nevertheless a peculiar feature of level *d* is the continuous presence of blanks on pebbles and cores and on thick flake albeit at a much lower frequency than in the older Acheulian industries (Figs 23 and 24). The adoption of the new technology which defines the Middle Paleolithic is thus characterized by innovation combined with a degree of stability.

#### Debitage analysis

The level *d* assemblage consists mostly of flakes, both unretouched and retouched (Table 12). The Levallois debitage [55, 88–89] is the most characteristic component of lithic production in this level, which also includes a simpler kind of end- products obtained from unprepared cores with successive series of unidirectional removals (Table 13).

**Table 12:** Torre in Pietra, level *d*. Counts of flakes.

	Unretouched		Retouched^b^		Total	
Debitage	N	%	N	%	N	%
Cortical flake	38	15	12	7	50	12
Partly cortical ordinary flake ^a^	70	28	51	30	121	29
Non cortical ordinary flake	41	16	15	9	56	13
Flake with non conchoidal fracture	14	6	6	4	20	5
Flake with unidirectional parallel scars and cortical back or lateral and/or distal cortical edge	13	5	18	11	31	7
Flake with unidirectional parallel and orthogonal scars and cortical back or lateral and/or distal cortical edge	4	2	2	1	6	1
Flake with opposed parallel scars and cortical back or lateral and/or distal cortical edge	2	1	3	2	5	1
Levallois flake	55	22	38	22	93	22
Kombewa flake (1st or 2nd generation)	5	2	1	1	6	1
Other types of flakes	3	1	4	2	6	1
Flake from tool making or tool reworking	0	0	2	1	2	0
Undetermined flake	9	4	18	11	27	6
Total	254	100	170	100	423	100

^a^ “Ordinary” flakes are the generic product of any kind of core.^.^

^b^The total of retouched flakes (n = 170) is less than the total of small tools (n = 214) because not all small tools were on flakes (42 on pebble or core and 2 on indeterminate blank).

**Table 13:** Torre in Pietra, level *d*. Counts of cores.

Core type	N
Levallois core with a preferential (i.e. a single invasive) removal	10
Levallois core with centripetal removals	13
Levallois core with parallel removals	4
Undetermined Levallois core	8
Core with series of unidirectional parallel removals	19
Core with series of unidirectional convergent removals	2
Other type of core with series of unidirectional removals	4
Core with series of unidirectional removals, secant relatively to the largest plan of the volume	1
Core with centripetal removals (non Levallois)	4
Core with removals bearing non conchoidal fracture	3
Tested raw material	6
Core of undetermined type	4
Unclassified core fragment	18
Total	96

We exclude from these counts 14 cores and core fragments that have been retouched, nine of which are in fact negative or positive blanks and should be considered as just special kinds of blanks and not the product of a reduction sequence (see Table 10 and section on “Split pebbles and the bipolar technique”). Two other retouched cores are undetermined or unclassifiable core fragments. The remaining three retouched cores are centripetal (1) or pebbles with unifacial removals and some secondary retouch (Fig 23C).

#### Distinguishing Levallois from non-Levallois cores

**Fig 25:**
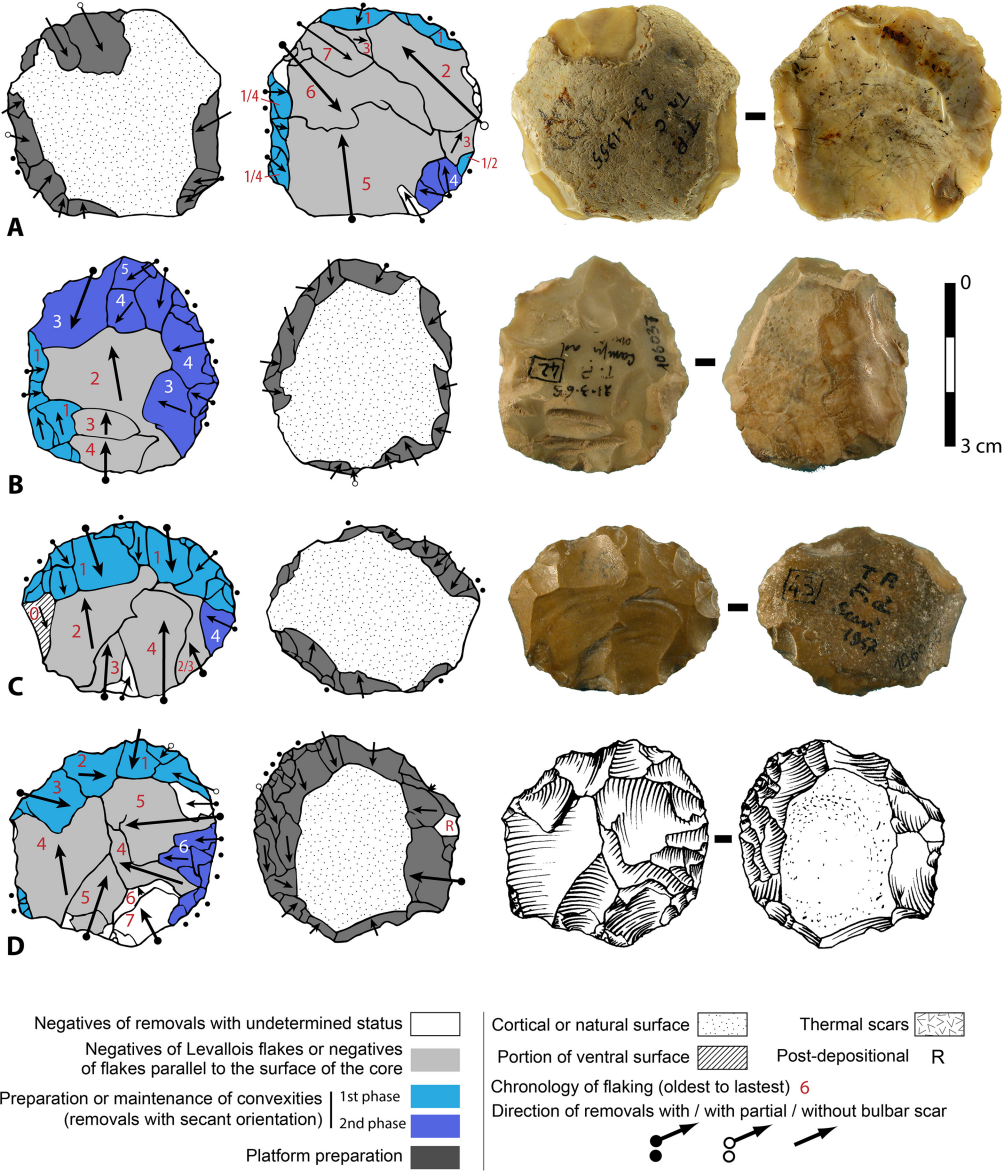
Torre in Pietra, level *d*. **Levallois cores.** The chronology of flaking has been established for each core through observations of the overlap of negatives with a magnifying glass under oblique light. Catalog numbers 30, 106037, 106038, 106039.

**Fig 26:**
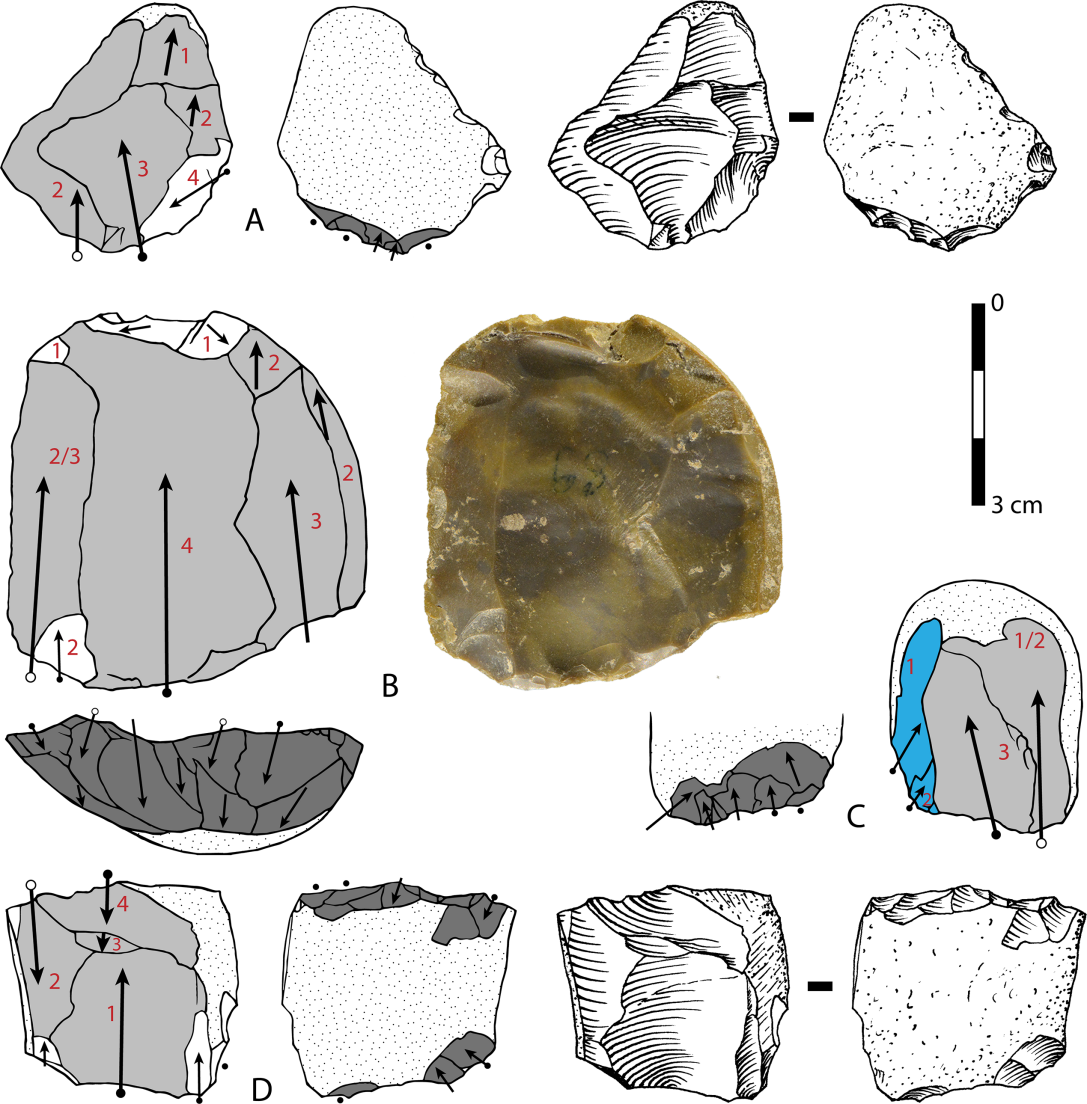
Torre in Pietra, level *d*. **Non-Levallois cores.** Caption and symbols used in drawing as in Fig 25. Catalog numbers 48, 43, 36, 51.

We follow the definition of Levallois cores proposed by [55, 87–89]. The 35 Levallois cores represent 51.5% of the total of determined cores (n = 68). Contrary to [81] we do not class as Levallois cores that have a single debitage surface with multiple removals (either parallel or opposed or centripetal) but without any scars that would indicate preparation of the debitage surface or maintenance of convexities (Figs 25 and 26). This is the case of core in Fig 26B which has no inclined scars on the periphery of the debitage surface which might suggest control of the lateral and distal convexities. Some of these non-Levallois cores may have produced flakes typologically classifiable as Levallois, similar to Levallois flakes that do not preserve on their dorsal face negatives of removals controlling the convexities (see type L3U, Fig Y in S1 File). This convergence in the morphology of the product has been described long ago [46]. We are aware of this problem and we have considered as Levallois flakes that fall within the typological definition of Levallois flakes.

#### The volumetric conception of Levallois cores

The core has two asymmetrical convex surfaces (the debitage surface and the striking platform) which define a plane of intersection. On the debitage surface there are two kinds of negatives: removals that are parallel or sub-parallel to the plane of intersection of the two surfaces and are elongated, spanning the middle or most of the debitage surface, and others that are shorter, centripetal and oblique with respect to the plane of intersection. The first are negatives of predetermined Levallois flakes; the second are the negatives of removals that shape the core and maintain the convexities. The chronology of removals clearly shows that the extraction of Levallois flakes are preceded by preparation of convexities (filled in light blue on Fig 25 and Figs Z, AA in S1 File) and also at times followed by a partial re-preparation (filled in dark blue). This alternation is typical of the Levallois volumetric conception.

The maximum length of abandoned Levallois cores is always less than 50 mm; the reduction sequence is stopped when the length or width of products is not smaller than 28 and 22 mm (Fig AB in S1 File).

The abandoned Levallois cores show three types of predetermined removals: 1. a single invasive “preferential” scar (N = 10; Fig 25B) (Fig Z: B-D in S1 File and Fig AA: B in S1 File); 2. multiple negatives that are either parallel (N = 4; Fig 25C) (Fig Z: A, F in S1 File and Fig AA: D in S1 File) or 3. centripetal (N = 13; Fig 25A and 25D) (Fig AA: A, C in S1 File). Eight cores definitely Levallois but broken could not be classed in this manner.

Does this mean that two different methods (preferential and recurrent) were used? Not exactly. Several elements suggest that cores with a single invasive removal are the final or accidental step of a recurrent Levallois method. First, the length/width distribution of cores (Fig AB in S1 File) is not scattered but tends to cluster. The length and width means in Table 14 show that the Levallois cores with a single invasive removal are similar in size to the abandoned Levallois cores with centripetal removals. Note that the standard deviation is always less than a fourth of the mean.

**Table 14:** Torre in Pietra, level *d*. Length and width of Levallois cores.

	Length (mm)		Width (mm)	
	Mean	SD	Mean	SD
Levallois core with a single invasive scar (N = 10)	34.1	5.8	29.6	6.2
Levallois core with centripetal scars (N = 13)	36.8	5.6	29.6	6.0
Levallois core with parallel scars (N = 4)	40.7	6.0	34.8	4.1

In northwestern Europe when both preferential and recurrent methods were used, the size of preferential cores was definitely larger than that of recurrent cores, e.g. at Mesvin IV [57].

**Fig 27:**
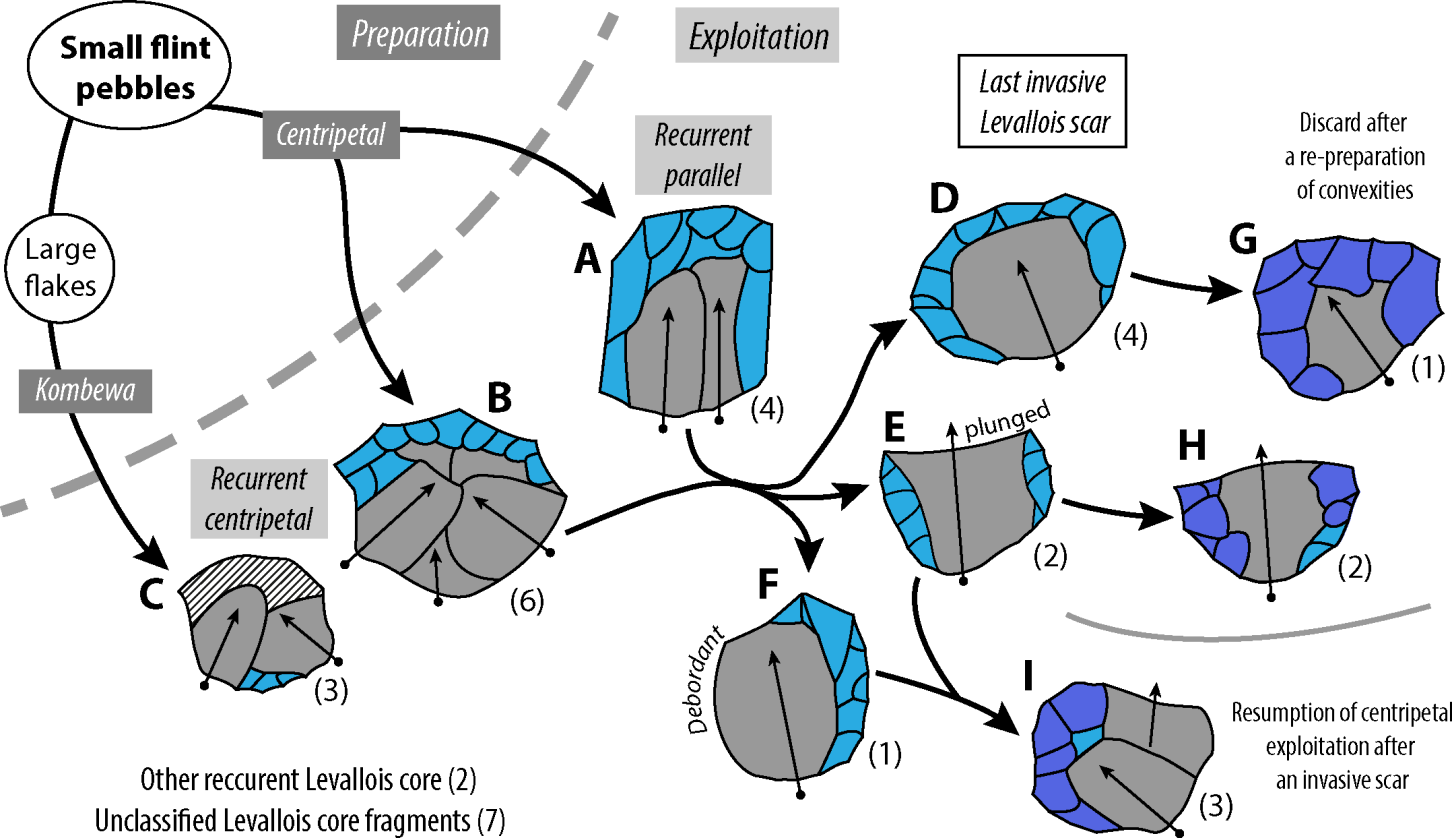
Torre in Pietra, level *d*. **Schematic representation of the Levallois debitage.** Counts of cores in each type are in parenthesis. Codes of colors are as in Fig 25.

Second, a detailed analysis of the reduction sequence shows that on four of the cores with an invasive removal, the flake is plunging (Fig 27E and 27H) so that its invasiveness is likely to be the result of a knapping accident. On three other cores, after the accident, knapping continued producing one or more smaller Levallois flakes (Fig 27I), thus the cores have been defined as Levallois with scars of several predetermined flakes (Fig Z: A in S1 File). Based on these observations, we believe that the Levallois cores with a single invasive removal are not the result of the preferential method, as it has been described at the sites of Hermies, Ault and Fitz-James in northern France [90–92] but correspond to a final or semi-final stage of recurrent Levallois debitage on small pebbles. At any rate, it is impossible in this industry to distinguish Levallois flakes from a preferential method from those produced by the recurrent method, especially flakes of type L1C (Fig Y in S1 File).

In sum, the Levallois production mode used at Torre in Pietra is the recurrent parallel or centripetal method [88–89]. We note that the Levallois flakes which have only preparation scars (type L1C in Fig Y in S1 File; about 14% of Levallois flakes) indicate that debitage began by a centripetal preparation. Some residual portions of ventral face on 6.4% of Levallois flakes (Table A in S1 File) as well as four cores (Fig 25C) show that the debitage began on the ventral face of large flakes. Most cores show particular care in controlling peripheral convexities using small secant centripetal flakes. In contrast, “débordant” flakes [93] were rarely used for control of convexities (only 5.4% of the Levallois flakes). The few (3.2%) Levallois flakes with convergent scars result from a centripetal debitage documented in some cores (Fig 25D). Cores and flakes with parallel bidirectional opposed scars (types L2B, L3B; Fig Y in S1 File; Table A in S1 File) are rare compared to those with unidirectional parallel scars, not surprisingly if we consider the rather small size of the used pebbles.

It is not easy to define the relation between the recurrent centripetal and recurrent parallel methods. Levallois cores with parallel scars are few (n = 4) compared to those with centripetal or invasive scars; yet Levallois flakes with parallel scars are as numerous as those with centripetal scars (Table A in S1 File). Since the flakes with parallel scars are a bit bigger (Table 15), it is possible that in some cases debitage started in the recurrent parallel mode and was then continued in the centripetal mode, as suggested by [81]. However this cannot be formally established.

**Table 15:** Torre in Pietra level *d*. Length and width of complete or almost complete Levallois flakes.

	Length		Width	
	Mean	SD	Mean	SD
Levallois flakes with parallel predetermined dorsal scars	36.7	8.9	25.5	5.2
Levallois flakes with centripetal predetermined dorsal scars	28.4	7.0	25.4	5.4

The use of two different exploitation methods resulted in some variability in the size of products (Figs AC and AD in S1 File). In most cases flake length ranges from 20 to 40 mm, as expected given the small size of knapped pebbles. A few flakes with length ≥ 50 mm are isolated occurrences.

#### Non-Levallois debitage

Aside from Levallois debitage, a good number of cores were knapped in short sequences of unidirectional parallel or subparallel, convergent or centripetal removals without any special preparation of the debitage surface or shaping of the core (Fig 26). In a few cases (n = 4) a partial preparation of convexities, restricted to one side of the core, can be observed (Fig 26C). In some cases a single series of flakes were extracted from one of the large faces of the core using a unique striking platform. The flakes so produced have unidirectional dorsal scars and cortical abrupt or oblique back. Some with a facetted platform resemble Levallois flakes. When there are several series of removals of parallel or sub- direction, the flakes may have been produced from the same debitage surface but from different striking platforms or on different debitage surfaces. In this case the flakes have no specific pattern and we class them in the “ordinary” category. The few cores which are worked with a unifacial centripetal organization of removals cannot be confused with the Levallois centripetal method (Fig Z: E in S1 File). This kind of simple debitage occurs throughout the Paleolithic [94–95].

A few flakes (n = 20, of which 6 are retouched) are characterized by a non-conchoidal fracture, without the opposing bulb and crushing or splintering typical of the bipolar technique (see “Split pebbles and the bipolar technique”).

#### Concluding remarks on Level *d*

The Levallois debitage from level *d* is not the oldest occurrence of the Levallois in Europe [87, 96]. It is fully within the definition of the Levallois and is similar to later Middle Paleolithic occurrences. It presents some interesting features: 1.The small size of the raw material did not cause a shortening of the reduction sequence. On the contrary, there is systematic management of convexities in Levallois flakes, even more carefully developed than in later Middle Paleolithic industries. 2.The predominance of the centripetal methods is not a consequence of the raw material small size. In fact, the centripetal method is frequent in late Middle Paleolithic industries where large nodules of flint were available and were exploited, for instance at Corbehem and Henin-sur-Cojeul [89, 97].

Strikingly, the centripetal method is not frequent in early Middle Paleolithic industries from SW France, such as Cantalouette 1, Londigny or Les Bosses [98–100]. There it is found in association with Acheulean-style bifaces and other kinds of debitage. At La Borde and Coudoulous also in SW France the centripetal Levallois is found together with the discoidal debitage on quartz [101–102]. In other words, the raw material of level *d* limited the size of the debitage products but had limited effect on the overall system of lithic production.

A peculiar feature of level *d* is the continuous presence of small tools blanks on pebbles and cores and on thick flake albeit at a lower frequency than in the older Acheulian industries. The adoption of the new technology which typifies the Middle Paleolithic can be defined as innovation combined with a degree of continuity.

We observe a comparable, yet different, phenomenon in the early Middle Paleolithic assemblages from Europe where the Levallois is the dominant method of production but is associated with a few bifaces of Acheulian style (not like the bifaces of the Mousterian of Acheulian Tradition). This is the case at Mesvin IV in Belgium, of Pontnewydd in England, of Bapaume and Piègu in northern France [103–106].

In Latium continuity between the Lower and Middle Paleolithic is defined by small thick tools on pebbles or flakes. To the north stability in cultural transmission is indicated by the retention of bifaces.

## Conclusions

This research has allowed us: (a) to obtain ^40^Ar/^39^Ar dates and U-series-ESR dates which support correlation to marine isotope stages. Prior to this work the Torre in Pietra sequence was not radiometrically dated. The reworked pumices found in level *m* and interpreted as derived from the Red Tuff with black scoria only provided a post quem date of 431ka; (b) to integrate the dating results with geomorphological and geochronological data of the coastal area of Latium thus placing the Torre in Pietra succession into a clear chronostratigraphic scheme. The Acheulian is now securely dated to the end of MIS 10 and the Middle Paleolithic of level *d* to the early part of MIS7 between 270 and 240 ka; (c) to show that the hominins at Torre in Pietra and Castel di Guido had a clear understanding of the specific qualities of flint which came in the form of rather small pebbles and was deemed desirable for the making of small tools. The almost exclusive preference for flint in the presence of other raw materials, and the preference for thick blanks with cortex including negative blanks obtained by splitting a pebble in half by direct percussion imply a technical repertory that was shared by the Acheulian craftsmen in the region and can also be observed in later assemblages. To what extent a significant factor was the choice of raw material (small flint pebbles) can only be decided on the basis of further analyses of assemblages from other regions; (d) to suggest that the Levallois technique of the Middle Paleolithic assemblage which is characterized by the production of thin flakes without cortex is an innovation likely to correlate with the emergence of hafting. Interestingly the oldest direct evidence of hafting is from the site of Campitello in Tuscany dated to end of MIS 7, about 200 ka; (e) to suggest that the recurrence of certain tool-making habits, in spite of the introduction of an innovative technique underlies the importance of learning and conformity in the behavior of Neandertals.

## Supporting Information

S1 File Figures and Tables. (PDF)

S2 File ^40^Ar/^39^Ar dating. (PDF)

S3 File U-series and ESR dating. (PDF)

S4 File Permissions from copyright holders. (PDF)

S5 File Lithic analysis. (PDF)
