# Supplementary material for: The Acheulian and Early Middle Paleolithic in Latium (Italy): Stability and Innovation
Source: PLoS One. 2016 Aug 15;11(8):e0160516. doi: 10.1371/journal.pone.0160516 (PMC4985512; doi:10.1371/journal.pone.0160516)
Supplement: S1 File — (PDF) [file pone.0160516.s001.pdf]

# Supporting Information

## The Acheulian and early Middle Paleolithic in Latium (Italy): Stability and Innovation

Paola Villa*, Sylvain Soriano, Rainer Grün, Fabrizio Marra, Sebastien Nomade, Alison Pereira, Giovanni Boschian, Luca Pollarolo, Fang Fang, Jean-Jacques Bahain

*To whom correspondence should be addressed. E-mail: villap@colorado

## **S1 File.** Figures and Tables

This PDF file includes:

- Figures A-Z, AA-AD
- Table A
- References

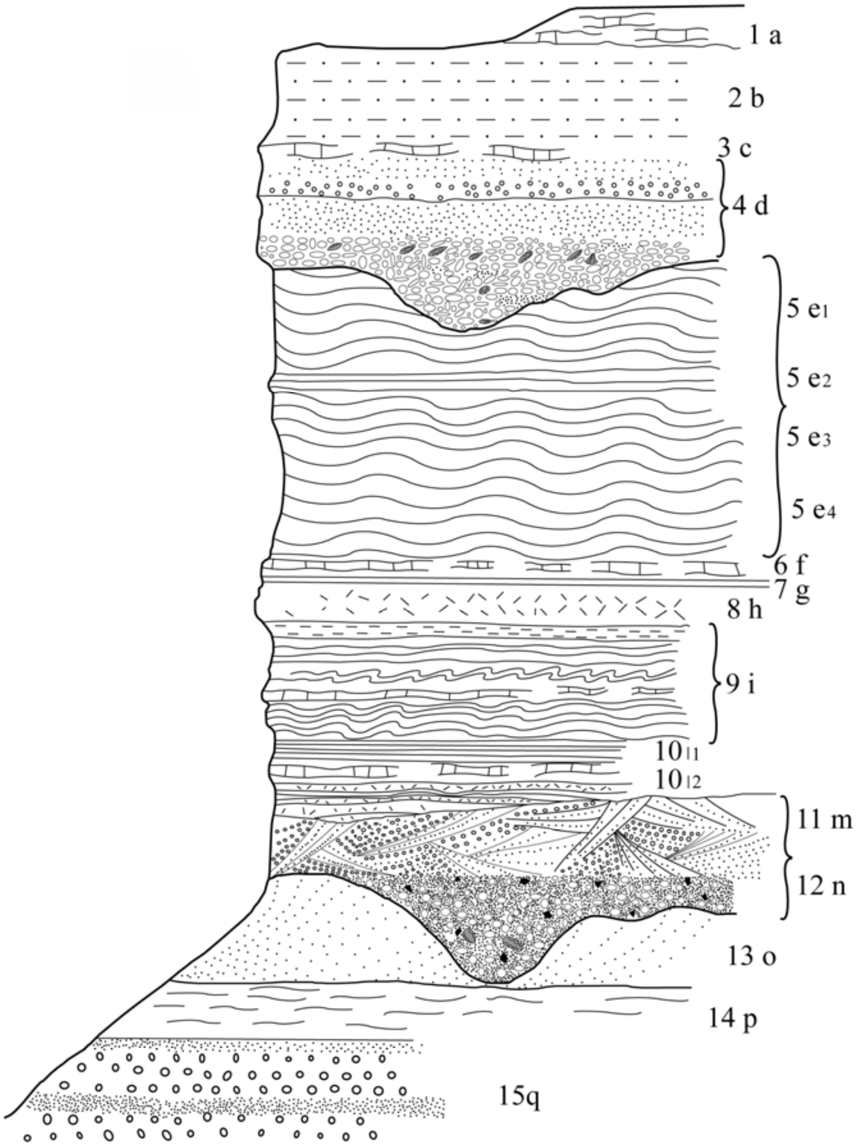


Figure A. Torre in Pietra, stratigraphic section published in 1978. Modified from Malatesta [1].

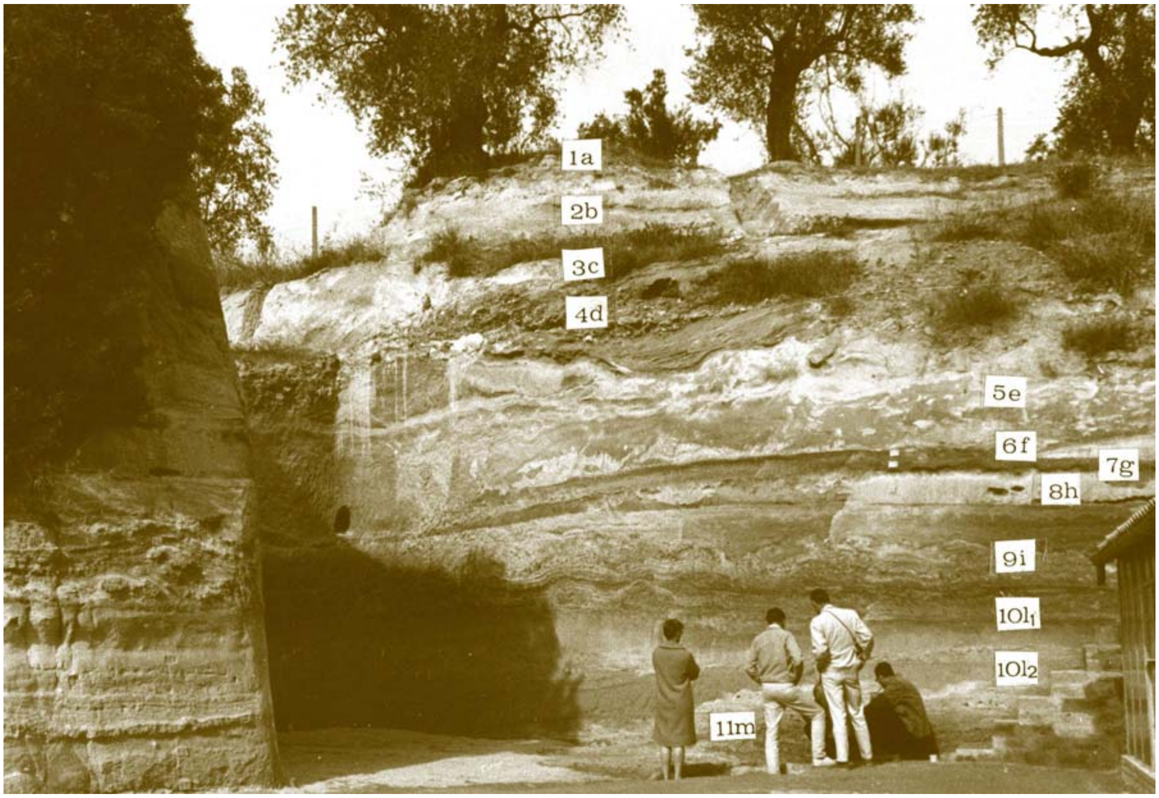


Figure B. Torre in Pietra. Main section of excavation 1955-1964 (reprinted from [1] with permission from the Italian Institute of Human Paleontology).

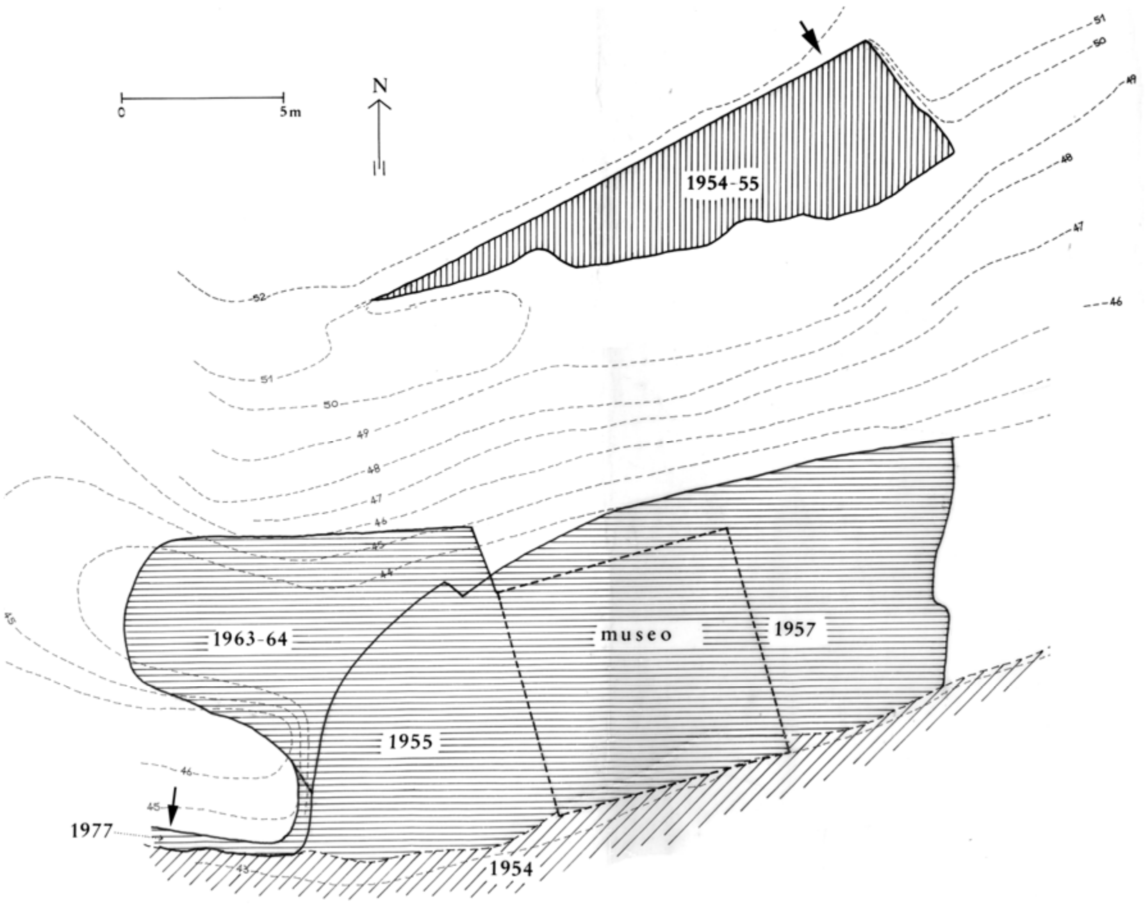


Figure C. Torre in Pietra. Plan of the excavation. The arrows indicate the position of sections cleaned in 2012-2013. The building called Museum protected a portion of level *m* left in situ; the material was later removed to avoid degradation and transported to the Pigorini Museum. Modified after [2].

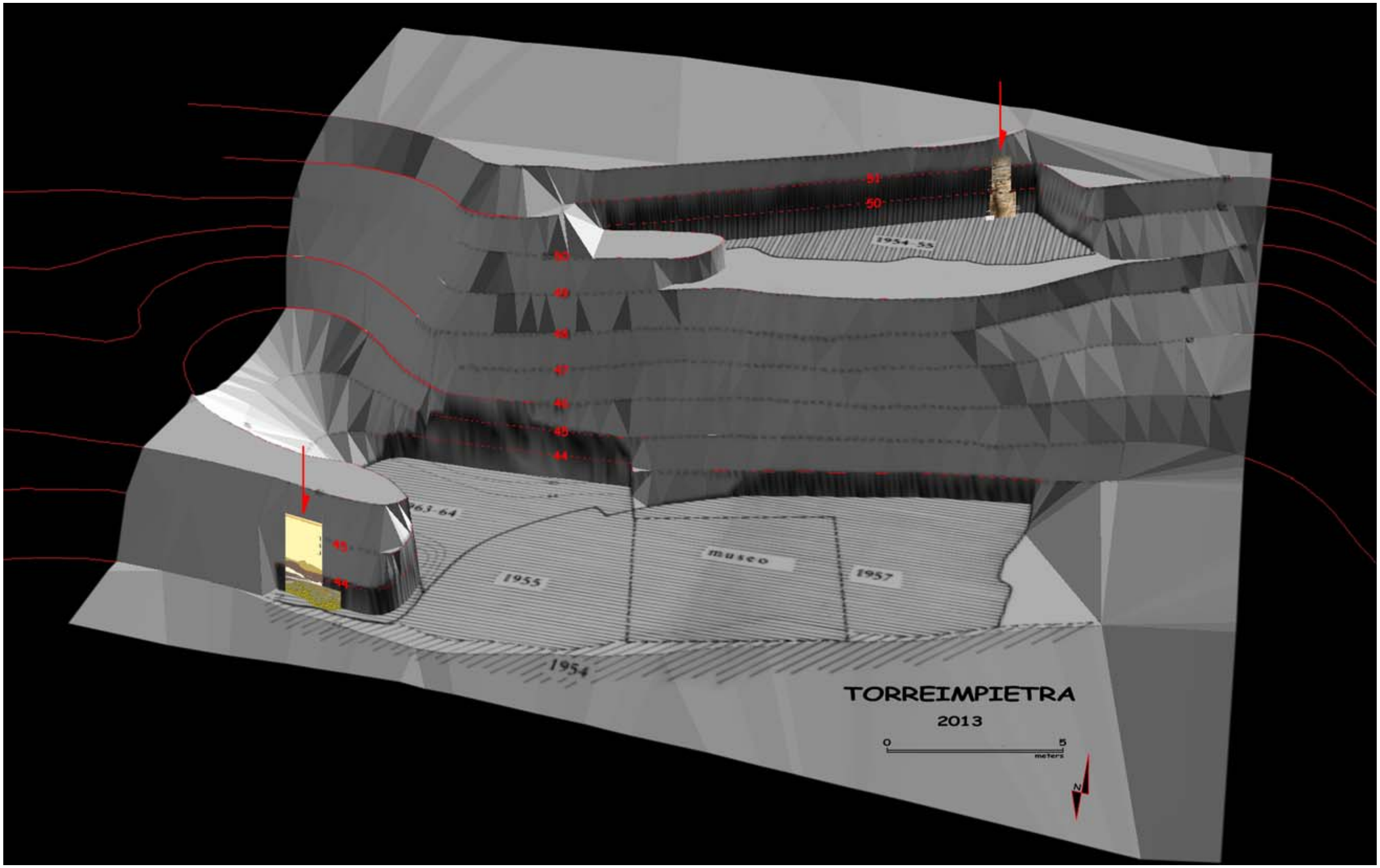


Figure D. 3-D view of the Torre in Pietra excavation and location of cleaned sections (courtesy of Luca Galletti).

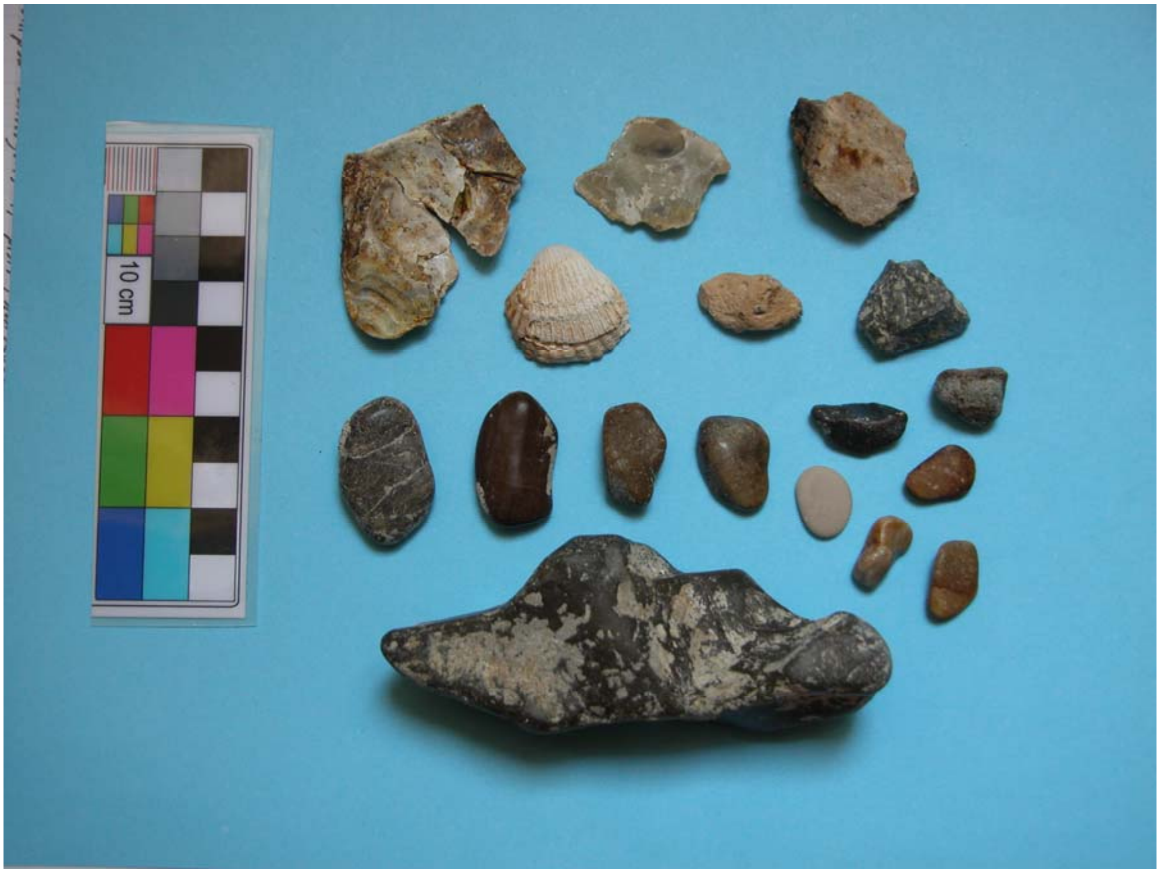


Figure E. Natural components of the conglomerate at the base of the Aurelia Formation (layer 12 n of Malatesta) with pebbles, shell fragments and one intact valve of *Cerastoderma* reworked from the underlying layers.

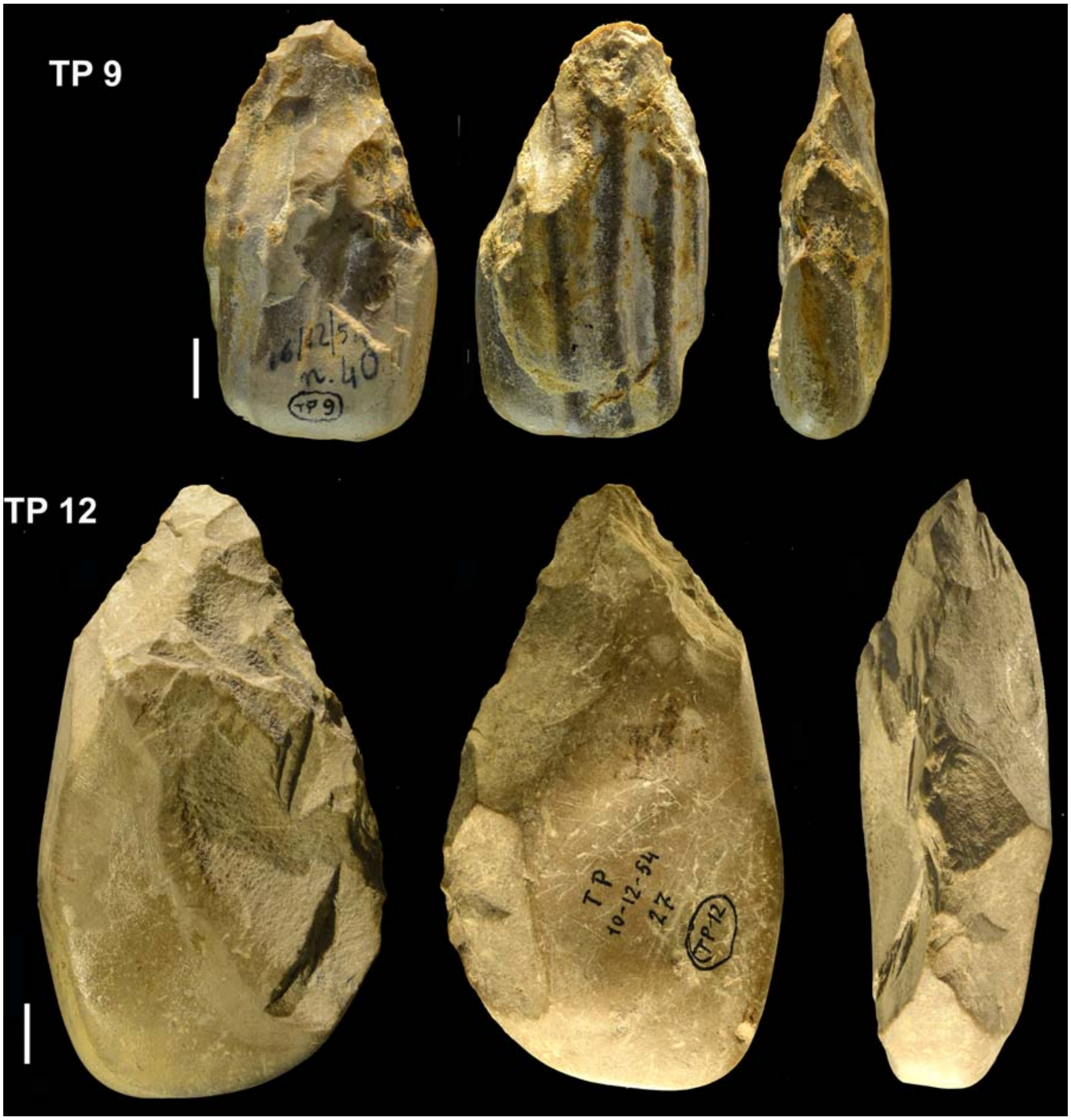


Figure F. Bifaces TP 9 of siliceous limestone and TP 12 of sandstone, both fresh. Scale bar = 1 cm.

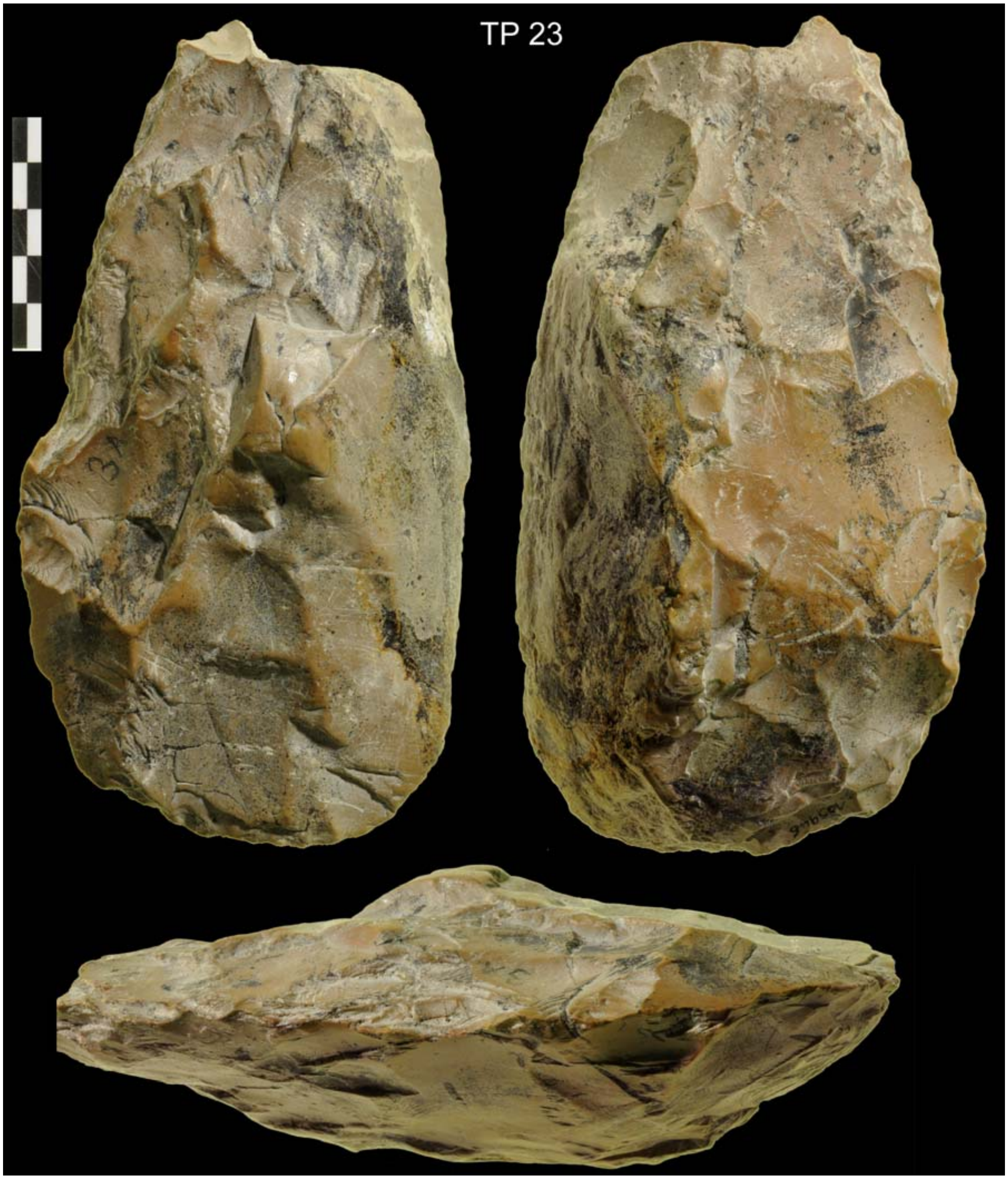


Figure G. TP 23, lithographic limestone. Face 1 (left) is abraded, face 2 (right) is slightly abraded.

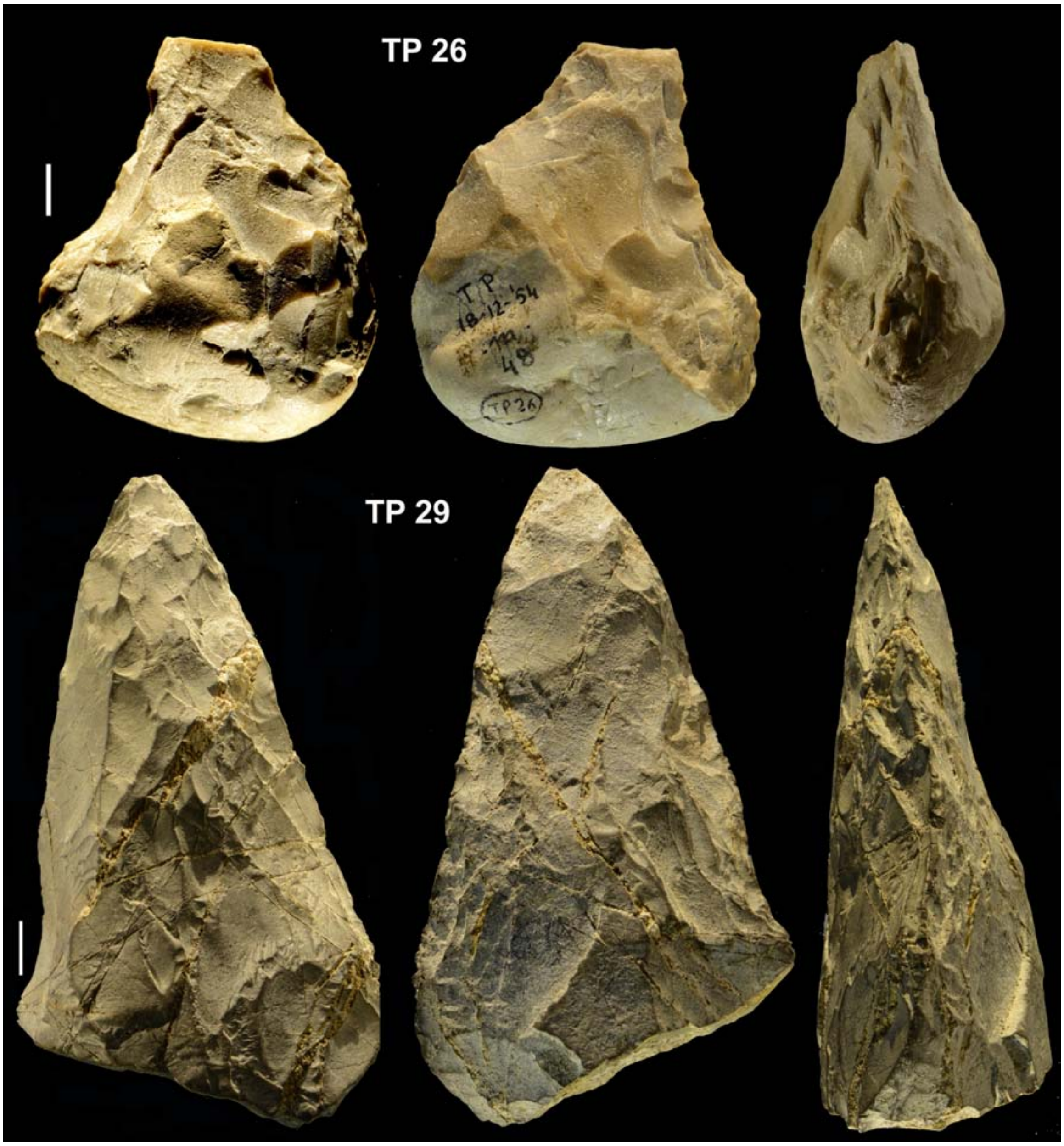


Figure H. TP 26 coarse flint, fresh.  TP 29 micritic limestone, only slightly abraded. Scale bar = 1 cm.

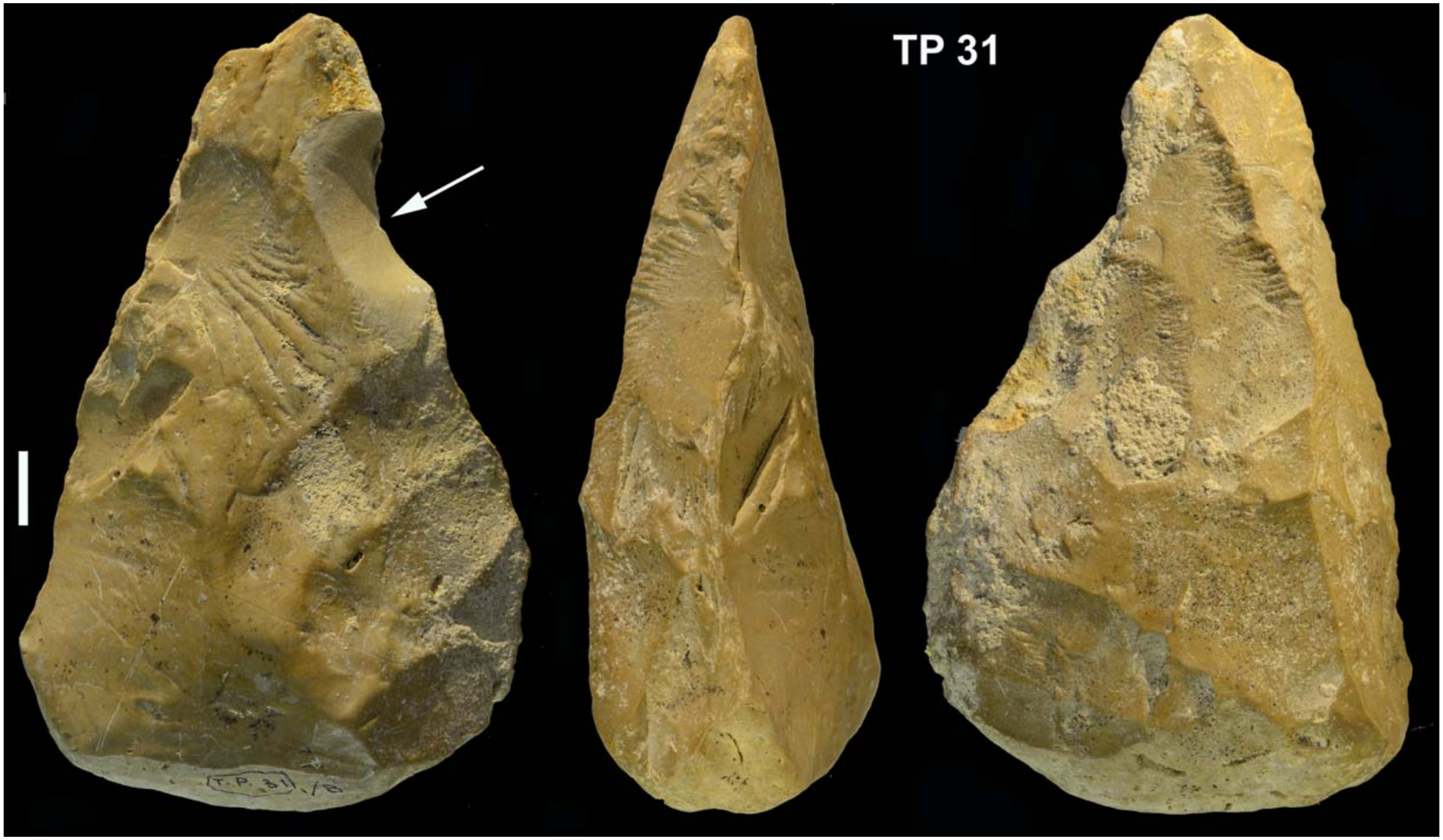


Figure I. TP 31, siliceous limestone. Abraded on face 1, slightly abraded on face 2. The arrow points to recent excavation damage. Scale bar = 1 cm.

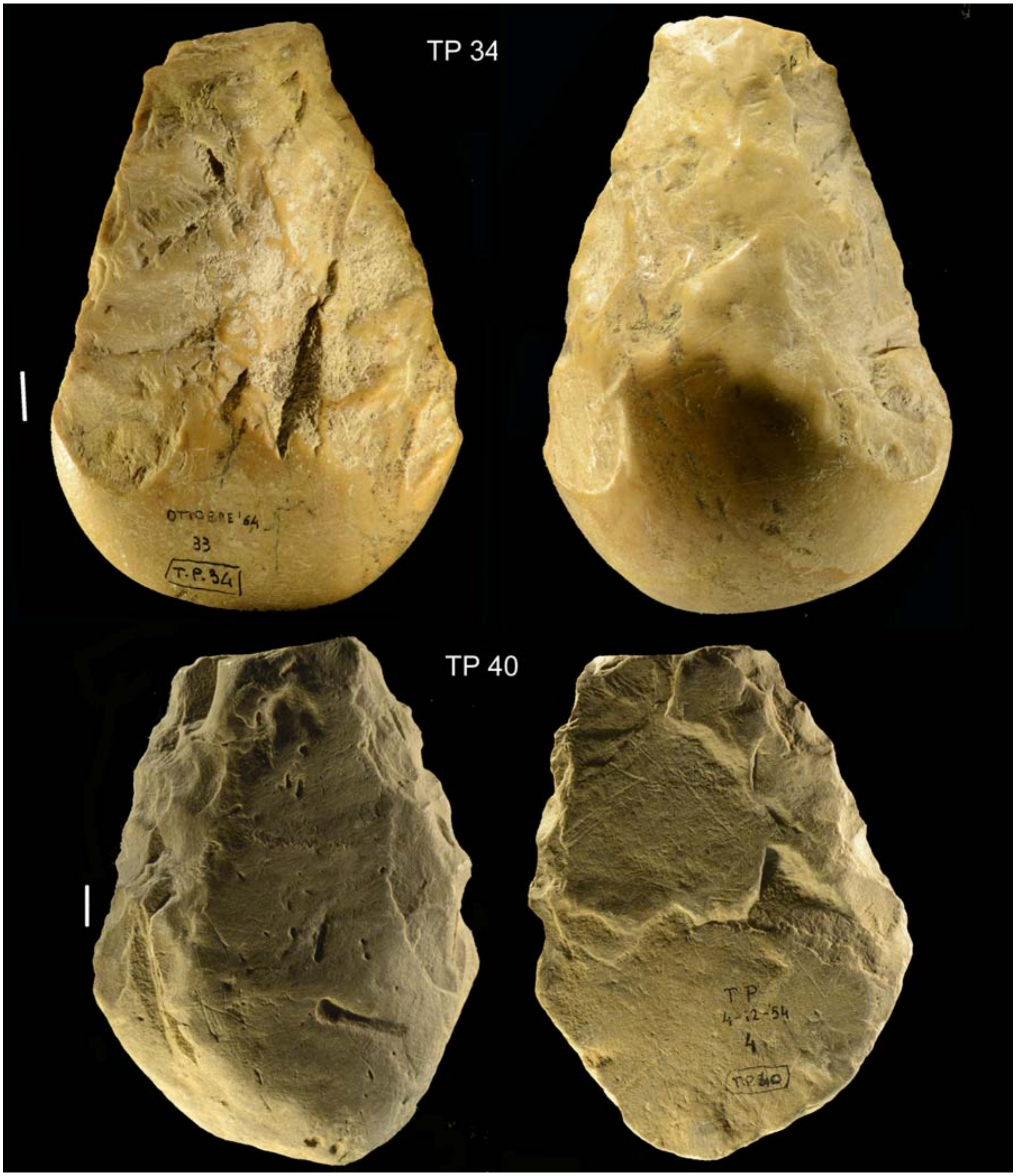


Figure J. TP 39, TP 40. Both of limestone and both abraded. The tip is missing (ancient break) on both. Scale bar = 1 cm.

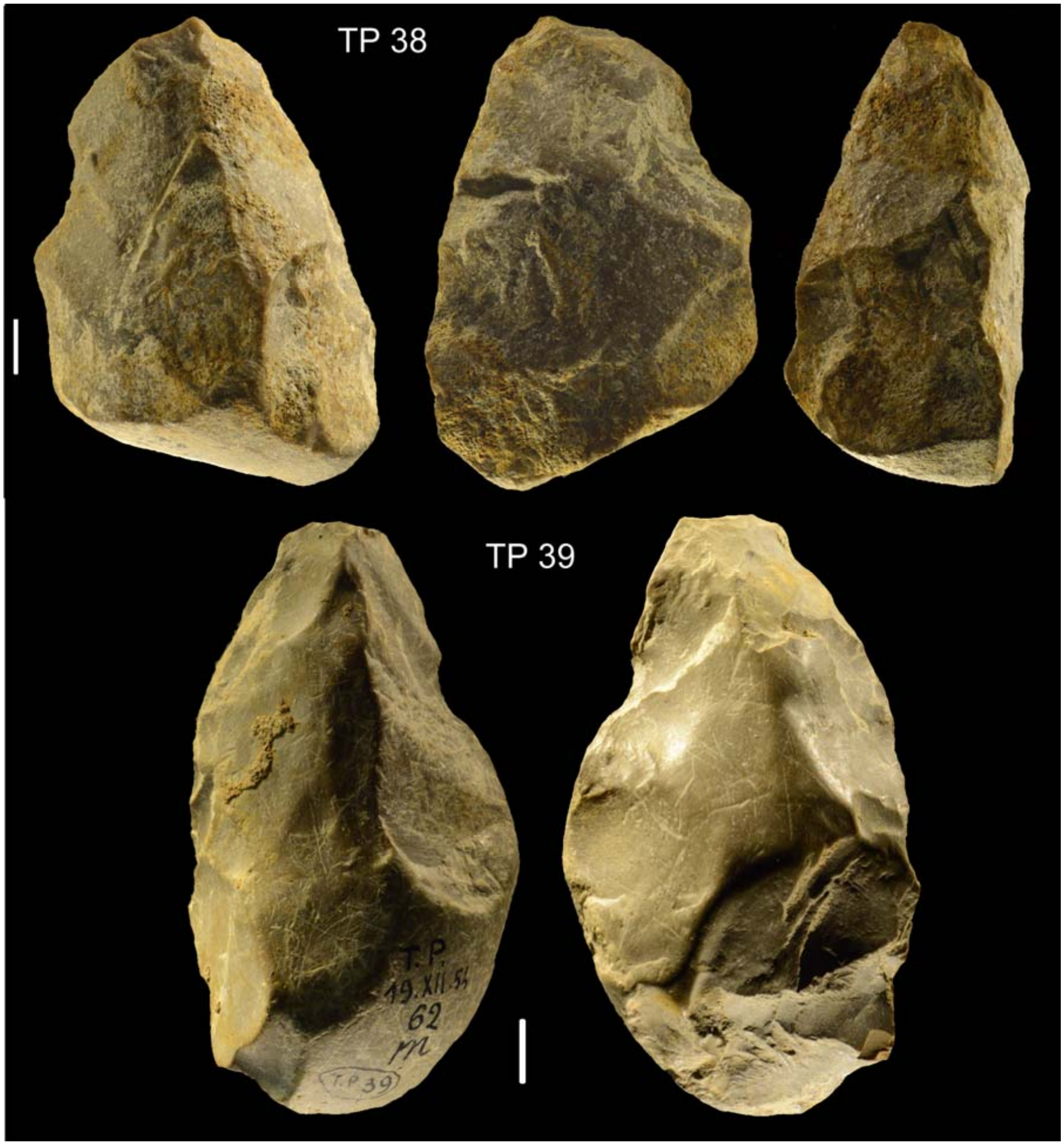


Figure K. TP 38 (quartzite) is slightly abraded. It is a very thick rough-out, abandoned after a few removals. TP 39 (Limestone) is abraded with fresh removals at the tip and at the base. Scale bar = 1 cm.

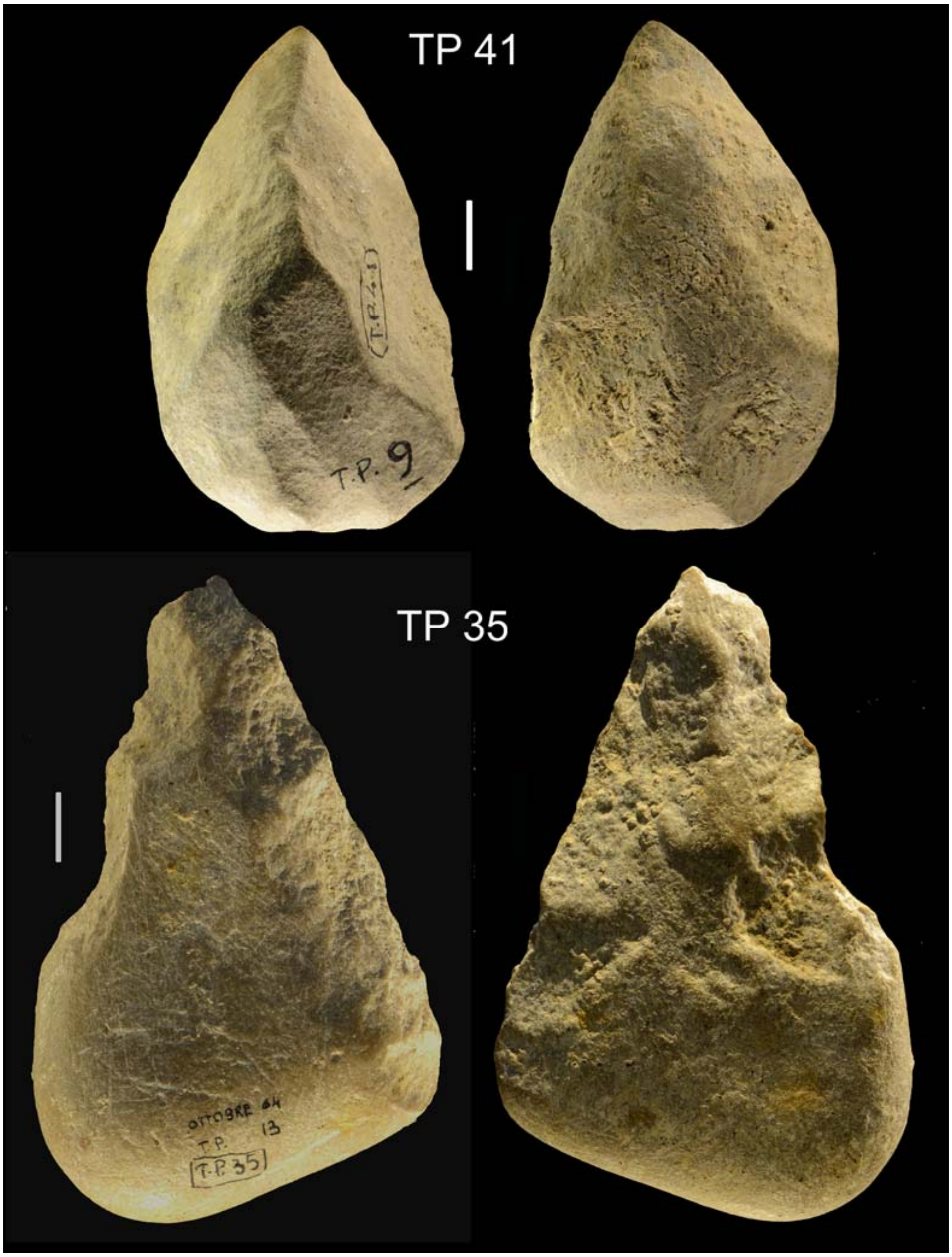


Figure L. TP 41, TP 35, both of limestone. TP 41 is extremely abraded, TP 35 is abraded. Scale bar = 1 cm.

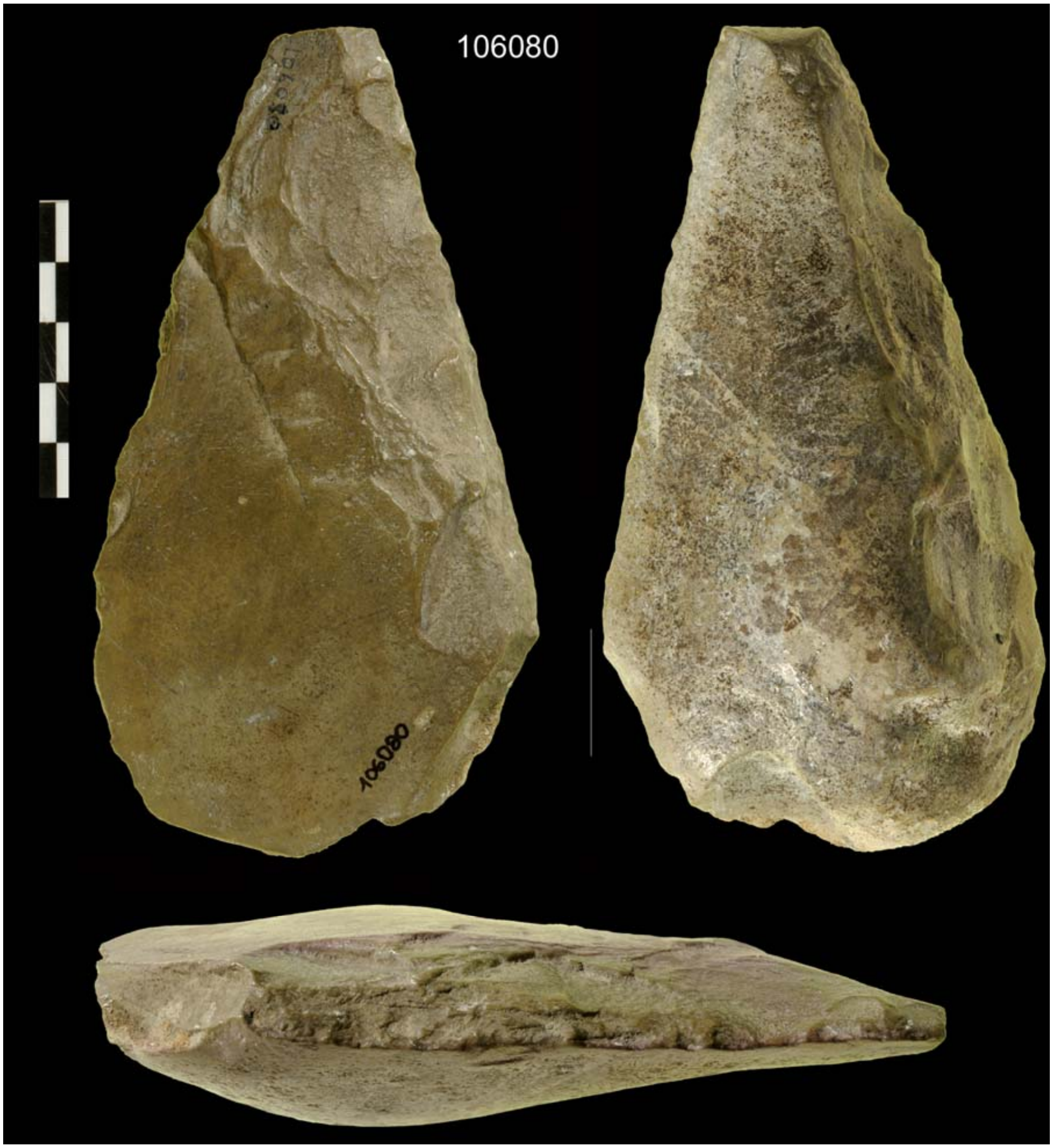


Figure M. Pigorini Museum catalogue number 106080. This slightly abraded limestone biface is on flake, the only one of this kind at Torre in Pietra. The tip is broken by an ancient fracture.

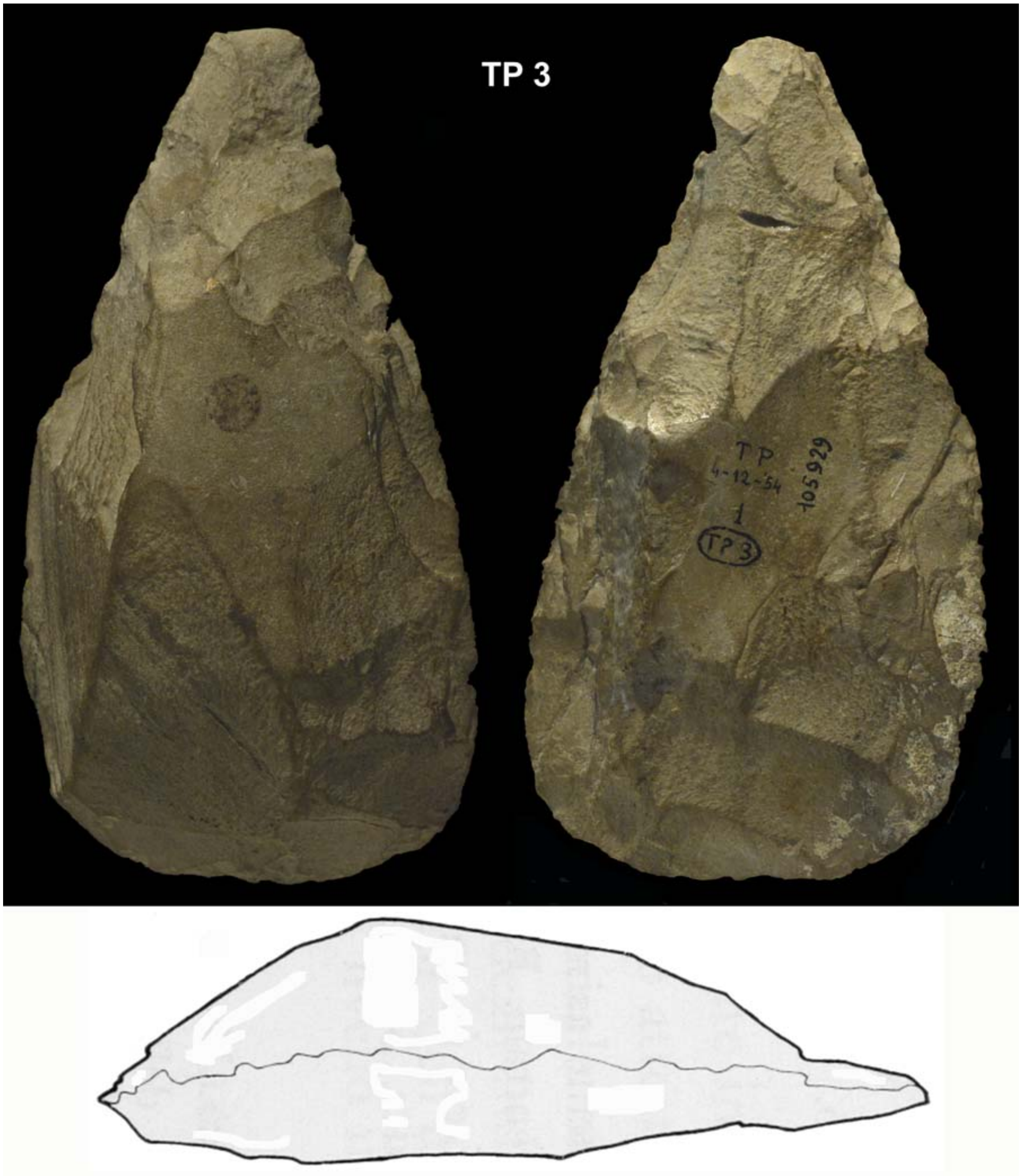


Figure N. TP 3, limestone biface, fresh to very slightly abraded, L = 15.5 cm.

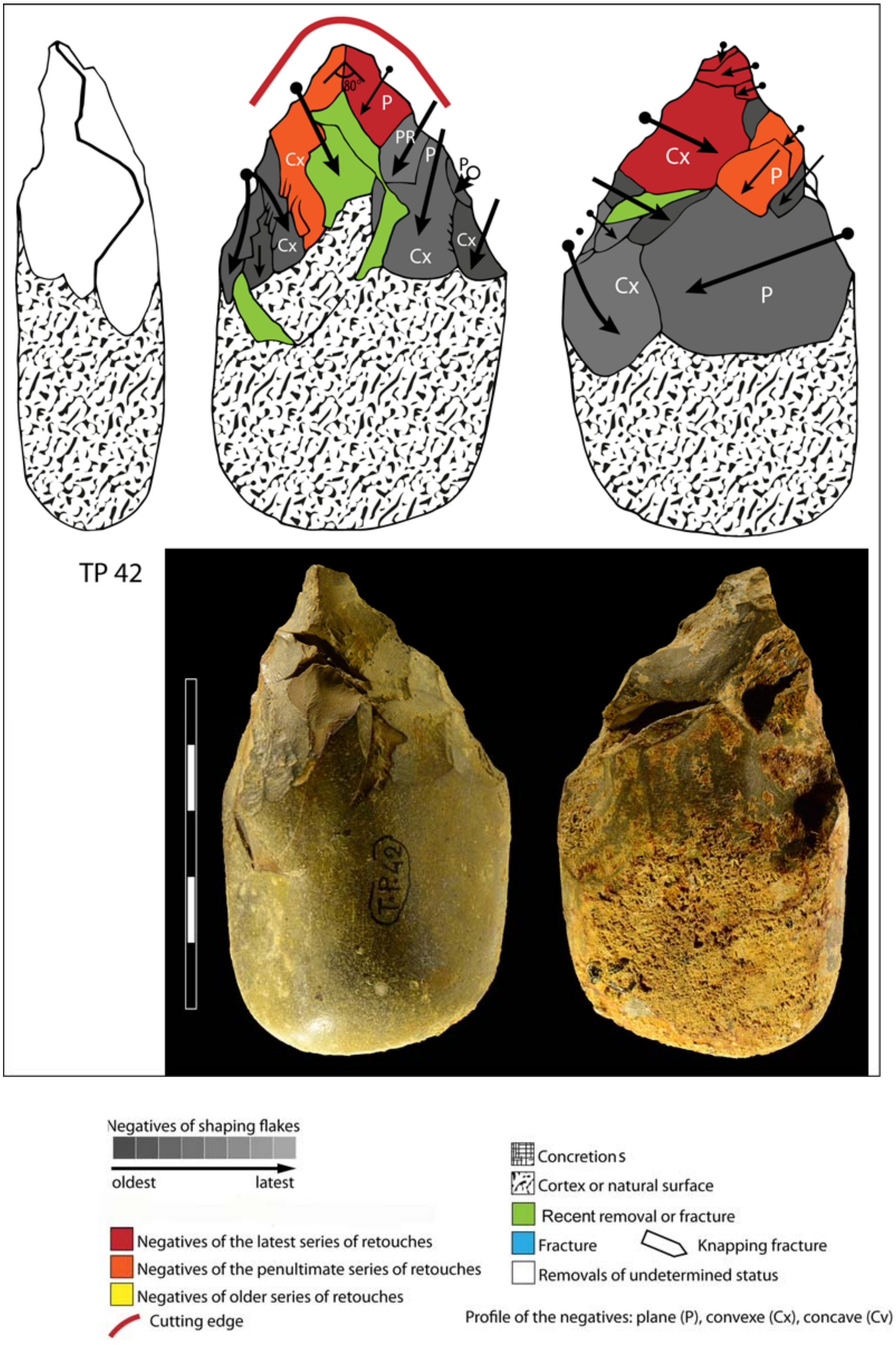


Figure O. TP 42, fresh, lithographic limestone.

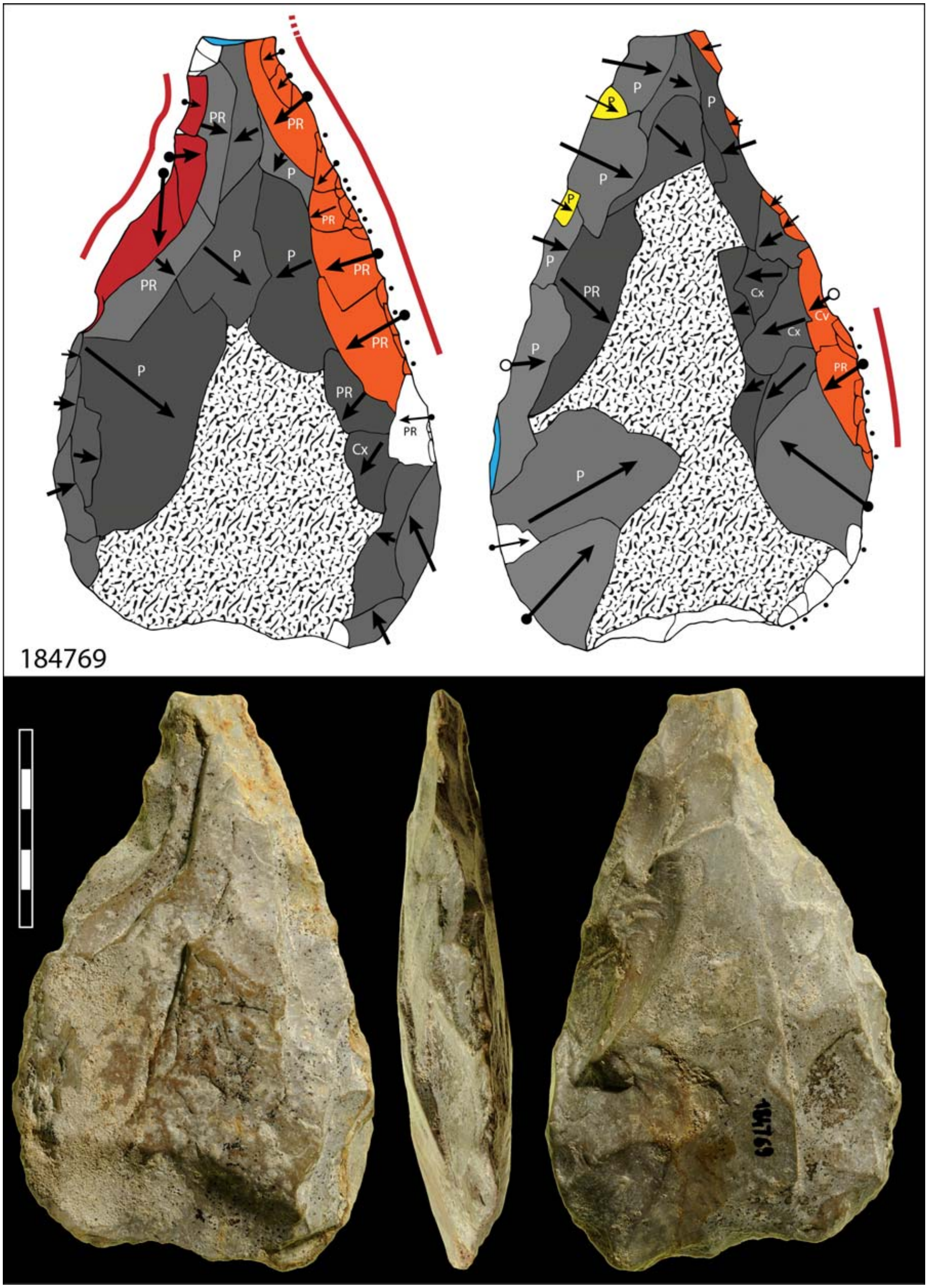


Figure P. Pigorini Museum catalogue number 184769. Slightly abraded biface of lithographic limestone, tip broken by ancient fracture. Drawing symbols as in Figure O.

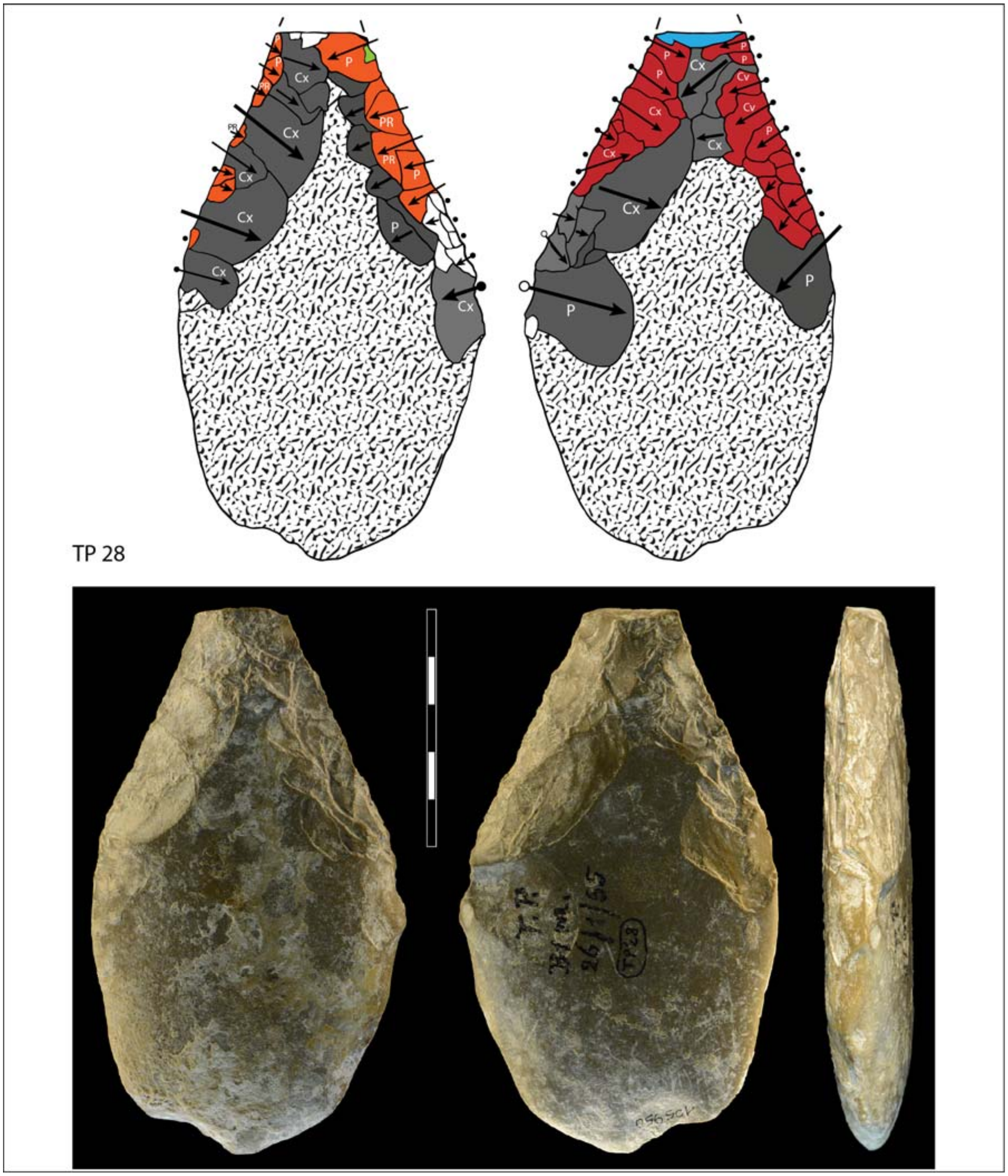


Figure Q. TP 28, slightly abraded, lithographic limestone, tip broken by ancient fracture. Drawing symbols as in Figure O.

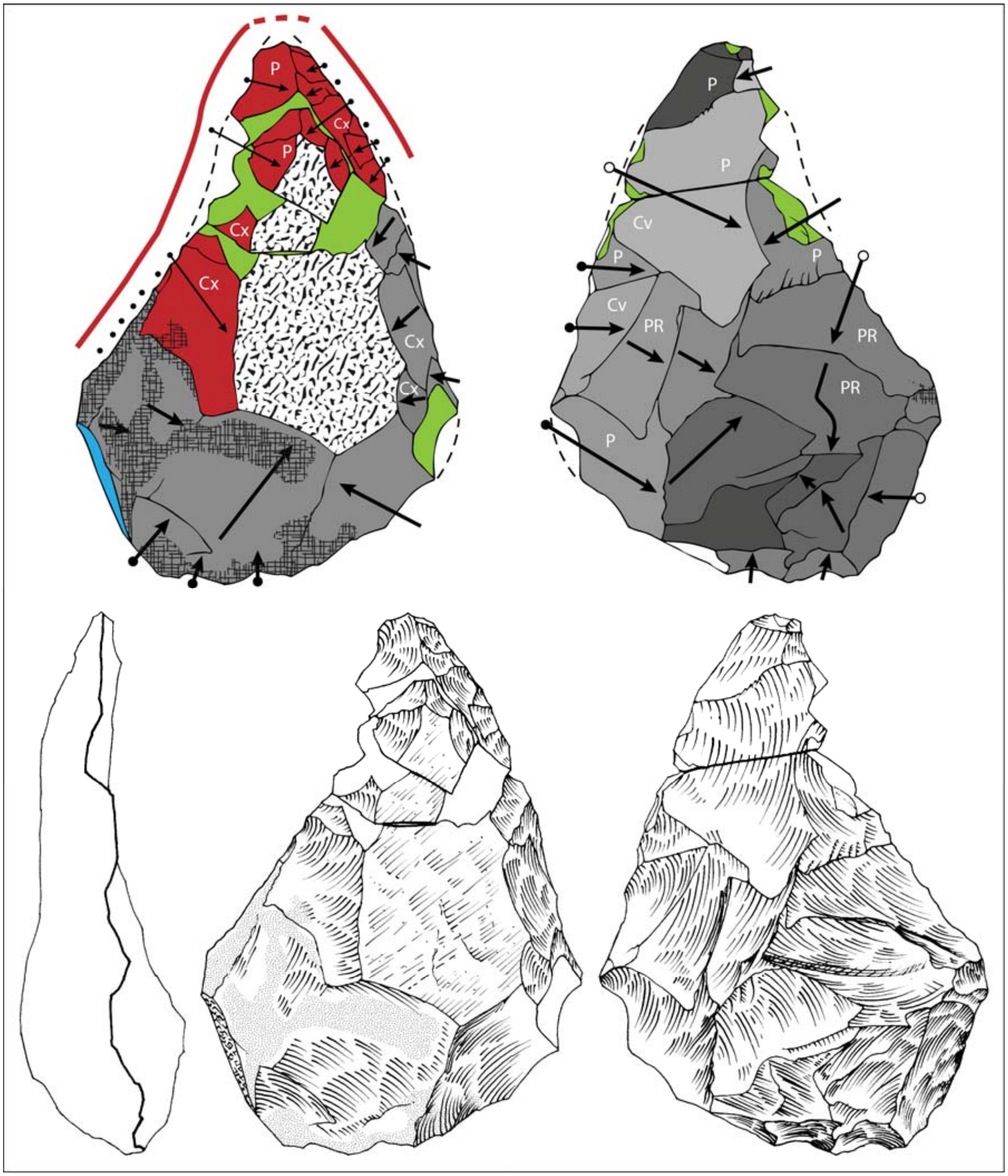


Figure R. Biface 4. 1957. Lithigraphic limestone, abraded on face 1, very slightly abraded on face 2. L = 10 cm.

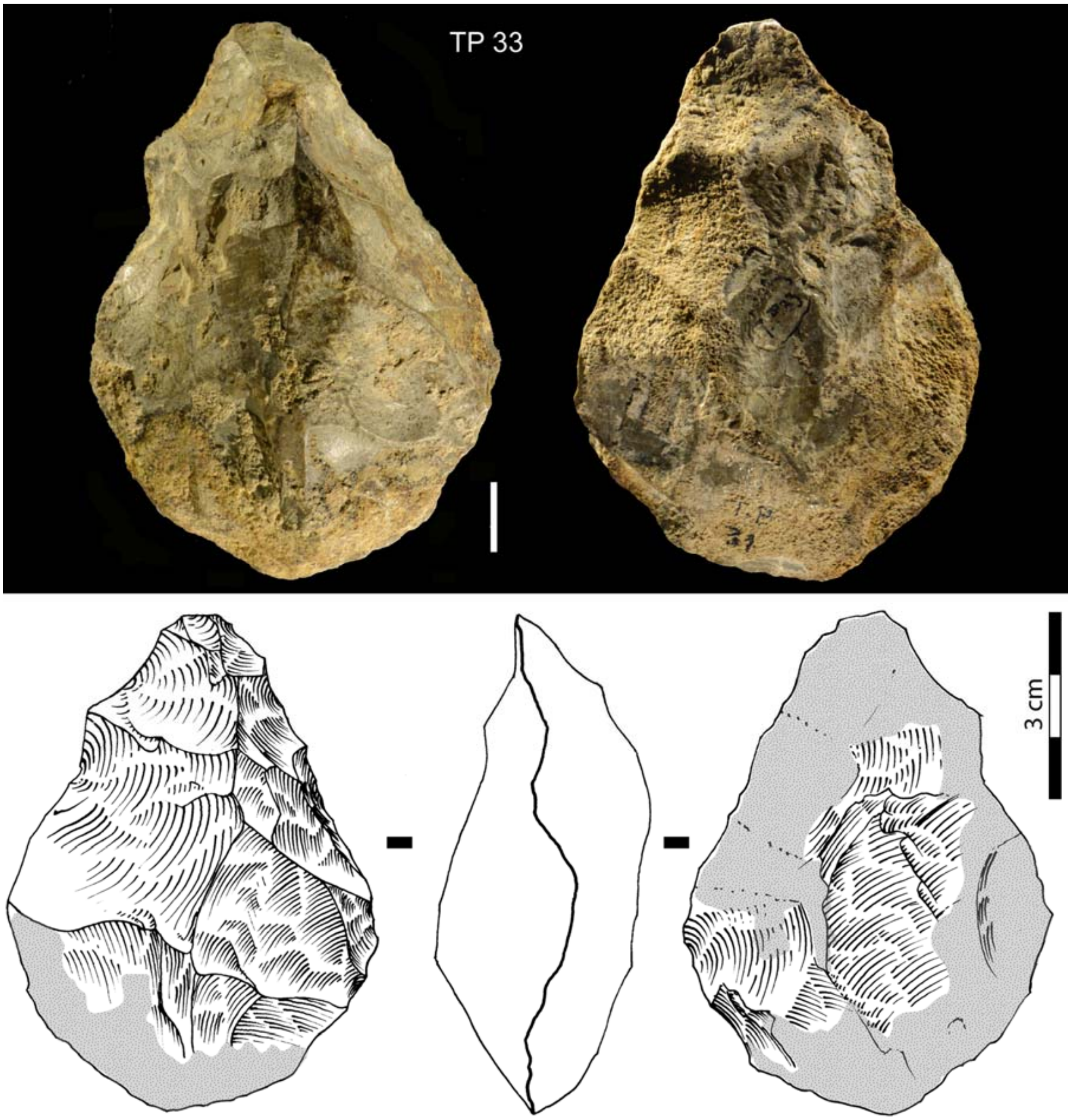


Fig S. TP 33, micritic limestone. Fresh to slightly abraded, covered with concretions. Drawing symbols as in fig O. Scale bar in photo = 1 cm.

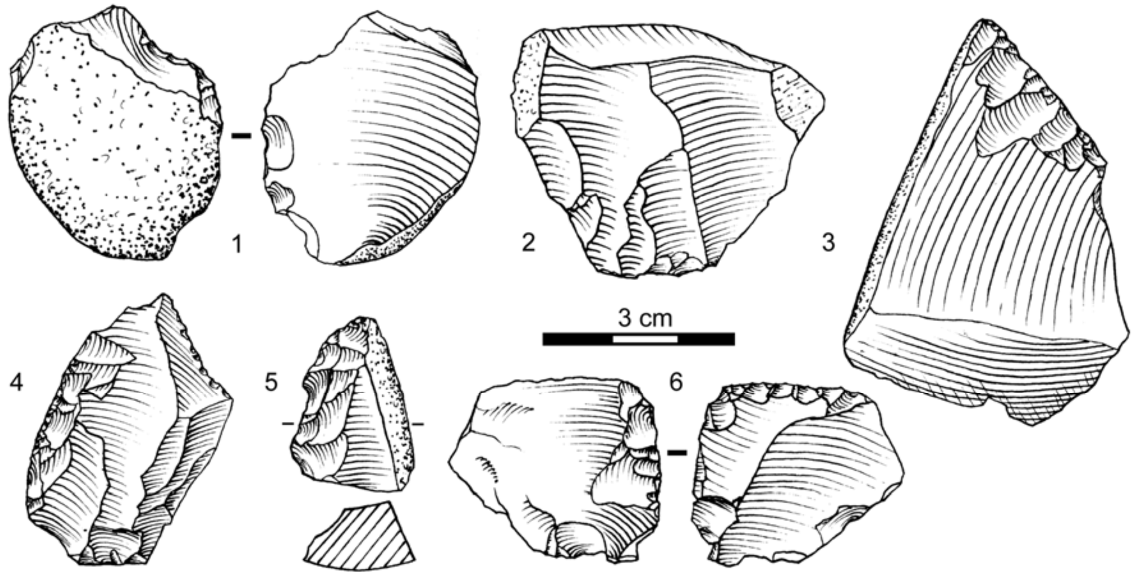


Figure T. Torre in Pietra level *m*, one shaping flake and flake tools, all flint. (1) Retouched notch on cortical flake, no. 16; (2) biface shaping flake, unretouched; (3) scraper on flake fragment, no. 5; (4) scraper on flake, no. 6; (5) scraper on a flake fragment, no. 7; (6) alternate scraper, no. 18.

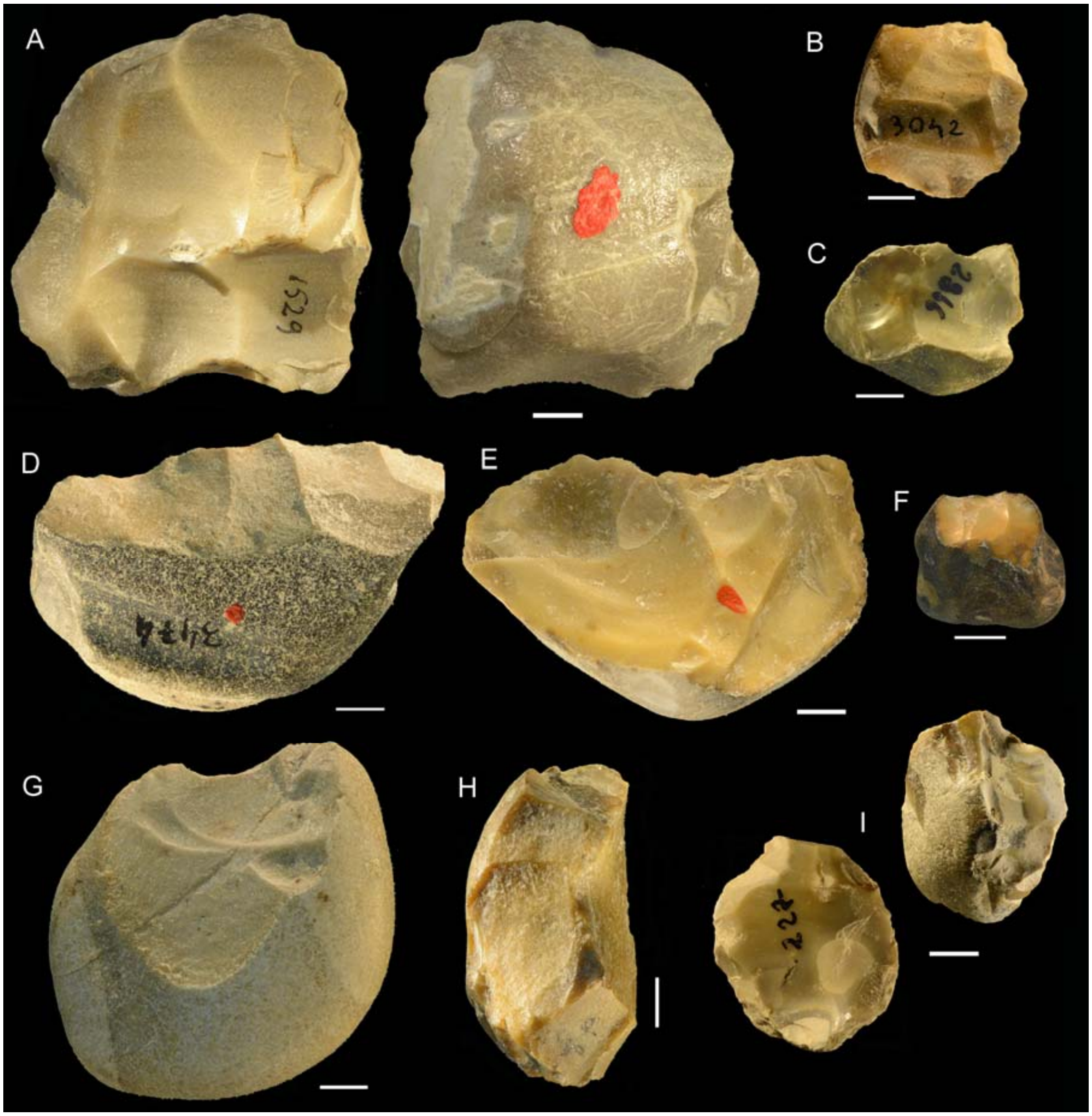


Figure U. Castel di Guido cores, all flint except D and G of chert. A, C, D - G have one debitage surface, B, H and I are bifacially flaked. Catalogue numbers: 1529, 3042, 2866, 3474, 3162, 1491, 165, 900007, 227. Scale bar = 1 cm.

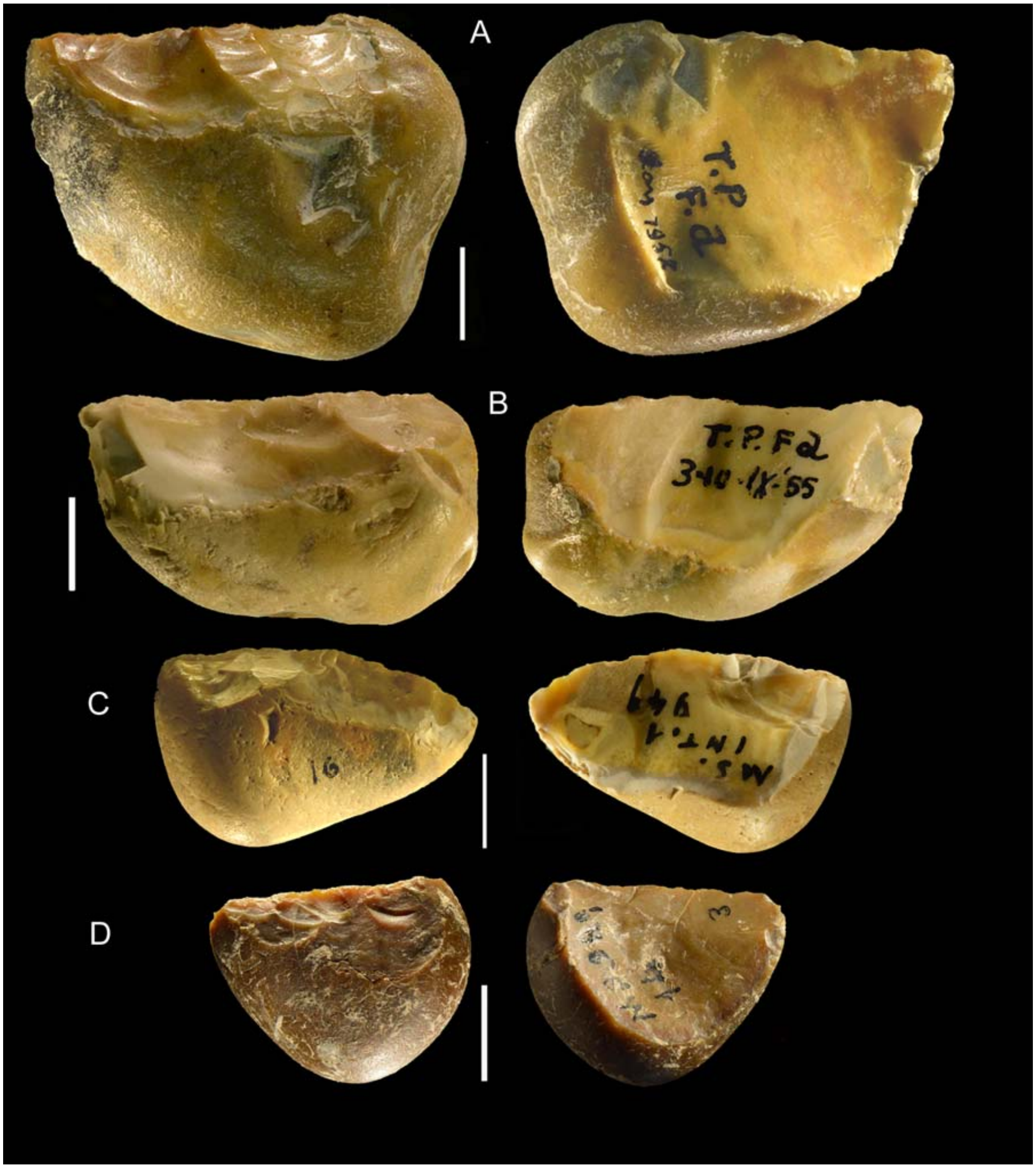


Figure W. Small tools on split pebbles or pebbles with a large removal forming the striking platform for retouch. (A, B) scrapers fromTorre in Pietra level *d*, nos. 60 and 57; (C, D) scrapers from Grotta dei Moscerini (Interno 1, no. 16 and level 14 no. 3, respectively). Scale bar = 1 cm.

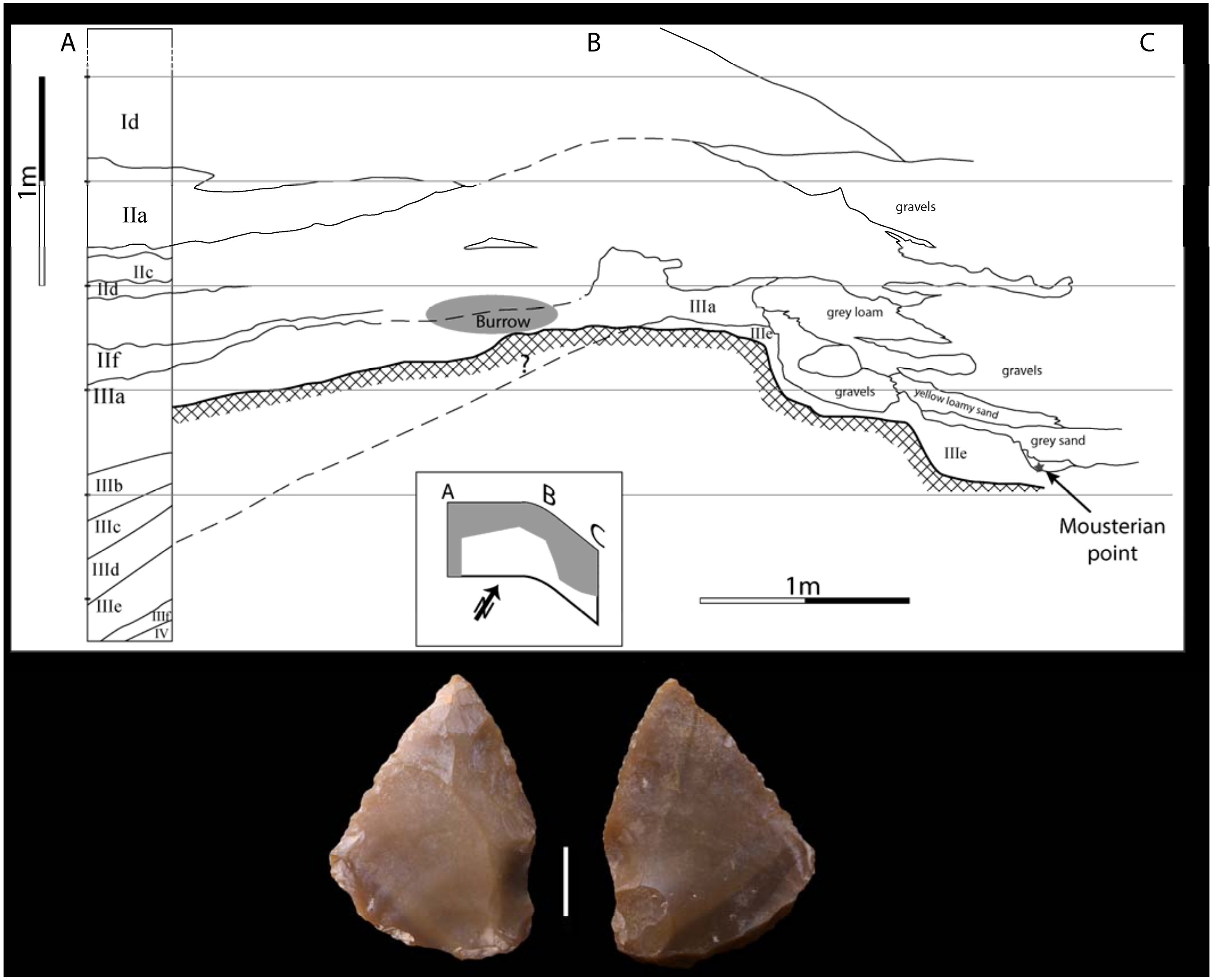


Figure X. Torre in Pietra, Vitinia deposits, lateral section (B-C) correlated to the frontal section (A, cf. text Fig. 3). The Mousterian point found at the bottom of the channel during section cleaning is in a layer above IIIe and probably equivalent to layers Iif/IIe. Scale in photo = 1 cm.

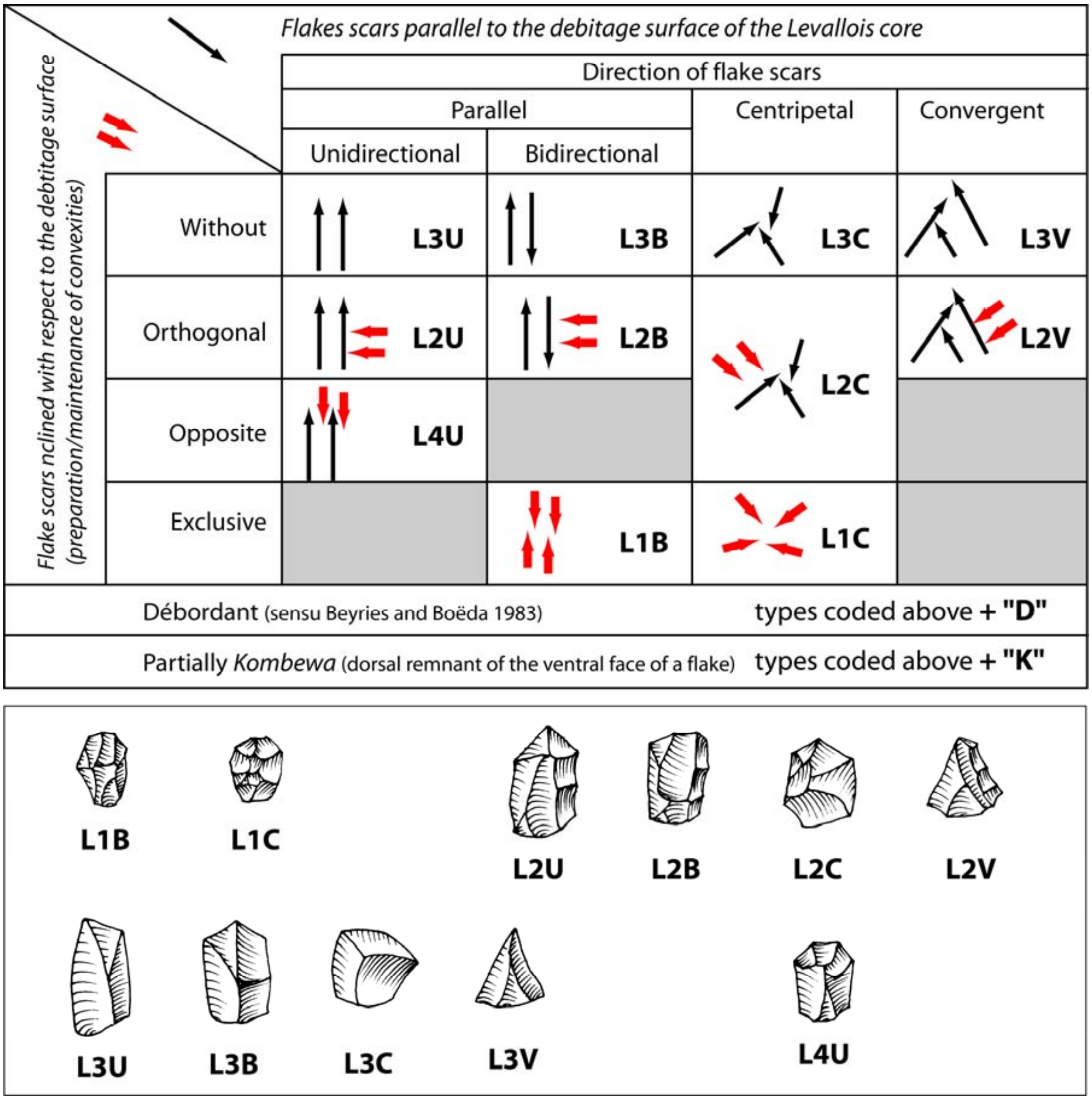


Figure Y. Torre in Pietra, level d. Coding of Levallois flake types relative to dorsal scar patterns. '*Debordant*' flakes are defined with reference to [3, 4]. Types L1, L2+L4 and L3 respectively correspond to Levallois flakes of types I, II and III from [4]. Shaded cells correspond to theoretically impossible or unlikely combination.

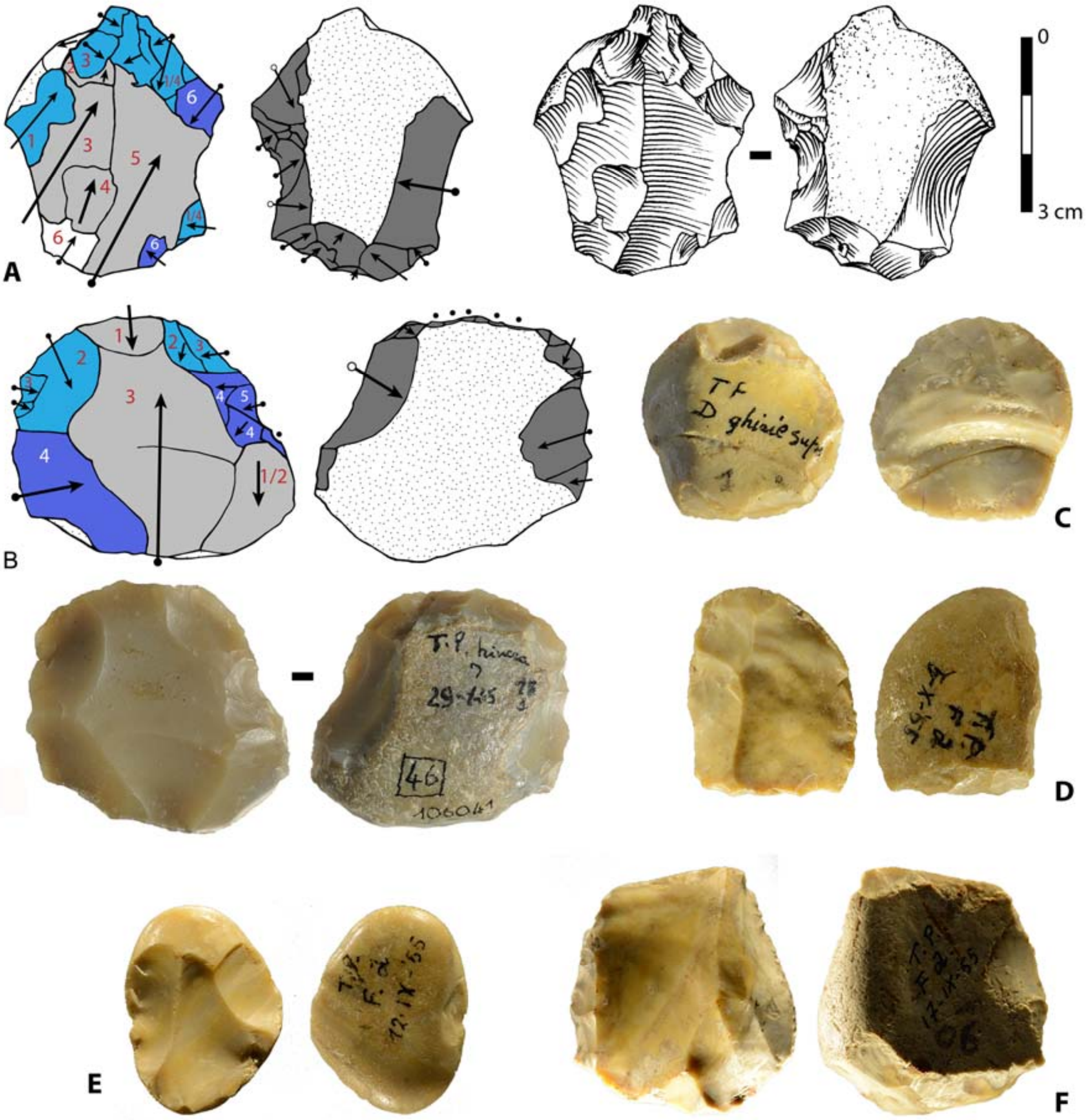


Figure Z. Torre in Pietra level *d*. A-D, F: Levallois cores; E: centripetal non-Levallois core. Catalog numbers: 19, 10641, 24, 27, 64, 17.

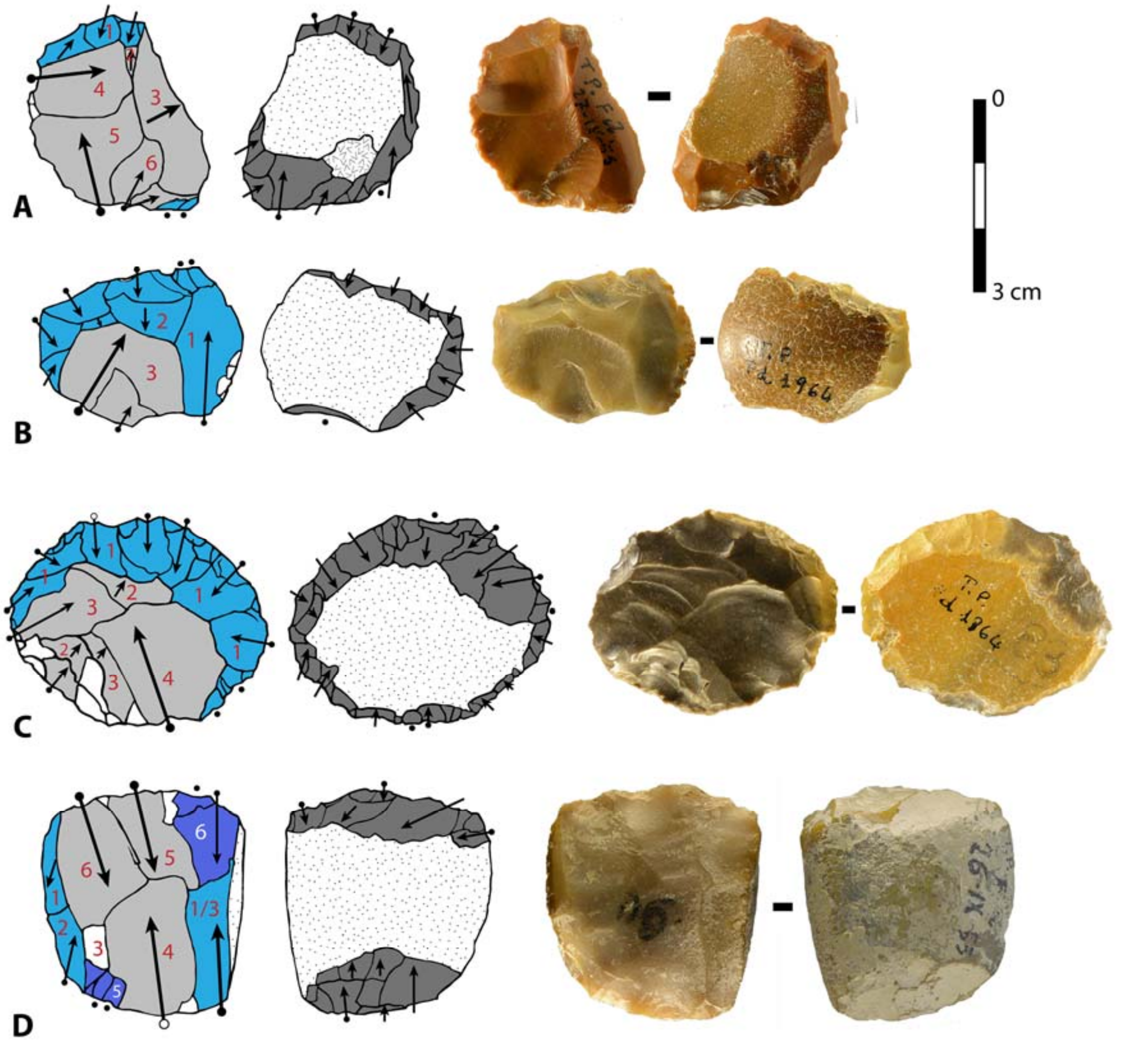


Figure AA. Torre in Pietra level *d*. A-D: Levallois cores. Catalog numbers: 61, 26, 34, 18.

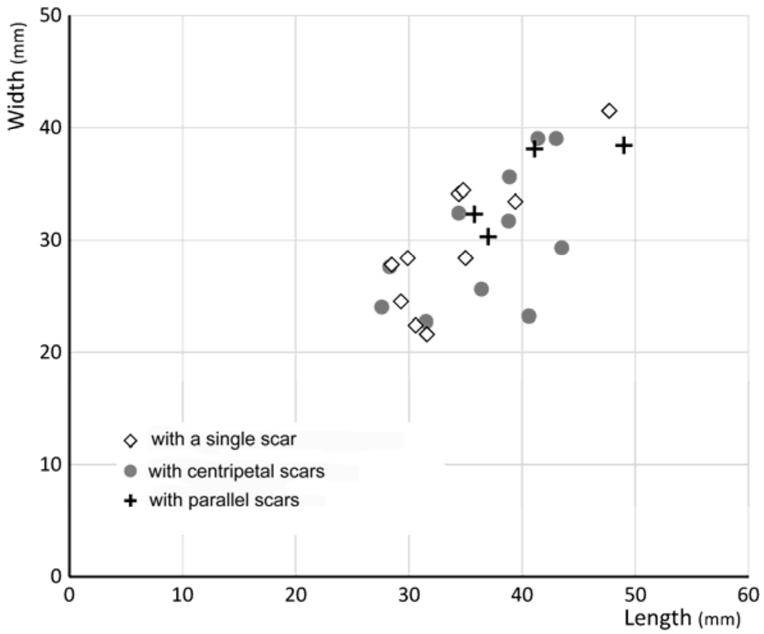


Figure AB. Torre in Pietra, level *d*. Length/width scatterplot of complete Levallois cores according to the organization of flake scars (those parallel to the plan of intersection of the surfaces of the Levallois core) on the debitage surface.

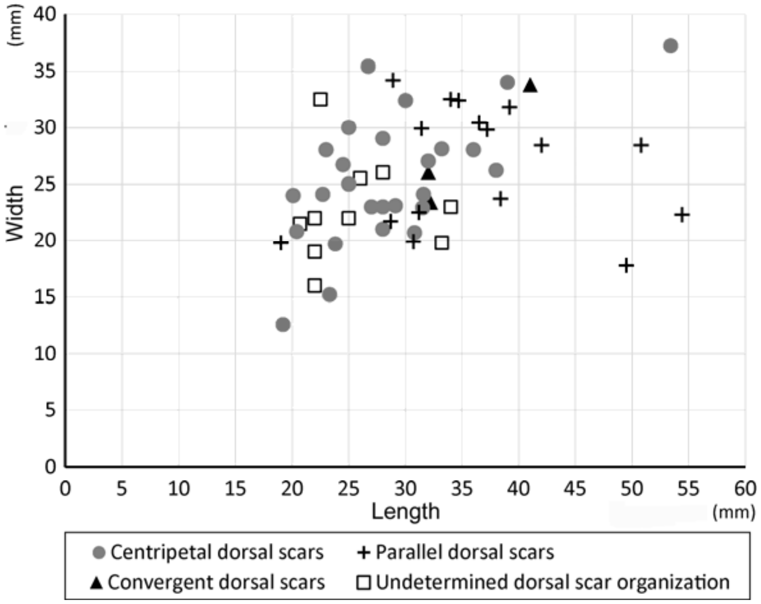


Figure AC. Torre in Pietra, level *d*. Length/width scatterplot of complete or almost complete Levallois flakes according to the organization of dorsal scars.

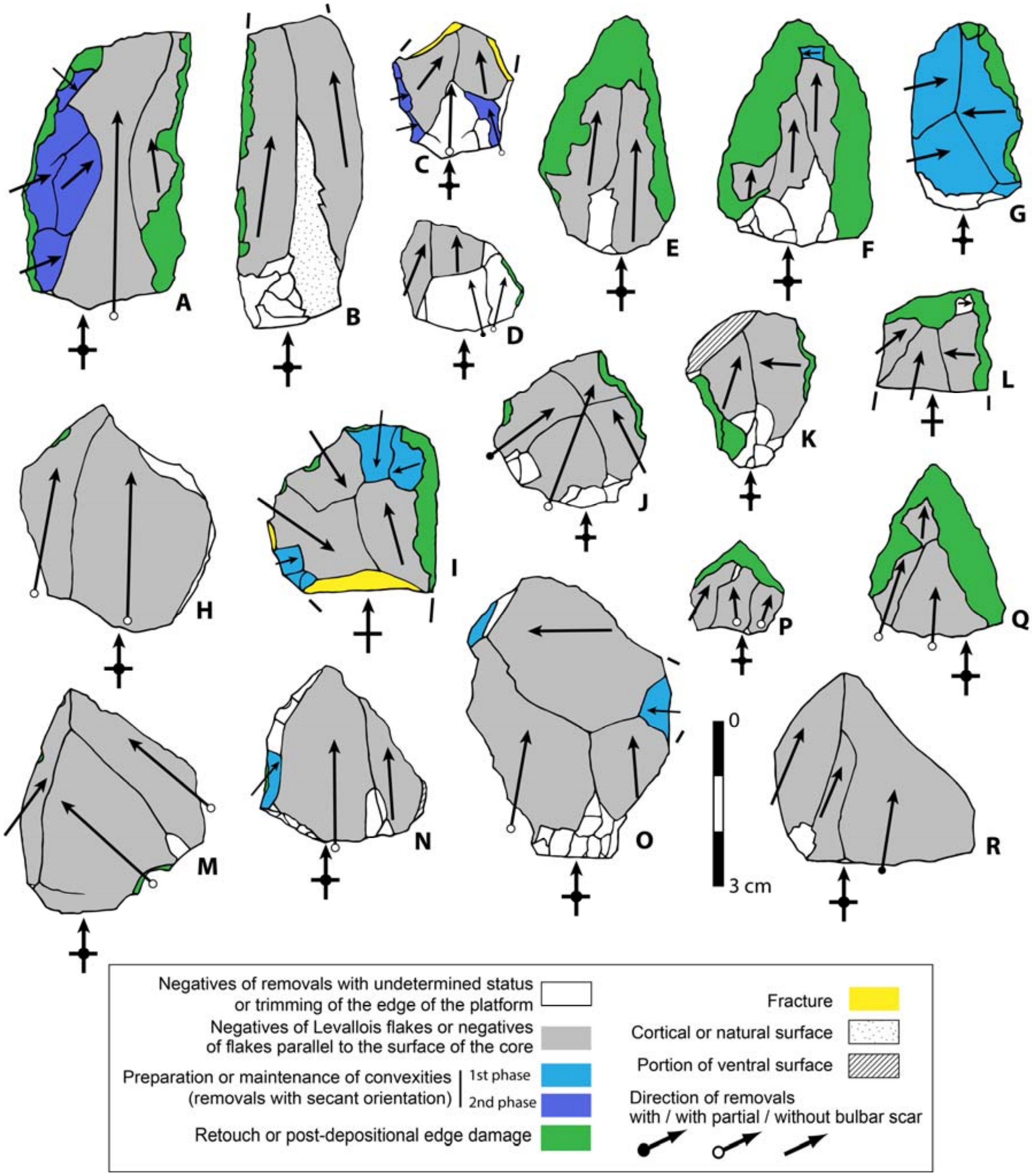


Figure AD. Torre in Pietra, level *d*. Levallois flakes.

Table A. Torre in Pietra, level *d*. Counts of Levallois flake types

| Levallois flake type | | | | |
|---|---|---|---|---|
| (a) | (b) | Unretouched | Retouched | Total |
| L1C | - | 8 | 5 | 13 |
| L2C | centripetal | 11 | 6 | 17 |
| L3C | | 5 | 2 | 7 |
| L2U | parallel | 6 | 1 | 7 |
| L2B | | 1 | 0 | 1 |
| L3U | | 7 | 12 | 19 |
| L3B | | 0 | 1 | 1 |
| L3V | convergent | 2 | 1 | 3 |
| Undet. | - | 15 | 10 | 25 |
| | Total | 55 | 38 | 93 |

(a) Coding of types as in Fig. Y

(b) Direction of dorsal flake scars parallel to the plan of intersection of the surfaces of the Levallois core

**References**

1. Malatesta A. La serie di Torre del Pagliaccetto e il bacino di Torre in Pietra. Quaternaria 1978; 20: 237-246.
2. Piperno M., Biddittu I. Studio tipologico ed interpretazione dell'industria acheuleana e premusteriana dei livelli *m* e *d* di Torre in Pietra (Rome). Quaternaria 1978; 20: 441-535.
3. Beyries S, Boëda E. Etude technologique et traces d'utilisation des "éclats débordants" de Corbehem (Pas-de-Calais). Bull Soc Préhist Fr. 1983; 80: 275–279.
4. Boëda E. Le concept Levallois: variabilité des méthodes.Paris: CNRS Editions;1994.
