# Supplementary material for: The Acheulian and Early Middle Paleolithic in Latium (Italy): Stability and Innovation
Source: PLoS One. 2016 Aug 15;11(8):e0160516. doi: 10.1371/journal.pone.0160516 (PMC4985512; doi:10.1371/journal.pone.0160516)
Supplement: S2 File — (PDF) [file pone.0160516.s002.pdf]

Supporting Information

The Acheulian and early Middle Paleolithic in Latium (Italy): Stability and Innovation

Paola Villa*, Sylvain Soriano, Rainer Grün, Fabrizio Marra, Sebastien Nomade, Alison Pereira, Giovanni Boschian, Luca Pollarolo, Fang Fang, Jean-Jacques Bahain

*To whom correspondence should be addressed. E-mail: villap@colorado

S2 File. $^{40}Ar/^{39}Ar$ Dating

This PDF file includes:

$^{40}Ar/^{39}Ar$ protocol
Tables S1-S4

$^{40}Ar/^{39}Ar$ Protocol (LSCE, Gif-sur-Yvette)

After crushing and sieving of volcanic deposits extracted from each unit, pristine sanidine crystals ranging from 500 µm up to 1 mm in size are extracted. Crystals are handpicked under a binocular microscope and slightly leached for 5 minutes in a 7 % HF acid solution in order to remove groundmass that might still be attached to them. After leaching, at least 30 crystals are handpicked for each sample and separately loaded in aluminium disks. The samples are then irradiated for 1 hour (IRR 106) in the β1 tube of the OSIRIS reactor (CEA Saclay, France). After irradiation between 15 and 20 crystals for each unit are loaded individually in a copper sample holder. The sample holder is then put into a double vacuum Cleartran window. Each sanidine is fused using a Synrad CO_2 laser at 10 to 15 % of nominal power) (c.a. 25 Watts). The extracted gas is then purified for 10 min by two hot GP 110 getters (ZrAl). Argon's isotopes (^{36}Ar, ^{37}Ar, ^{38}Ar, ^{39}Ar and ^{40}Ar) are analysed using a VG5400 mass spectrometer equipped with an electron multiplier Balzers 217 SEV SEN coupled to an ion counter. We follow the full analytical protocol outlined in detail in [1]. Neutron fluence J for each sample is calculated using co-irradiated **Alder Creek Sanidine** (ACs-2) standard with an age of **1.194 Ma** [2] and the total decay constant of [3]. J values for TIP B0 and TIP B1 are J = 0.00033260 ± 0.00000166 and J = 0.00033530 ± 0.00000168 respectively. Recent revisions of the standard and/or decay constants suggest values of about +/- 1% than the one we used. Nevertheless, the difference in the final age for levels dated is negligible well within the full-propagated uncertainties [4-7]. Procedural blank measurements are computed after every three unknown samples. For typical 9 min static blank, typical backgrounds are about 2.0-3.0 x 10^{-17} and 5.0 to 6.0 x 10^{-19} moles for ^{40}Ar and ^{36}Ar respectively. The precision and accuracy of the mass discrimination correction was monitored by weekly measurements of air argon of various beam sizes.

References

1. Nomade S, Gauthier A, Guillou H, Pastre JF. $^{40}Ar/^{39}Ar$ temporal framework for the Alleret maar lacustrine sequence (French Massif Central): Volcanological and Paleoclimatic implications. Quat Geochronol. 2010: 5, 20-27.
2. Nomade S, Renne PR, Vogel N, Deino AL, Sharp WD, Becker TA, Jaouni AR, Mundil R. Alder Creek sanidine (ACs-2), A Quaternary $^{40}Ar/^{39}Ar$ dating standard tied to the Cobb Mountain geomagnetic event. Chem Geol. 2005: 218, 315–338.
3. Steiger RH. Jäger E. Subcommission on geochronology: convention on the use of decay constants in geo- and cosmochronology. Earth Planet Sci Lett. 1977: 6, 359-362
4. Kuiper KF, Deino A, Hilgen FJ, Krijgsman W, Renne, PR, Wijbrans J.R. Synchronizing rock clocks of Earth history. Science 2008: 320, 500-504.

5. Renne PR, Mundil R, Balco G, Min K, Ludwig, KR. Response to the comment by W.H. Schwarz et al. on "Joint determination of ^{40}K decay constants and $^{40}Ar^{*}/^{40}K$ for the Fish Canyon sanidine standard, and improved accuracy for $^{40}Ar/^{39}Ar$ geochronology" by P.R. Renne et al. (2010). Geochim Cosmochim Acta. 2011: 75, 5097-5100.
6. Phillips D, Matchan EL. Ultra-high precision $^{40}Ar/^{39}Ar$ ages for Fish Canyon Tuff and Alder Creek Rhyolite sanidine: New dating standards required? Geochimica et Cosmochimica Acta 2013: 121, 229–239.
7. Rivera TA, Storey M, Schmitz MD, Crowley JL. Age intercalibration of $^{40}Ar/^{39}Ar$ sanidine and chemically distinct U/Pb zircon populations from the Alder Creek Rhyolite Quaternary geochronology standard. Chemical Geology 2013: 345, 87-98.

Sample ID:TIP-B0		Lab#N1456-01/N1456-15	J = 0.00033260 ± 0.00000166			
Sanidine			Irradiation # 106	reactor	OSIRIS	
Flux standard	ACS-2	1,194 Ma	**Single crystal total fusion**			

N	^{40}Ar (moles)	^{36}Ar V	±s_{36} V	^{37}Ar V	±s_{37} V	^{38}Ar V	±s_{38} V	^{39}Ar V	±s_{39} V	^{40}Ar V	±s_{40} V	D$^{(1)}$	±%s_D	%^{40}Ar*	Age (ka)	±s (ka)	K/Ca ± 1s
N1456-01	3.413E-15	3.142E-07	5.165E+00	3.376E-05	3.177E+00	5.143E-05	3.704E-01	3.336E-03	1.413E-01	2.491E-03	0.072	1.01295	0.1	96.35	431.7	± 1.1	14.8 ± 0.5
N1456-02	2.394E-15	5.981E-07	2.406E+00	2.233E-05	3.272E+00	3.392E-05	4.500E-01	2.176E-03	1.887E-01	1.747E-03	0.113	1.01276	0.1	89.89	433.0	± 1.5	14.6 ± 0.5
N1456-03	2.361E-15	9.968E-07	1.987E+00	2.233E-05	3.272E+00	3.627E-05	4.765E-01	2.416E-03	1.280E-01	1.723E-03	0.073	1.01275	0.1	82.83	354.5	± 1.6	16.2 ± 0.5
N1456-04	4.029E-15	1.678E-06	8.909E-01	2.803E-05	3.701E+00	4.951E-05	4.750E-01	3.093E-03	1.561E-01	2.941E-03	0.093	1.01307	0.1	83.05	473.7	± 1.3	16.6 ± 0.6
N1456-05	2.894E-15	8.151E-07	1.884E+00	2.804E-05	4.195E+00	4.266E-05	6.337E-01	2.654E-03	1.414E-01	2.113E-03	0.105	1.01286	0.1	88.61	423.2	± 1.3	14.2 ± 0.6
N1456-06	4.077E-15	1.083E-06	1.270E+00	4.519E-05	1.683E+01	5.689E-05	5.207E-01	3.546E-03	1.720E-01	2.976E-03	0.113	1.01308	0.1	89.28	449.5	± 1.2	11.8 ± 2.0
N1456-07	3.993E-15	1.042E-06	9.940E-01	3.375E-05	1.690E+01	5.016E-05	4.479E-01	3.178E-03	1.720E-01	2.915E-03	0.103	1.01306	0.1	89.43	492.0	± 1.2	14.1 ± 2.4
**N1456-08**	**2.620E-15**	**2.411E-06**	**1.183E+00**	**1.786E-05**	**8.358E+00**	**3.419E-05**	**5.414E-01**	**2.128E-03**	**1.724E-01**	**1.912E-03**	**0.105**	**1.0128**	**0.1**	**62.45**	**336.7**	**± 2.6**	**## ± 1.5**
N1456-09	1.947E-15	7.956E-07	2.575E+00	2.263E-05	1.517E+00	2.741E-05	4.049E-01	1.775E-03	1.770E-01	1.421E-03	0.150	1.01268	0.1	83.43	400.8	± 2.3	11.8 ± 0.2
N1456-10	2.330E-15	4.746E-07	4.181E+00	2.391E-05	8.211E+00	3.451E-05	6.339E-01	2.162E-03	1.754E-01	1.701E-03	0.158	1.01275	0.1	91.80	433.3	± 2.0	13.6 ± 1.1
N1456-11	2.660E-15	1.912E-07	6.005E+00	2.028E-05	1.048E+01	4.125E-05	3.702E-01	2.617E-03	1.639E-01	1.942E-03	0.095	1.01281	0.1	97.15	432.4	± 1.1	19.4 ± 2.0
N1456-12	1.530E-15	7.743E-08	2.734E+01	2.029E-05	1.048E+01	2.642E-05	8.753E-01	1.703E-03	1.886E-01	1.117E-03	0.111	1.0126	0.1	98.10	386.0	± 2.4	12.6 ± 1.3
N1456-13	1.046E-15	1.417E-07	1.237E+01	1.093E-05	3.295E+01	1.635E-05	3.875E-01	1.029E-03	1.804E-01	7.637E-04	0.224	1.0125	0.1	94.63	421.3	± 3.3	14.1 ± 4.7
N1456-14	2.216E-15	1.257E-07	1.028E+01	2.082E-05	2.615E+01	3.452E-05	5.671E-01	2.165E-03	1.720E-01	1.618E-03	0.145	1.01273	0.1	97.84	438.6	± 1.5	15.6 ± 4.1
N1456-15	7.835E-16	6.396E-08	2.156E+01	5.156E-06	1.020E+02	1.048E-05	9.062E-01	6.712E-04	4.416E-01	5.719E-04	0.485	1.01243	0.1	96.77	494.7	± 5.0	19.5 ± 19.9

Background corrections TIP-B0

N	^{36}Ar V	±s36 V	^{37}Ar V	±s37 V	^{38}Ar V	±s38 V	^{39}Ar V	±s39 V	^{40}Ar V	±s40 V
N1456-01	1.904E-07	8.701E-09	1.000E-08	3.500E-09	1.957E-08	1.671E-08	2.269E-07	1.250E-07	3.091E-05	2.844E-07
N1456-02	1.904E-07	8.701E-09	1.000E-08	3.500E-09	1.957E-08	1.671E-08	2.269E-07	1.250E-07	3.091E-05	2.844E-07
N1456-03	1.904E-07	8.701E-09	1.000E-08	3.500E-09	1.957E-08	1.671E-08	2.269E-07	1.250E-07	3.091E-05	2.844E-07
N1456-04	1.422E-07	4.949E-09	1.000E-08	3.500E-09	8.340E-08	5.921E-09	8.972E-07	9.331E-08	3.634E-05	5.814E-07
N1456-05	1.422E-07	4.949E-09	1.000E-08	3.500E-09	8.340E-08	5.921E-09	8.972E-07	9.331E-08	3.634E-05	5.814E-07
N1456-06	1.422E-07	4.949E-09	1.000E-08	3.500E-09	8.340E-08	5.921E-09	8.972E-07	9.331E-08	3.634E-05	5.814E-07
N1456-07	1.422E-07	4.949E-09	1.000E-08	3.500E-09	8.340E-08	5.921E-09	8.972E-07	9.331E-08	3.634E-05	5.814E-07
**N1456-08**	**2.271E-07**	**1.045E-08**	**1.000E-08**	**3.500E-09**	**8.460E-08**	**1.540E-08**	**1.789E-06**	**2.522E-07**	**3.070E-05**	**4.912E-07**
N1456-09	1.927E-07	5.396E-09	4.785E-09	2.771E-09	6.215E-08	8.452E-09	1.362E-06	7.505E-07	4.908E-05	5.399E-07
N1456-10	1.927E-07	5.396E-09	4.785E-08	2.771E-08	6.215E-08	8.452E-09	1.362E-06	7.505E-07	4.908E-05	5.399E-07
N1456-11	1.539E-07	8.157E-09	1.120E-08	6.160E-09	3.148E-08	1.196E-08	7.211E-08	4.975E-08	4.013E-05	4.414E-07
N1456-12	1.539E-07	8.157E-09	1.120E-08	6.160E-09	3.148E-08	1.196E-08	7.211E-08	4.975E-08	4.013E-05	4.414E-07
N1456-13	8.398E-08	1.713E-08	1.522E-08	3.500E-09	3.106E-08	2.361E-08	1.721E-07	9.121E-08	2.320E-05	4.408E-07
N1456-14	1.167E-07	1.739E-09	1.522E-08	3.500E-09	3.246E-08	4.869E-09	1.325E-07	7.023E-08	2.320E-05	4.408E-07
N1456-15	1.167E-07	1.739E-09	1.522E-08	3.500E-09	3.246E-08	4.869E-09	1.325E-07	7.023E-08	2.320E-05	4.408E-07

Table S1

Sample ID:TIP-B2	Lab#N1470-01/N471-15	J = 0.00033500 ± 0.00000335		
Sanidine		Irradiation # 106	reactor	OSIRIS
Flux standard ACS-2	1.194 Ma	**Single crystal total fusion**		

N	^{40}Ar (moles)	^{36}Ar V	±s$_{36}$ V	^{37}Ar V	±s$_{37}$ V	^{38}Ar V	±s$_{38}$ V	^{39}Ar V	±s$_{39}$ V	^{40}Ar V	±s$_{40}$ V	D$^{(1)}$	±%s$_D$	%^{40}Ar*	Age (ka)	±s (ka)	K/Ca ± 1s
**N1470-01**	**2.517E-15**	**2.322E-07**	**8.053E+00**	**2.622E-05**	**2.136E+01**	**5.196E-05**	**4.533E-01**	**2.816E-03**	**9.898E-02**	**1.837E-03**	**0.137**	**1.01278**	**0.07**	**96.46**	**387.2**	**± 1.4**	**## ± 9.9**
N1471-01	4.747E-15	2.584E-06	1.009E+00	3.200E-05	1.508E+01	6.629E-05	3.501E-01	3.810E-03	1.140E-01	3.465E-03	0.083	1.0132	0.07	77.87	435.8	± 1.4	51.2 ± 7.7
N1471-02	2.151E-15	2.613E-07	9.052E+00	2.437E-05	1.837E+01	2.920E-05	5.245E-01	1.881E-03	1.140E-01	1.570E-03	0.082	1.01271	0.07	95.21	489.0	± 2.4	33.2 ± 6.1
N1471-03	2.132E-15	7.633E-07	4.899E+00	2.319E-05	2.718E+01	2.491E-05	7.197E-01	1.743E-03	1.839E-01	1.556E-03	0.153	1.01262	0.07	85.50	469.8	± 4.1	32.3 ± 8.8
N1471-04	4.453E-15	3.959E-06	1.999E+00	3.324E-05	1.401E+01	4.434E-05	2.541E-01	2.810E-03	1.140E-01	3.250E-03	0.091	1.01315	0.07	63.75	453.7	± 5.2	36.4 ± 5.1
N1471-05	2.837E-15	1.431E-07	2.151E+01	3.327E-05	1.401E+01	3.999E-05	5.909E-01	2.222E-03	1.140E-01	2.071E-03	0.089	1.01285	0.07	98.16	563.0	± 2.7	28.7 ± 4.0
N1471-06	2.444E-15	1.777E-06	1.415E+00	1.545E-05	2.802E+01	2.334E-05	4.072E-01	1.569E-03	1.222E-01	1.784E-03	0.139	1.01277	0.07	70.35	492.2	± 3.2	43.7 ± 12.2
N1471-07	1.735E-15	6.626E-07	1.970E+00	1.308E-05	3.287E+01	2.351E-05	5.176E-01	1.318E-03	1.657E-01	1.267E-03	0.155	1.01264	0.07	84.54	499.8	± 2.2	43.3 ± 14.2
N1471-08	1.022E-15	3.121E-07	5.772E+00	9.515E-06	4.475E+01	1.782E-05	6.313E-01	9.882E-04	1.483E-01	7.459E-04	0.149	1.01252	0.07	87.70	407.4	± 3.5	44.7 ± 20.0
N1471-09	2.682E-15	5.822E-07	3.622E+00	1.845E-05	2.374E+01	3.176E-05	7.173E-01	2.080E-03	1.932E-01	1.958E-03	0.264	1.01235	0.07	91.22	528.3	± 2.6	48.5 ± 11.5
N1471-10	2.321E-15	1.454E-07	1.595E+01	2.737E-05	1.658E+01	3.549E-05	3.771E-01	2.053E-03	1.063E-01	1.694E-03	0.148	1.01235	0.07	97.65	495.9	± 2.3	32.2 ± 5.3
N1471-11	3.658E-15	3.188E-06	8.152E-01	1.547E-05	2.802E+01	3.096E-05	5.098E-01	1.988E-03	1.304E-01	2.670E-03	0.104	1.01237	0.07	64.42	532.3	± 2.7	55.3 ± 15.5
N1471-12	4.048E-15	5.463E-06	8.850E-01	2.301E-05	1.569E+01	2.660E-05	6.193E-01	1.696E-03	1.304E-01	2.955E-03	0.112	1.01307	0.07	44.89	481.5	± 5.4	31.7 ± 5.0
N1471-13	2.809E-15	3.058E-07	6.304E+00	3.212E-05	1.193E+01	3.643E-05	7.844E-01	2.570E-03	1.140E-01	2.050E-03	0.093	1.01284	0.07	95.70	469.8	± 1.6	34.4 ± 4.1
N1471-14	2.898E-15	1.586E-06	1.729E+00	3.334E-05	1.508E+01	3.231E-05	8.654E-01	1.976E-03	1.749E-01	2.116E-03	0.084	1.01277	0.07	77.81	512.7	± 2.8	25.5 ± 3.8
N1471-15	1.704E-15	1.670E-07	1.358E+01	1.820E-05	2.612E+01	2.116E-05	6.948E-01	1.404E-03	1.146E-01	1.244E-03	0.109	1.01255	0.07	96.15	524.1	± 3.1	33.2 ± 8.7

Background corrections TIP-B2

N	^{36}Ar V	±s36 V	^{37}Ar V	±s37 V	^{38}Ar V	±s38 V	^{39}Ar V	±s39 V	^{40}Ar V	±s40 V
N1470-01	1.259E-07	1.335E-08	1.500E-08	7.500E-09	6.513E-08	1.277E-08	3.665E-07	1.001E-07	3.086E-05	6.789E-07
N1471-01	1.259E-07	1.335E-08	1.500E-08	7.500E-09	6.513E-08	1.277E-08	3.665E-07	1.001E-07	3.086E-05	6.789E-07
N1471-02	1.252E-07	1.903E-08	1.400E-08	7.000E-09	5.444E-08	2.025E-08	1.672E-07	6.956E-08	2.677E-05	2.142E-07
N1471-03	1.252E-07	1.903E-08	1.400E-08	7.000E-09	5.444E-08	2.025E-08	1.672E-07	6.956E-08	2.677E-05	2.142E-07
N1471-04	2.048E-07	5.898E-08	1.400E-08	7.000E-09	1.310E-08	1.309E-08	1.672E-07	6.956E-08	2.680E-05	3.618E-07
N1471-05	2.800E-07	1.840E-08	1.400E-08	7.000E-09	1.216E-07	2.128E-08	1.026E-06	9.183E-08	3.941E-05	7.213E-07
N1471-06	2.800E-07	1.840E-08	1.400E-08	7.000E-09	1.216E-07	2.128E-08	1.026E-06	9.183E-08	3.941E-05	7.213E-07
N1471-07	3.323E-07	9.105E-09	1.400E-08	7.000E-09	1.216E-07	1.836E-08	1.026E-06	9.183E-08	3.941E-05	7.213E-07
N1471-08	3.323E-07	9.105E-09	1.400E-08	7.000E-09	1.402E-07	2.117E-08	1.712E-06	1.185E-07	1.120E-04	5.824E-07
N1471-09	4.195E-07	9.355E-09	1.400E-08	7.000E-09	9.192E-08	1.489E-08	8.159E-07	8.893E-08	9.430E-05	1.547E-06
N1471-10	1.629E-07	1.645E-08	1.400E-08	7.000E-09	3.785E-09	3.781E-09	1.647E-06	6.209E-08	5.116E-05	5.883E-07
N1471-11	1.629E-07	1.645E-08	1.400E-08	7.000E-09	3.785E-09	3.781E-09	1.647E-06	6.209E-08	5.116E-05	5.883E-07
N1471-12	1.204E-07	1.120E-08	1.200E-08	5.400E-09	3.514E-08	1.497E-08	1.149E-07	5.435E-08	2.791E-05	3.349E-07
N1471-13	1.204E-07	1.120E-08	1.200E-08	5.400E-09	3.514E-08	1.497E-08	1.149E-07	5.435E-08	2.791E-05	3.349E-07
N1471-14	1.477E-07	1.831E-08	1.500E-08	7.500E-09	3.075E-08	2.466E-08	5.829E-07	1.766E-07	3.141E-05	4.649E-07
N1471-15	1.477E-07	1.831E-08	1.500E-08	7.500E-09	3.075E-08	2.466E-08	5.829E-07	1.766E-07	3.141E-05	4.649E-07

Table S2

Sample ID:TIP-B1 Lab#N1458-01/N458-12 J = 0.00033530 ± 0.00000168

Sanidine Irradiation # 106 reactor OSIRIS

Flux standard ACS-2 1.194 Ma **Single crystal total fusion**

N	^{40}Ar (moles)	^{36}Ar V	±s_{36} V	^{37}Ar V	±s_{37} V	^{38}Ar V	±s_{38} V	^{39}Ar V	±s_{39} V	^{40}Ar V	±s_{40} V	D(1)	±%s_D	%^{40}Ar*	Age (ka)	±s (ka)	K/Ca ± 1s
N1458-01	2.408E-15	0.0000013	0.942	0.0000199	10.351	0.0000350	0.923	0.0022171	0.172	0.0017575	0.124	1.01156	0.1	77.83	373.2	± 1.4	19.0 ± 2.0
N1458-02	1.419E-14	0.0000268	0.643	0.0000110	10.618	0.0000605	4.008	0.0035828	0.224	0.0103583	0.130	1.01389	0.1	22.76	397.9	± 10.2	55.4 ± 5.9
N1458-03	3.868E-15	0.0000061	0.520	0.0000179	15.590	0.0000257	0.469	0.0016112	0.149	0.0028235	0.135	1.01187	0.1	35.31	374.2	± 4.6	15.3 ± 2.4
N1458-04	2.440E-15	0.0000001	12.602	0.0000117	16.551	0.0000570	0.439	0.0036905	0.149	0.0017808	0.116	1.01157	0.1	97.59	284.8	± 1.1	53.7 ± 8.9
N1458-05	2.470E-15	0.0000005	4.018	0.0000272	10.463	0.0000383	0.307	0.0023569	0.172	0.0018028	0.106	1.01158	0.1	92.42	427.6	± 1.7	14.7 ± 1.5
N1458-06	4.435E-15	0.0000018	4.480	0.0000117	11.509	0.0000512	0.415	0.0030848	0.128	0.0032369	0.054	1.01196	0.1	83.72	531.3	± 4.7	44.9 ± 5.2
N1458-07	3.110E-15	0.0000002	48.361	0.0000117	11.509	0.0000468	0.523	0.0029951	0.134	0.0022699	0.058	1.0117	0.1	98.01	449.3	± 4.5	43.6 ± 5.0
**N1458-08**	**1.514E-15**	**0.0000003**	**6.139**	**0.0000117**	**11.509**	**0.0000441**	**0.709**	**0.0029712**	**0.206**	**0.0011051**	**0.119**	**1.01138**	**0.1**	**91.73**	**206.4**	**± 1.3**	**## ± 5.0**
N1458-09	2.247E-15	0.0000007	3.732	0.0000117	11.509	0.0000300	0.361	0.0019222	0.156	0.0016401	0.095	1.01153	0.1	87.07	449.4	± 2.7	27.9 ± 3.2
**N1458-10**	**1.561E-15**	**0.0000001**	**7.835**	**0.0000117**	**11.509**	**0.0000510**	**0.403**	**0.0032296**	**0.172**	**0.0011394**	**0.149**	**1.01139**	**0.1**	**97.06**	**207.1**	**± 0.7**	**## ± 5.4**
**N1458-11**	**1.818E-15**	**0.0000006**	**3.010**	**0.0000117**	**11.509**	**0.0000495**	**0.539**	**0.0032908**	**0.180**	**0.0013271**	**0.137**	**1.01144**	**0.1**	**85.79**	**209.3**	**± 1.2**	**## ± 5.5**
N1458-12	1.678E-15	0.0000003	6.116	0.0000117	11.509	0.0000212	0.718	0.0013541	0.206	0.0012251	0.239	1.01142	0.1	91.56	501.1	± 3.3	19.7 ± 2.3
N1458-13	1.408E-15	0.0000003	7.127	0.0000145	17.239	0.0000224	6.512	0.0014530	0.260	0.0010278	0.269	1.01257	0.1	92.37	395.2	± 2.8	17.1 ± 2.9
**N1458-14**	**2.087E-15**	**0.0000037**	**0.970**	**0.0000057**	**29.693**	**0.0000179**	**6.703**	**0.0011825**	**0.224**	**0.0015233**	**0.118**	**1.0127**	**0.1**	**27.59**	**215.0**	**± 5.9**	**## ± 10.4**
**N1458-15**	**8.279E-16**	**0.0000002**	**8.434**	**0.0000069**	**26.144**	**0.0000239**	**0.529**	**0.0016055**	**0.215**	**0.0006043**	**0.249**	**1.01246**	**0.1**	**91.67**	**208.7**	**± 1.8**	**## ± 10.3**

Results	40Ar*/39Ark ± 1s	Age ± 1s (Ka)	MSWD	39Ar(k) (n)	K/Ca ± 1s
**Weighted mean**	**0.3432** **± 0.0010** **± 0.29%**	**207.6** **± 1.2** **± 0.50%**	**1.35**	**33.60** **5**	**44.5 ± 2.8**
		**Full External Error ± 2.8**	**1.06**	Statistical T ratio	
		**Analytical Error ± 0.6**			

**Results**	40(a)/36(a) ± 1s	40(r)/39(k) ± 1s	Age ± 1s (Ka)	**MSWD**
**Inverse Isochron**	**303.4325** **± 3.0215** **± 1.00%**	**0.3427** **± 0.0009** **± 0.27%**	**207.3** **± 1.2** **± 0.57%**	**0.99**
**Full External Error**			**± 2.8**	
**Analytical Error**			**± 0.6**	

**Statistics**				
	Statistical F ratio	**1.09**	Convergence	**0.0073708120**
	Error Magnification	**1.0000**	Number of Iterations	**2**
	Number of Data Points	**5**	Calculated Line	**Weighted York-2**

Table S3

Background corrections TIP-B1

N	^{36}Ar V	±s36 V	^{37}Ar V	±s37 V	^{38}Ar V	±s38 V	^{39}Ar V	±s39 V	^{40}Ar V	±s40 V
N1458-01	1.538E-07	4.799E-09	1.100E-08	1.100E-09	3.399E-08	1.132E-08	5.776E-07	9.068E-08	3.249E-05	3.931E-07
N1458-02	1.513E-07	8.775E-09	1.100E-08	1.100E-09	4.179E-08	1.178E-08	8.777E-07	6.407E-08	3.169E-05	4.405E-07
N1458-03	2.452E-07	1.152E-08	1.100E-08	1.100E-09	6.390E-08	1.336E-08	1.000E-07	7.400E-08	7.052E-05	5.642E-07
N1458-04	2.178E-07	1.568E-08	1.200E-08	8.160E-09	1.120E-07	1.142E-08	2.372E-06	1.566E-07	4.825E-05	4.922E-07
N1458-05	2.178E-07	1.568E-08	1.200E-08	8.160E-09	1.120E-07	1.142E-08	2.372E-06	1.566E-07	4.825E-05	4.922E-07
N1458-06	2.335E-07	7.659E-08	1.200E-08	8.160E-09	1.118E-07	2.191E-08	1.037E-06	1.141E-07	4.613E-05	6.135E-07
N1458-07	2.335E-07	7.659E-08	1.200E-08	8.160E-09	1.118E-07	2.191E-08	1.037E-06	1.141E-07	4.613E-05	6.135E-07
**N1458-08**	**1.781E-07**	**6.964E-09**	**1.200E-08**	**8.160E-09**	**1.069E-09**	**1.068E-09**	**5.191E-07**	**9.500E-08**	**3.387E-05**	**4.064E-07**
N1458-09	1.781E-07	6.964E-09	1.200E-08	8.160E-09	1.069E-09	1.068E-09	5.191E-07	9.500E-08	3.387E-05	4.064E-07
**N1458-10**	**1.781E-07**	**6.964E-09**	**1.200E-08**	**8.160E-09**	**1.069E-09**	**1.068E-09**	**5.191E-07**	**9.500E-08**	**3.387E-05**	**4.064E-07**
**N1458-11**	**1.781E-07**	**6.964E-09**	**1.200E-08**	**8.160E-09**	**1.069E-09**	**1.068E-09**	**5.191E-07**	**9.500E-08**	**3.387E-05**	**4.064E-07**
N1458-12	1.781E-07	6.964E-09	1.200E-08	8.160E-09	1.069E-09	1.068E-09	5.191E-07	9.500E-08	3.387E-05	4.064E-07
N1458-13	1.167E-07	1.727E-08	1.515E-08	1.515E-09	3.246E-08	4.869E-09	1.325E-07	7.023E-08	2.321E-05	4.410E-07
**N1458-14**	**6.467E-08**	**8.924E-09**	**1.515E-08**	**1.515E-09**	**1.218E-09**	**1.217E-09**	**2.700E-07**	**6.831E-08**	**2.466E-05**	**5.918E-07**
**N1458-15**	**6.467E-08**	**8.924E-09**	**1.515E-08**	**1.515E-09**	**1.218E-09**	**1.217E-09**	**2.700E-07**	**6.831E-08**	**2.466E-05**	**5.918E-07**

Sample ID: Tor 1 Lab# N1349-01/N1349-10 J = 0.00038160 ± 0.00000076

Sanidine Irradiation # 85 reactor OSIRIS

Flux standard ACS-2 1.193 Ma Single crystal total fusion

N	^{40}Ar (moles)	^{36}Ar V	$±s_{36}$ V	^{37}Ar V	$±s_{37}$ V	^{38}Ar V	$±s_{38}$ V	^{39}Ar V	$±s_{39}$ V	^{40}Ar V	$±s_{40}$ V	D(1)	$±\%s_D$	$\%^{40}Ar^*$	Age ±s (ka) (ka)	K/Ca ± 1σ
**N1349-01**	**2.141E-15**	**5.2530E-07**	**1.8386E-08**	**7.8610E-06**	**5.5027E-08**	**4.5930E-05**	**1.5157E-07**	**2.9160E-03**	**2.6244E-06**	**1.5720E-03**	**1.8864E-06**	**1.01219**	**0.07**	**94.52**	**352.7 ± 2,4**	**#### ± 0,0429**
N1349-02	3.159E-15	3.6170E-07	2.0255E-08	6.0928E-06	7.3114E-08	5.4450E-05	2.2869E-07	3.5250E-03	2.1150E-06	2.3150E-03	1.6205E-06	1.01231	0.07	98.19	447.2 ± 2,0	#### ± 0,1049
N1349-03	2.370E-15	4.9310E-07	2.1203E-08	3.6140E-06	5.4210E-08	3.9820E-05	1.4335E-07	2.5550E-03	2.5550E-06	1.7390E-03	1.2173E-06	1.01222	0.07	95.28	449.2 ± 2,8	#### ± 0,1620
**N1349-04**	**2.835E-15**	**1.0460E-06**	**3.7656E-08**	**6.2970E-06**	**6.9267E-08**	**5.6890E-05**	**1.5360E-07**	**3.6190E-03**	**3.2571E-06**	**2.0781E-03**	**1.8703E-06**	**1.01227**	**0.07**	**88.34**	**351.7 ± 2,3**	**#### ± 0,0962**
N1349-05	6.165E-15	7.3110E-07	2.7782E-08	9.4811E-06	5.6887E-08	1.0442E-04	1.8796E-07	6.7450E-03	6.7450E-06	4.5086E-03	3.1560E-06	1.01264	0.07	96.69	449.3 ± 1,1	#### ± 0,0735
N1349-06	3.906E-15	5.9540E-07	3.6915E-08	6.4070E-06	7.6884E-08	6.5700E-05	1.9710E-07	4.2270E-03	3.3816E-06	2.8600E-03	2.5740E-06	1.01239	0.07	95.99	450.9 ± 2,0	#### ± 0,1191
N1349-07	8.205E-15	1.2690E-06	2.5380E-08	1.4165E-05	1.1474E-07	1.3630E-04	2.3171E-07	8.7100E-03	2.6130E-06	5.9975E-03	3.5985E-06	1.01287	0.07	95.08	455.4 ± 0,8	#### ± 0,0778
**N1349-08**	**2.632E-15**	**1.8776E-06**	**1.1266E-08**	**4.7550E-06**	**5.7060E-08**	**4.2620E-05**	**1.5769E-07**	**2.7955E-03**	**2.2364E-06**	**1.9290E-03**	**1.9290E-06**	**1.01225**	**0.07**	**74.35**	**355.8 ± 1,7**	**#### ± 0,1086**
N1349-09	6.171E-15	8.7779E-07	2.6334E-08	9.8657E-06	6.4127E-08	1.0414E-04	1.5621E-07	6.6290E-03	3.3145E-06	4.5118E-03	2.7071E-06	1.01264	0.07	95.44	451.7 ± 1,1	#### ± 0,0733
N1349-10	5.113E-15	1.2210E-06	2.4420E-08	9.0900E-06	8.1810E-08	8.3860E-05	2.0126E-07	5.2610E-03	5.2610E-06	3.7396E-03	4.4875E-06	1.01287	0.07	91.90	454.2 ± 1,4	#### ± 0,0812

Results	40Ar*/39Ark ± 1σ	Age ± 1σ (Ka)	MSWD	39Ar(k) (n%)	K/Ca ± 1σ
Weighted mean	0.5142<br>± 0,0080<br>± 1,60%	354.0 ± 2,3	1.20	0.19<br>3	5.9 ± 0,1

Background corrections Tor 1										
N	Ar	$\pm s_{36}$	Ar	$\pm s_{37}$	Ar	$\pm s_{38}$	Ar	$\pm s_{39}$	Ar	$\pm s_{40}$
	V	V	V	V	V	V	V	V	V	V
**N1349-01**	**1.8960E-07**	**2.8440E-08**	**1.0715E-07**	**8.7863E-09**	**6.1180E-08**	**2.7286E-08**	**2.8200E-07**	**1.4974E-07**	**9.0523E-06**	**2.6252E-07**
N1349-02	1.8960E-07	2.8440E-08	1.0715E-07	8.7863E-09	6.1180E-08	2.7286E-08	2.8200E-07	1.4974E-07	9.0523E-06	2.6252E-07
N1349-03	1.8960E-07	2.8440E-08	1.0715E-07	8.7863E-09	6.1180E-08	2.7286E-08	2.8200E-07	1.4974E-07	9.0523E-06	2.6252E-07
**N1349-04**	**1.6396E-07**	**1.6396E-08**	**9.7370E-08**	**8.7633E-09**	**3.3190E-08**	**1.6263E-08**	**5.8690E-07**	**1.7079E-07**	**8.7660E-06**	**2.3668E-07**
N1349-05	1.6396E-07	1.6396E-08	9.7370E-08	8.7633E-09	3.3190E-08	1.6263E-08	5.8690E-07	1.7079E-07	8.7660E-06	2.3668E-07
N1349-06	1.6396E-07	1.6396E-08	9.7370E-08	8.7633E-09	3.3190E-08	1.6263E-08	5.8690E-07	1.7079E-07	8.7660E-06	2.3668E-07
N1349-07	1.6396E-07	1.6396E-08	9.7370E-08	8.7633E-09	3.3190E-08	1.6263E-08	5.8690E-07	1.7079E-07	8.7660E-06	2.3668E-07
**N1349-08**	**1.0830E-07**	**1.8411E-08**	**7.5430E-08**	**1.4407E-08**	**1.5530E-08**	**1.5375E-08**	**1.6430E-07**	**7.2456E-08**	**7.4760E-06**	**2.5418E-07**
N1349-09	1.0830E-07	1.8411E-08	7.5430E-08	1.4407E-08	1.5530E-08	1.5375E-08	1.6430E-07	7.2456E-08	7.4760E-06	2.5418E-07
N1349-10	1.0830E-07	1.8411E-08	7.5430E-08	1.4407E-08	1.5530E-08	1.5375E-08	1.6430E-07	7.2456E-08	7.4760E-06	2.5418E-07

Table S4
