# Supplementary material for: The Acheulian and Early Middle Paleolithic in Latium (Italy): Stability and Innovation
Source: PLoS One. 2016 Aug 15;11(8):e0160516. doi: 10.1371/journal.pone.0160516 (PMC4985512; doi:10.1371/journal.pone.0160516)
Supplement: S3 File — (PDF) [file pone.0160516.s003.pdf]

Supporting Information

The Acheulian and early Middle Paleolithic in Latium (Italy): Stability and Innovation

Paola Villa*, Sylvain Soriano, Rainer Grün, Fabrizio Marra, Sebastien Nomade, Alison Pereira, Giovanni Boschian, Luca Pollarolo, Fang Fang, Jean-Jacques Bahain

*To whom correspondence should be addressed. E-mail: villap@colorado

S3 File. U-series and ESR dating

This PDF file includes:

U-series and ESR protocol
Tables A-C

U-series and ESR dating protocol

U-series analysis

Laser ablation U-series analyses were carried out at the Research School of Earth Sciences (RSES) of the Australian National University, using a custom-built laser sampling system interfaced between an ArF Excimer laser and a MC-ICP-MS Finnigan Neptune [1,2] following principles and procedures described in [3].The teeth were analysed along lines perpendicular to the enamel lamella, sampling all dental tissues present. The bone was analysed along a line perpendicular to the outer surface. No individual age calculation was carried when the U-concentrations were below about 0.5 ppm and/or detrital ^{232}Th was observed (elemental U/Th ratios below 300). For the teeth, the analytical data of the enamel and dentine sections were integrated to provide the data input for the ESR age calculations. The results are shown in Table B. All isotopic ratios are expressed as activity ratio. All errors are 2-σ, including the calculated ages.

ESR analysis

The fossil teeth were prepared following a standard ESR dating procedure for enamel powder [4] : the enamel layer was mechanically separated from the other dental tissues and both inner and outer surfaces were removed with a dentist drill to eliminate the volume that received an external alpha dose. The samples were then ground and sieved to recover the size fraction of <200 µm. Dose evaluation utilized the multiple aliquot additive dose method. The powder was split into 9 aliquots, and irradiated to: 0, 7.05, 14.1, 28.2, 56.4, 113, 226, 451, 902, and 1805 Gy. ESR measurements were carried out with a Bruker Elexsys 500 spectrometer at RSES using the following acquisition parameters: 3–10 scans, 2 mW microwave power, 1024 points resolution, 12 mT sweep width, 50 kHz modulation frequency, 0.1 mT modulation amplitude, 20 ms conversion time and 5 ms time constant. The ESR intensities were extracted from T1-B2 peak-to-peak amplitudes of the ESR signal [5] and then normalized on the number of scans and mass. All aliquots of a given sample were measured within a short time interval (<1 h). This procedure was repeated twice over two successive days without removing the enamel from the ESR tubes between measurements in order to evaluate measurement precision and thus D_E reproducibility.

Fitting procedures were carried out with the Microcal OriginPro 9.1. software using a Levenberg-Marquardt algorithm by chi-square minimisation. Data were weighted by the inverse of the squared ESR intensity ($1/I^2$) [6]. D_E values were obtained by fitting a single saturating exponential function through the pooled mean ESR intensities obtained from the two repeated measurements. To ensure reliable results, the maximum irradiation dose (D_{max}) was adjusted to meet the criteria defined by [7] : for D_E values of > 1000 Gy the D_{max}/D_E ratio has to be kept between 1 and 2. Consequently, final D_E values were calculated by using D_{max}= 1805 Gy.

For the dose rate calculations the following parameters were used: an alpha efficiency of 0.13±0.02 [8]. Monte-Carlo beta attenuation factors from [9] dose-rate conversion factors from [10] and estimated water content of 5±3 wt.% in sediment. The gamma dose rate was measured in situ with a portable gamma spectrometer. Representative sediment samples were analysed for U, Th and K by ICP-MS. ESR age calculations were carried out using the DATA program [11]. The results are shown in Table C. All errors are 1-σ.

References

1. Eggins, S., Grün, R., Pike, A., Shelley, A., Taylor, L., 2003. ^{238}U, ^{232}Th profiling and U-series isotope analysis of fossil teeth by laser ablation ICPMS. Quaternary Science Reviews 22, 1373-1382.

10. Guérin, G., Mercier, N., Adamiec, G., 2011. Dose rate conversion factors: update. Ancient TL 29, 5-8.

11. Grün, R., 2009. The DATA program for the calculation of ESR age estimates on tooth enamel. Quaternary Geochronology 4: 231-232.

2. Eggins, S.M., Grün, R., McCulloch, M.T., Pike, A.W.G., Chappell, J., Kinsley, L., Shelley, M., Murray-Wallace, C.V., Spötl, C., Taylor, L. 2005. In situ U-series dating by laser ablation multi-collector ICPMS: new prospects for Quaternary geochronology. Quaternary Science Reviews 24, 2523-2538.

3. Grün, R., Eggins, S., Kinsley, L., Mosely, H., Sambridge, M., 2014. Laser ablation U-series analysis of fossil bones and teeth. Palaeogeography, Palaeoclimatology, Palaeoecology 416, 150-167.

4. Duval, M., Aubert, M., Hellstrom, J., Grün, R., 2011. High resolution, LA-ICP-MS mapping of U and Th isotopes in an Early Pleistocene equid tooth from Fuente Nueva-3 (Orce, Andalusia, Spain). Quaternary Geochronology 6, 458-467.
5. Grün, R., 2000a. Methods of dose determination using ESR spectra of tooth enamel. Radiation Measurements 32: 767-772.
6. Grün, R., Brumby, S., 1994. The assessment of errors in the past radiation doses extrapolated from ESR/TL dose response data. Radiation Measurements 23: 307-315.
7. Duval M., Grün, R., 2016. Are published ESR dose assessments on fossil tooth enamel reliable? Quaternary Geochronology 31, 19-27.
8. Grün, R., Katzenberger-Apel, O., 1994. An alpha irradiator for ESR dating. Ancient TL 12: 35-38.
9. Marsh, R.E., 1999. Beta-gradient Isochrons Using Electron Paramagnetic Resonance: Towards a New Dating Method in Archaeology. MSc thesis, McMaster University.

Table A: Samples for ESR and U-series analysis

	**Sample**	**Layer**	**Origin**
3448	*Bos* lower molar	d	Pigorini Museum
3449	*Equus* lower molar	m	Pigorini Museum
3450	Bos lower jaw fragment with M/1-3	m	IsIPU Anagni
3451	*Bos* tooth fragment	d	IsIPU Anagni
3452	*Bos* upper molar	d	IsIPU Anagni
3453	*Bos* lower molar	d	IsIPU Anagni
3454	*Bos* upper molar	d	IsIPU Anagni
3455	*Elephas* tooth fragment (no enamel)	m	Villa-Soriano excavation

Table B: U-series results on bones and teeth from Torre in Pietra. No age calculations were carried out for U concentrations of ≤ 0.5 ppm or U/Th ≤ 300, but not negative (indicated in red). Negative U/Th are due to the background being higher than the measurement. All errors are 2-σ. Individual results do not contain errors of standard measurement (correlated errors), mean values incorporate errors of standard. CS calculations are based on closed system assumptions while diffusion (Diff) ages are based on the continuous diffusion assumption of Sambridge et al. (2012). Bold green age estimates are in agreement with Ar/Ar results and other independent lines of chronological evidence (see text).

**3448 Torre in Pietra**	U (ppm)	Th (ppb)	U/Th	^{230}Th/^{238}U	^{230}Th/^{238}U error	^{234}U/^{238}U	^{234}U/^{238}U error	CS Age (ka)	CS Age error (ka)	Diff Age (ka)	Diff Age error (ka)
1	5.21	133	**39**	0.9011	0.0485	1.0663	0.0234	n/a			
2	1.22	0	-6422	0.8132	0.0859	1.1125	0.0613	138.3	33.3	143.4	31.3
3	0.72	1	1005	0.8983	0.1182	1.0877	0.0882	182.2	74.2	190.8	68.1
4	**0.07**	-1	-131	1.0283	1.2802	1.1800	1.6305	n/a			
5	1.49	0	-3523	0.6324	0.0689	1.0556	0.0534	98.7	19.5	99.8	17.8
6	3.45	1	3676	0.9023	0.0533	1.1198	0.0352	169.5	27.2	178.9	26.8
7	2.61	0	-64213	0.8330	0.0513	1.1052	0.0375	147.4	22.1	153.0	20.6
8	9.71	2	5630	1.0775	0.0417	1.2503	0.0232	189.9	20.8	216.1	26.4
9	119	1	109532	1.1784	0.0128	1.1931	0.0065	306.0	18.6	480.0	94.6
10	225	5	45271	1.2095	0.0158	1.1797	0.0034	389.4	41.2	leaching	
11	233	3	88392	1.2028	0.0173	1.1742	0.0042	390.9	46.6	leaching	
12	236	3	74001	1.2062	0.0130	1.1764	0.0049	392.1	37.4	leaching	
13	250	7	34963	1.1570	0.0177	1.1764	0.0042	305.1	23.3	448.2	99.7
14	252	1	333868	1.1730	0.0147	1.1696	0.0038	341.1	26.6	leaching	
15	234	1	229212	1.1825	0.0152	1.1682	0.0047	362.4	33.7	leaching	
16	237	1	217435	1.1985	0.0117	1.1669	0.0036	405.5	37.0	leaching	
17	231	0	494698	1.1940	0.0160	1.1633	0.0039	406.8	50.1	leaching	
18	210	3	74942	1.2296	0.0147	1.1521	0.0036	leaching			
19	225	1	297304	1.1991	0.0203	1.1627	0.0044	425.7	74.0	leaching	
20	122	1	114455	1.1880	0.0161	1.1824	0.0055	338.9	28.9	leaching	
21	1.33	3	423	0.7401	0.0910	1.1276	0.0659	113.3	27.1	116.6	25.6
22	4.28	4	1049	0.9566	0.0481	1.2119	0.0327	157.4	19.6	170.1	20.6
23	4.38	9	485	0.4652	0.0397	1.0273	0.0303	65.6	8.2	65.9	7.7
24	33.7	1864	**18**	1.8729	0.0856	1.0804	0.0120	n/a			
25	97.6	3146	**31**	1.8124	0.0460	1.0808	0.0075	n/a			
26	231	1291	**179**	0.7793	0.0114	1.0827	0.0049	n/a			
27	239	158	1516	0.7974	0.0097	1.0795	0.0043	142.6	3.8	146.6	3.8
28	139	660	**210**	1.6861	0.0603	1.1102	0.0067	n/a			
28	9.83	9728	**1**	3.4565	0.2670	1.0610	0.0249	n/a			
30	29.3	8351	**4**	3.6612	0.1596	1.0529	0.0169	n/a			
**Mean Values**											
2-7 Enamel	1.59±1.01	1.01		0.8305	0.0334	1.1029	0.0242	147.2	14.4	152.7	15.2
9-19 Dentine	223±22	21.89		1.1936	0.0189	1.1703	0.0056	380.7	48.3	leaching	
21-23 Enamel	3.33±2.00	2.00		0.7125	0.0346	1.1198	0.0219	107.6	9.6	110.4	10.0
26-28 Cement	239			0.7974	0.0097	1.0795	0.0043	142.6	3.8	146.6	3.8

**3449 Torre in Pietra**	U (ppm)	Th (ppb)	U/Th	$^{230}Th/^{238}U$	$^{230}Th/^{238}U$ error	$^{234}U/^{238}U$	$^{234}U/^{238}U$ error	CS Age (ka)	CS Age error (ka)	Diff Age (ka)	Diff Age error (ka)
1	373	17500	**21**	1.6857	0.0071	1.2540	0.0035	n/a			
2	357	35	10117	1.3159	0.0071	1.2150	0.0032	leaching			
3	254	72	3537	1.5351	0.0092	1.2964	0.0047	leaching			
4	256	39	6520	1.3563	0.0096	1.3264	0.0052	319.7	13.1	leaching	
5	255	82	3099	1.3717	0.0077	1.3516	0.0037	303.1	8.6	leaching	
6	261	51	5133	1.3670	0.0068	1.3259	0.0039	333.4	10.6	leaching	
7	285	74	3832	1.4401	0.0100	1.3476	0.0045	406.3	25.1	leaching	
8	366	165	2216	1.4394	0.0077	1.3123	0.0048	620.1	117.6	leaching	
9	443	10	44427	1.2176	0.0093	1.1694	0.0058	463.3	57.0	leaching	
10	1.78	2	1024	0.8738	0.0845	1.1185	0.0445	158.3	36.8	166.0	37.7
11	**0.02**	1	**17**	1.1457	17.6387	2.1355	27.0488	n/a			
12	**0.14**	2	**71**	1.0257	0.3532	1.4381	0.3475	n/a			
13	**0.18**	1	**148**	0.9319	0.5044	1.0162	0.3867	n/a			
14	1.24	0	4056	1.0692	0.2140	1.1597	0.1908	239.8	226.8	278.4	258.1
15	253	1	290586	1.2296	0.0126	1.1904	0.0084	404.8	47.1	leaching	
16	192	3	71731	1.2446	0.0183	1.2507	0.0108	300.4	24.3	581.2	326.8
17	174	1	141489	1.2003	0.0190	1.2800	0.0071	236.8	12.7	303.2	26.0
18	143	4	31920	1.0721	0.0157	1.2527	0.0084	186.5	7.5	211.5	9.5
19	114	3	39700	1.0433	0.0205	1.2588	0.0095	172.7	8.4	192.7	10.4
20	93.4	2	40647	1.0155	0.0195	1.2555	0.0097	163.8	7.5	180.7	8.9
**Mean Values**											
2-9 Cement	309±50			1.3686	0.0212	1.2820	0.0062	451.8	77.7	leaching	
10-14 Enamel	0.67±0.71			0.9572	0.1421	1.1487	0.1322	181.5	89.0	195.6	102.5
15-20 Dentine	162±47			1.1616	0.0192	1.2419	0.0069	239.5	13.7	299.1	27.0

**3450 Torre in Pietra**	U (ppm)	Th (ppb)	U/Th	$^{230}Th/^{238}U$	$^{230}Th/^{238}U$ error	$^{234}U/^{238}U$	$^{234}U/^{238}U$ error	CS Age (ka)	CS Age error (ka)	Diff Age (ka)	Diff Age error (ka)
1	**0.02**	1	10	2.2804	78.3054	-0.7909	7.5610	n/a			
2	**0.07**	1	74	1.2849	0.9154	0.4557	0.3097	n/a			
3	**0.53**	0	1922	0.9442	0.1868	1.1105	0.0869	n/a			
4	2.35	1	1260	1.1597	0.0743	1.3011	0.0373	203.2	38.4	242.3	57.4
5	**0.39**	1	**264**	0.7142	0.1564	0.9795	0.1024	n/a			
6	97.0	1	67261	1.3861	0.0220	1.5562	0.0087	189.6	7.5	241.7	14.1
7	127	0	276188	1.3778	0.0237	1.5795	0.0072	180.0	7.2	224.7	12.9
8	128	1	131183	1.3768	0.0227	1.5780	0.0065	180.2	6.8	224.9	12.3
9	133	2	65134	1.3713	0.0231	1.5869	0.0073	176.1	6.7	217.9	11.7
10	136	2	54958	1.3676	0.0266	1.5898	0.0045	174.4	7.4	214.9	13.1
11	142	3	43819	1.3646	0.0197	1.5895	0.0073	173.6	5.7	213.5	9.6
12	146	1	141535	1.3692	0.0224	1.5861	0.0056	175.8	6.4	217.3	11.3
13	152	3	40755	1.3523	0.0219	1.5813	0.0064	172.4	6.2	211.0	10.4
14	158	2	57128	1.3586	0.0194	1.5709	0.0054	176.9	5.7	218.5	10.0
15	163	3	40866	1.3599	0.0170	1.5482	0.0070	183.9	5.6	230.1	9.9
16	174	2	54442	1.3455	0.0196	1.5067	0.0063	192.8	7.0	244.0	13.3
17	165	4	29970	1.3191	0.0197	1.4754	0.0051	194.7	7.3	245.2	13.8
**Mean Values**											
1-5 Enamel	0.67±0.86			1.0823	0.2075	1.2041	0.0710	216.4	127.3	250.2	194.6
8-17 Dentine	148±10			1.3589	0.0218	1.5601	0.0075	180.1	6.8	223.7	12.1

**3451 Torre in Pietra**	U (ppm)	Th (ppb)	U/Th	$^{230}Th/^{238}U$	$^{230}Th/^{238}U$ error	$^{234}U/^{238}U$	$^{234}U/^{238}U$ error	CS Age (ka)	CS Age error (ka)	Diff Age (ka)	Diff Age error (ka)
1	2.99	6	496	0.6023	0.0404	1.0540	0.0353	91.6	10.9	92.5	9.8
2	8.36	-1	-8077	0.5941	0.0236	1.0133	0.0154	96.1	6.6	96.4	6.1
3	3.63	1	3321	0.7948	0.0501	1.0158	0.0365	165.3	29.4	166.6	24.7
4	373	2	175942	1.2051	0.0116	1.1149	0.0070	leaching			
5	296	1	380414	1.1937	0.0082	1.2171	0.0065	289.9	11.9	431.3	38.4
6	260	2	154585	1.1659	0.0121	1.2322	0.0090	250.3	11.7	319.2	20.0
7	236	2	147060	1.1447	0.0126	1.2348	0.0082	233.7	9.9	285.8	15.3
8	275	1	386468	1.1478	0.0086	1.2293	0.0075	239.9	8.1	296.4	11.5
9	284	10	29205	1.2149	0.0114	1.2137	0.0081	319.9	20.4	leaching	
10	286	2	125312	1.1402	0.0100	1.2086	0.0070	251.4	9.9	313.7	16.0
11	299	1	573970	1.0634	0.0124	1.2195	0.0059	197.9	6.7	224.5	8.7
12	300	1	242899	1.0188	0.0108	1.2178	0.0086	178.7	5.6	197.8	5.9
13	268	0	699345	1.0630	0.0176	1.2109	0.0084	202.1	9.9	229.6	13.0
14	1.77	0	-13132	0.7472	0.0761	1.1279	0.0451	115.1	22.2	118.6	21.8
15	5.96	-1	-4089	0.9286	0.0619	1.2703	0.0227	133.5	17.2	143.4	19.8
16	5.92	0	-26692	0.8860	0.0508	1.2087	0.0288	135.9	16.3	144.2	17.2
17	8.72	29	306	0.9684	0.0320	1.2673	0.0252	145.3	11.4	157.7	11.7
18	164	578	284	1.1848	0.0281	1.1827	0.0098	333.0	48.3	leaching	0.0
19	266	93	2875	1.0980	0.0184	1.1703	0.0062	252.9	16.2	304.0	27.7
20	311	54	5805	1.0980	0.0161	1.1881	0.0140	237.5	15.8	281.5	19.5
21	306	66	4631	1.1493	0.0183	1.1966	0.0081	270.6	18.9	352.6	59.5
**Mean Values**											
1-3 Enamel	4.99±3.39			0.6444	0.0217	1.0220	0.0149	108.2	6.9	108.7	6.8
4-13 Dentine	288±23			1.1371	0.0178	1.2064	0.0061	**250.9**	**14.7**	312.0	28.9
14-17 Enamel	5.59±2.87			0.9185	0.0292	1.2416	0.0154	137.3	9.0	147.0	10.6
18-21 Cement	262±68			1.1266	0.0198	1.1852	0.0076	**262.4**	**18.8**	328.3	38.6

**3452 Torre in Pietra**	U (ppm)	Th (ppb)	U/Th	$^{230}Th/^{238}U$	$^{230}Th/^{238}U$ error	$^{234}U/^{238}U$	$^{234}U/^{238}U$ error	CS Age (ka)	CS Age error (ka)	Diff Age (ka)	Diff Age error (ka)
1	437.75	9042	48	1.0508	0.0102	1.1077	0.0126	278.3	20.5	324.2	19.6
2	589.77	86	6861	0.9785	0.0212	1.0741	0.0147	245.2	24.3	264.3	24.3
3	9.36	1	15077	0.7702	0.0326	1.1274	0.0275	121.5	11.0	125.5	10.0
4	3.18	-1	-3023	1.0859	0.0675	1.4434	0.0531	136.8	18.7	152.4	20.6
5	4.02	-2	-2427	1.0714	0.0647	1.3651	0.0347	150.2	19.9	167.8	24.1
6	6.96	0	162554	1.0086	0.0345	1.3130	0.0237	145.5	11.3	159.6	12.4
7	23.5	-1	-18817	0.9978	0.0277	1.3191	0.0167	141.1	8.4	154.2	9.4
8	88.9	2	38355	1.2450	0.0138	1.3834	0.0145	202.9	8.5	251.4	10.9
9	194	1	374175	1.4439	0.0194	1.4464	0.0175	269.6	18.7	696.0	857.9
10	195	0	-5479689	1.5023	0.0166	1.4527	0.0127	308.2	20.2	leaching	
11	189	1	154428	1.4744	0.0209	1.4496	0.0163	288.5	21.9	leaching	
12	185	2	93180	1.4832	0.0246	1.4538	0.0104	291.3	21.4	leaching	
13	191	2	77740	1.4924	0.0230	1.4544	0.0125	298.0	22.5	leaching	
14	167	4	43130	1.4992	0.0161	1.4485	0.0131	310.1	20.6	leaching	
15	175	9	20000	1.4289	0.0316	1.4474	0.0088	259.4	20.2	475.9	186.0
16	173	705	**246**	1.4235	0.0215	1.4324	0.0112	n/a			
17	5.26	7323	**1**	0.4816	0.0348	0.9750	0.0241	n/a			
18	102	31905	**3**	1.5288	0.0173	1.4014	0.0160	n/a			
19	160	28	5611	1.5204	0.0228	1.4352	0.0148	350.2	36.8	leaching	
20	177	6	31986	1.5175	0.0186	1.4391	0.0087	340.7	24.9	leaching	
21	181	3	57010	1.5409	0.0161	1.4444	0.0105	361.2	28.2	leaching	
22	184	0	-3252196	1.5509	0.0150	1.4529	0.0082	359.8	24.1	leaching	
23	180	0	490297	1.5886	0.0101	1.4481	0.0083	435.7	35.2	leaching	
24	184	1	197959	1.6096	0.0172	1.4461	0.0116	506.8	93.1	leaching	
25	115	1	97096	1.4130	0.0198	1.4077	0.0107	280.3	17.6	leaching	
26	7.99	-1	-12102	1.0404	0.0376	1.2985	0.0217	158.9	13.6	176.4	15.9
27	3.47	-1	-3428	1.1186	0.0670	1.3177	0.0445	179.4	29.6	206.4	36.7
28	7.08	0	-19379	1.0649	0.0538	1.3132	0.0239	162.4	18.8	181.9	23.7
28	12.5	0	32959	0.9838	0.0329	1.1910	0.0234	175.0	16.5	191.0	17.3
30	362	2	154290	1.1422	0.0155	1.1788	0.0086	284.6	19.7	379.0	58.0
**Mean Values**											
1-2 Cement	514±152			1.0093	0.0204	1.0884	0.0112	**258.6**	**23.9**	**286.4**	**31.3**
3-6 Enamel	5.88±2.83			0.9349	0.0264	1.2657	0.0174	136.2	8.2	146.5	9.6
9-15 Dentine	185±8			1.4750	0.0241	1.4505	0.0084	**288.2**	**19.9**	leaching	
19-25 Dentine	169±19			1.5417	0.0245	1.4410	0.0078	368.5	37.7	leaching	
26-28 Enamel	6.18±2.76			1.0644	0.0333	1.3077	0.0166	163.9	12.1	183.7	16.0
29-30 Cement	187±350			1.1369	0.0230	1.1792	0.0100	**278.7**	**26.1**	363.4	63.6

**3453 Torre in Pietra**	U (ppm)	Th (ppb)	U/Th	$^{230}Th/^{238}U$	$^{230}Th/^{238}U$ error	$^{234}U/^{238}U$	$^{234}U/^{238}U$ error	CS Age (ka)	CS Age error (ka)	Diff Age (ka)	Diff Age error (ka)
1	1.58	81	**19**	1.0422	0.1693	1.1301	0.0651	n/a			
2	**0.01**	0	**43**	14.9789	96.7970	-3.9867	60.2203	n/a			
3	**0.15**	0	2188	0.7838	0.3937	0.7422	0.3349	n/a			
4	1.14	1	1440	0.8035	0.1142	1.0524	0.0712	154.1	54.3	157.4	50.1
5	3.20	0	-7756	0.6740	0.0656	1.0354	0.0255	113.9	20.2	114.9	19.8
6	1.26	0	13821	0.6106	0.0962	0.9094	0.0765	125.3	44.9	121.6	35.2
7	6.48	0	-147969	1.0559	0.0581	1.2583	0.0275	177.7	25.0	199.5	31.4
8	160	2	77124	1.2073	0.0142	1.2074	0.0056	320.3	21.5	1019.8	14654.5
9	159	3	54098	1.2180	0.0128	1.2446	0.0051	281.2	13.2	419.9	52.4
10	160	4	42839	1.2499	0.0085	1.2630	0.0066	290.4	11.4	499.0	70.9
11	155	4	37638	1.2436	0.0136	1.2712	0.0060	275.7	13.2	418.5	54.2
12	155	2	64285	1.2199	0.0141	1.2744	0.0070	254.1	11.7	344.2	28.3
13	151	3	45909	1.1940	0.0157	1.2978	0.0052	221.9	8.9	275.8	16.5
14	162	2	77740	1.2158	0.0144	1.2915	0.0050	238.4	9.5	309.8	20.8
15	160	3	59226	1.1752	0.0157	1.2922	0.0069	215.3	8.9	262.3	14.7
16	171	3	50547	1.2157	0.0164	1.2930	0.0054	237.3	10.7	307.6	23.2
17	173	2	70213	1.2078	0.0211	1.2878	0.0045	236.1	13.2	303.4	28.7
18	175	2	76234	1.2014	0.0126	1.2826	0.0056	235.7	8.6	301.3	17.0
19	186	7	28586	1.1983	0.0128	1.2521	0.0063	257.2	11.2	343.6	25.9
20	196	11	18565	1.2199	0.0179	1.2352	0.0045	294.3	19.7	479.5	128.1
**Mean Values**											
1-6 Enamel	1.15±1.14			0.7069	0.0518	0.9970	0.0351	134.9	22.4	134.8	21.6
8-20 Dentine	166±7			1.2127	0.0191	1.2681	0.0061	**254.1**	**14.7**	341.8	38.5

**3454**<br>**Torre in Pietra**	U (ppm)	Th (ppb)	U/Th	$^{230}Th/^{238}U$	$^{230}Th/^{238}U$ error	$^{234}U/^{238}U$	$^{234}U/^{238}U$ error	CS Age (ka)	CS Age error (ka)	Diff Age (ka)	Diff Age error (ka)
1	19.60	17400	**1**	1.2272	0.0356	1.0956	0.0144	n/a			
2	4.01	2	2384	0.9975	0.0562	1.0916	0.0285	244.3	56.2	267.7	65.3
3	9.40	2	4094	0.9431	0.0400	1.0740	0.0260	217.1	34.3	229.8	33.4
4	7.95	-1	-9044	0.9794	0.0473	1.0778	0.0228	242.1	46.8	261.3	52.4
5	8.24	0	70275	0.9804	0.0395	1.0618	0.0225	260.7	49.9	280.5	53.0
6	7.83	1	14777	0.9876	0.0430	1.1069	0.0190	223.3	33.6	243.3	39.4
7	19.8	1	15483	1.1881	0.0422	1.1399	0.0165	534.8	430.4	leaching	
8	97.8	9	11243	1.3557	0.0202	1.2084	0.0068	leaching			
9	113	5	20526	1.2894	0.0227	1.1568	0.0070	leaching			
10	97.0	6	16436	1.2914	0.0213	1.1863	0.0081	leaching			
11	75.9	6	11979	1.3371	0.0271	1.2320	0.0068	leaching			
12	74.5	8	9072	1.3411	0.0275	1.2259	0.0083	leaching			
13	75.3	8	9745	1.3632	0.0274	1.2493	0.0081	leaching			
14	69.7	8	8794	1.3431	0.0210	1.2629	0.0095	454.0	88.3	leaching	
15	64.9	8	8431	1.3943	0.0284	1.2531	0.0096	leaching			
16	63.1	9	7238	1.2181	0.0234	1.2433	0.0079	282.7	23.9	425.6	101.5
17	74.1	14	5116	1.2738	0.0241	1.2425	0.0085	353.1	44.2	leaching	
18	78.3	17	4532	1.4053	0.0262	1.2579	0.0072	leaching			
19	80.2	18	4413	1.4166	0.0295	1.2619	0.0071	leaching			
20	79.7	19	4216	1.4025	0.0232	1.2536	0.0081	leaching			
21	85.0	29	2958	1.4394	0.0234	1.2305	0.0076	leaching			
22	88.7	52	1691	1.4799	0.0203	1.2442	0.0066	leaching			
23	59.4	1455	**41**	1.7624	0.0256	1.2332	0.0111	leaching			
24	90.4	82	1107	1.4878	0.0238	1.2777	0.0088	leaching			
25	76.7	16	4850	1.4276	0.0180	1.2621	0.0078	leaching			
26	70.0	12	5721	1.4172	0.0194	1.2633	0.0076	leaching			
**Mean Values**											
2-6<br>Enamel	7.49±1.82			0.9741	0.0249	1.0809	0.0119	**234.8**	**22.9**	**252.8**	**27.5**
8-22<br>Dentine	81.1±6.9			1.3572	0.0218	1.2301	0.0060	leaching			

**3455 Torre in Pietra**	U (ppm)	Th (ppb)	U/Th	$^{230}Th/^{238}U$	$^{230}Th/^{238}U$ error	$^{234}U/^{238}U$	$^{234}U/^{238}U$ error	CS Age (ka)	CS Age error (ka)	Diff Age (ka)	Diff Age error (ka)
1	203	33	6152	1.3781	0.0100	1.3597	0.0065	299.8	11.8	leaching	
2	223	12	18244	1.2943	0.0205	1.3121	0.0048	277.1	17.5	469.3	125.4
3	280	16	18001	1.2925	0.0153	1.2577	0.0039	351.6	25.9	leaching	
4	352	14	24916	1.1760	0.0173	1.1813	0.0039	322.4	26.0	589.3	353.7
5	347	9	37419	1.1923	0.0145	1.1821	0.0037	346.8	27.0	leaching	
6	314	11	28925	1.2145	0.0143	1.2056	0.0041	334.1	23.2	leaching	
7	285	11	25360	1.2307	0.0192	1.2084	0.0038	354.9	35.6	leaching	
8	257	12	207 86	1.2636	0.0171	1.2093	0.0052	430.7	61.1	leaching	
9	270	10	26379	1.2251	0.0123	1.1995	0.0052	366.0	27.9	leaching	
10	272	11	24696	1.2345	0.0148	1.2171	0.0046	342.3	25.3	leaching	
11	275	10	28784	1.2300	0.0102	1.2032	0.0043	366.1	23.1	leaching	
12	273	8	35827	1.2470	0.0130	1.2134	0.0071	373.7	33.2	leaching	
13	276	12	22741	1.2652	0.0100	1.2189	0.0072	398.2	34.6	leaching	
14	267	15	17832	1.2773	0.0128	1.2267	0.0064	402.9	39.2	leaching	
15	226	43	5197	1.3140	0.0148	1.2516	0.0045	411.5	41.9	leaching	
16	257	58	4462	1.3141	0.0129	1.2501	0.0055	417.3	40.9	leaching	
17	275	26	10496	1.2970	0.0134	1.2264	0.0087	468.9	77.6	leaching	
18	279	28	10110	1.2877	0.0129	1.2342	0.0089	403.8	44.4	leaching	
19	296	14	20830	1.2355	0.0146	1.2039	0.0068	375.7	36.8	leaching	
20	302	7	40845	1.2640	0.0136	1.2093	0.0061	432.4	53.2	leaching	
21	311	11	27427	1.2224	0.0173	1.1819	0.0093	417.1	66.6	leaching	
22	314	8	40240	1.2393	0.0131	1.1966	0.0084	409.0	49.2	leaching	
23	334	24	13949	1.2396	0.0117	1.1832	0.0079	478.1	81.3	leaching	
**Mean Values**											
1-3 Outer Bone	235±46			1.3177	0.0223	1.3042	0.0067	307.8	25.2	leaching	
4-23 Inner Bone	289±14			1.2455	0.0194	1.2083	0.0058	**384.8**	**47.7**	leaching	
1-23 Whole Bone	282±15			1.2533	0.0195	1.2188	0.0058	**372.2**	**42.4**	leaching	

Table C: ESR Results. All errors are 1-σ. Bold green age estimates are in agreement with Ar/Ar results and other independent lines of chronological evidence (see text).

		ENAMEL					DENTINE			SEDIMENT		
Sample	De (Gy)	U(ppm)	$^{234}U/^{238}U$	$^{230}Th/^{234}U$	Thickness (µm)	Layer Removed (µm)	U (ppm)	$^{234}U/^{238}U$	$^{230}Th/^{234}U$	U(ppm)	Th(ppm)	K(%)
3448	918±16	1.59±0.05	1.1029±0.0121	0.7530±0.0151	1240±100	85±25	223±11	1.1703±0.0028	1.0199±0.0081	8.13±1.62	23.1±4.2	0.93±0.14
3449	1711±81	0.76±0.36	1.2419±0.0035*	0.9353±0.0077*	1150±100	75±50	162±24	1.2419±0.0035	0.9353±0.0077	7.8±0.4	21.2±2.6	0.3±0.1
3450	1653±35	0.67±0.43	1.5601±0.0038*	0.8710±0.0070*	1230±50	50±20	148±5	1.5601±0.0038	0.8710±0.0070	7.8±0.4	21.2±2.6	0.3±0.1
3451	1858±48	5.30±1.50	1.2416±0.0077	0.7398±0.0118	1145±100	140±25	288±12	1.2064±0.0031	0.9426±0.0074	8.13±1.62	23.1±4.2	0.93±0.14
3452	1089±47	6.08±1.45	1.2657±0.0087	0.7386±0.0104	1235±100	160±50	185±4	1.4505±0.0041	1.0169±0.0083	8.13±1.62	23.1±4.2	0.93±0.14
3453	737±19	1.14±0.57	0.9970±0.01760	0.7090±0.0260	1120±100	65±25	166±4	1.2681±0.0031	0.9563±0.0075	8.13±1.62	23.1±4.2	0.93±0.14
3454	938±20	7.48±0.91	1.0809±0.0060	0.9012±0.0115	1150±100	160±25	81.0±3.5	1.2301±0.0030	1.1033±0.0089	8.13±1.62	23.1±4.2	0.93±0.14

*Because of low U-concentrations in enamel, dentine U-series values were applied

Sample	Sediment γ-dose rate (µGy/a)	Sediment β-dose rate (µGy/a)	internal dose rate (µGy/a)	Dentine β-dose rate (µGy/a)	Total dose rate (µGy/a)	Age (ka)	internal dose rate (µGy/a)	Dentine β-dose rate (µGy/a)	Total dose rate (µGy/a)	p-value	Age (ka)	Age (ka)
			CLOSED SYSTEM (EU) CALCULATIONS				US-ESR CALCULATIONS					CSUS-ESR
3448	2163±180	237±40	519±59	2625±206	5544±283	165±8			no solution			-203±52
3449	1920±130	203±28	351±168	2614±453	5088±501	**336±36**	294±160	2155±549	4572±586	-0.86±0.07	**374+46/-36**	**424±48**
3450	1920±130	198±16	367±235	2638±158	5123±312	**322±20**	248±171	1744±186	4110±284	-0.47±0.05	**402+30/-22**	559±38
3451	2163±180	239±41	2115±597	3719±872	8236±1073	**225±29**			no solution			**271±32**
3452	2163±180	216±41	2226±525	2126±233	6731±603	161±16			no solution			-16±27
3453	2163±180	269±46	2408±349	1137±135	5941±417	157±11			no solution			18±12
3454	2163±180	233±41	2437±351	911±87	5744±406	163±12			no solution			44±87
