# Supplementary material for: The Acheulian and Early Middle Paleolithic in Latium (Italy): Stability and Innovation
Source: PLoS One. 2016 Aug 15;11(8):e0160516. doi: 10.1371/journal.pone.0160516 (PMC4985512; doi:10.1371/journal.pone.0160516)
Supplement: S4 File — (PDF) [file pone.0160516.s004.pdf]

Supporting Information

The Acheulian and early Middle Paleolithic in Latium (Italy): Stability and Innovation

Paola Villa*, Sylvain Soriano, Rainer Grün, Fabrizio Marra, Sebastien Nomade, Alison Pereira, Giovanni Boschian, Luca Pollarolo, Fang Fang, Jean-Jacques Bahain

*To whom correspondence should be addressed. E-mail: villap@colorado

S4 File. Permissions from copyright holders

This PDF file includes: Permission from TINITALY/01 DEM and from INGV for figure 1

Permission to use and modify insets in figures 5, 6, 9 and S1 File fig. B

TINITALY DEM

Simone Tarquini <simone.tarquini@ingv.it>

Wed 6/8/2016 7:05 AM

Inbox

To:Paola Villa <paola.villa@colorado.edu>;

Dear user,

Thank you for your request. To download data, you have to log in at the directory:

http://tinitaly.pi.ingv.it/paola.villa/

Your system should require user and pass, that is for you

user: paola.villa
pass: pv6y4s0

then you can copy all the zipped files there. File format is "ESRI ASCII Raster". Data is in Universal Transverse Mercator coordinate system (UTM), World Geodetic System WGS 84. Notice that all the DEM is projected in zone 32N, even if eastern italian regions are in zone 33N (in case, you need to re-project portions of the DEM).

The content includes four papers describing to some extent the DEM database (including input data and interpolation algorithms). Do not hesistate to contact me for any problem in downloading/using data or if you need a broader area.

I recall below terms and conditions of use:

(i) data is provided for research purposes only;

(ii) data is provided solely to the person named on this application form and should not be given to third parties. Third parties who might need access to the same dataset are required to fill their own application forms.

(iii) data must be referenced with the following citations:

1. Tarquini S., Isola I., Favalli M., Mazzarini F., Bisson M., Pareschi M. T., Boschi E. (2007). TINITALY/01: a new Triangular Irregular Network of Italy, Annals of Geophysics 50, 407 - 425.

2. Tarquini S., Vinci S., Favalli M., Doumaz F., Fornaciai A., Nannipieri L., (2012). Release of a 10-m-resolution DEM for the Italian territory: Comparison with global-coverage DEMs and anaglyph-mode exploration via the web, Computers & Geosciences 38, 168-170. doi: doi:10.1016/j.cageo.2011.04.018

(iv) Our aim is to provide scientific information to members of national and international scientific communities. The Istituto Nazionale di Geofisica e Vulcanologia assumes no responsibility for the downloaded data, which is not necessarily updated. The global accuracy of the digital elevation model id described in the above reference #1. Nevertheless, we cannot exclude the presence of local higher errors.

Thank you for your interest in our work.

Kind regards

Simone Tarquini & colleagues at INGV Pisa

Simone Tarquini
Istituto Nazionale di Geofisica e Vulcanologia
Sezione di Pisa
Via della Faggiola, 32
56126 PISA, ITALY
tel. +39 050-8311932
fax. +39 050-8311942
simone.tarquini@ingv.it
http://ingv.academia.edu/SimoneTarquini

Permission

presidenza IsIPU <presidente@isipu.org>

Wed 3/30/2016 2:39 PM

To:Paola Villa <paola.villa@colorado.edu>;

Importance: High

Dear Paola,

you have my permission to use two figures published by A. Malatesta in Quaternaria 1978 with the stratigraphy of Torre in Pietra (figs 2 and 4). You also have my permission to use fig. 4 of Piperno and Biddittu's paper published in the same Quaternaria 1978 issue and to use in modified form various figures of artifacts of their papers. The source must be cited.

Fabio Parenti, President of the Italian Institute of Human Paleontology

INGV
terremoti
vulcani
ambiente

ISTITUTO NAZIONALE
DI GEOFISICA E VULCANOLOGIA

Rome, June 9, 2016

to: Fabrizio Marra

Primo Ricercatore
Istituto Nazionale di Geofisica e Vulcanologia
Via di Vigna Murata 605, 00143, Roma
tel. 06 51860420
fax 06 51860507
fabrizio.marra@ingv.it

Dear Dr. Marra

you have my permission to use and modify if necessary the Digital Elevation Map (DEM) for the region of Rome (WA 6570) for your research.

Dr. Fabio Florindo

Director, Environment Department
Istituto Nazionale di Geofisica e Vulcanologia
Rome - Italy
fabio.florindo@ingv.it

Fabio Florindo
