# Supplementary material for: The Acheulian and Early Middle Paleolithic in Latium (Italy): Stability and Innovation
Source: PLoS One. 2016 Aug 15;11(8):e0160516. doi: 10.1371/journal.pone.0160516 (PMC4985512; doi:10.1371/journal.pone.0160516)
Supplement: S5 File — (PDF) [file pone.0160516.s005.pdf]

Supporting Information

The Acheulian and early Middle Paleolithic in Latium (Italy): Stability and Innovation

Paola Villa*, Sylvain Soriano, Rainer Grün, Fabrizio Marra, Sebastien Nomade, Alison Pereira, Giovanni Boschian, Luca Pollarolo, Fang Fang, Jean-Jacques Bahain

*To whom correspondence should be addressed. E-mail: villap@colorado

S5 File. Lithic analysis

This PDF file includes:

Lithic analysis procedures

References

Our analysis of lithic assemblages is directed to the identification and description of similarities and differences in reduction sequences, in the objectives of production and in the selection of blanks for tool manufacture. Since interassemblage comparison is the fundamental tool of our research we use a standard nomenclature and coding forms.

Once sorted with procedures described in the text "Sorting and Sampling", each major class of artifacts (bifaces, small formal tools, cores, unretouched flakes) is recorded by catalog number, provenience and raw material in separate Excel files. Length, breadth and thickness (in mm) were measured by digital caliper, according to the morphological axis (if a tool) or the debitage axis (if a flake). Incomplete measurements (if the artifact is broken and incomplete) are identified by a symbol in front of the number. Raw material is identified by macroscopic examination, 10x hand-lens and comparison with known geological sample.

The analytical details are coded in the following manner:

Taphonomic attributes indicative of the state of preservation, formational history and evidence of fluvial disturbance.

- State of preservation and dulling of the working edges, observations of two separate edges: fresh, slightly abraded, abraded and very abraded [1 and references therein].
- Difference between states of abrasion of edges is noted in a separate column.
- Syn-or postdepositional chemical alteration: patinated, double patina, weathered.
- Burning: yes, no, partial, calcined.
- Breakage: complete, broken, almost complete

Attributes related to the mode of production
Blank:

- flakes,
- flake fragment,
- pebble,
- core,
- core fragment,
- chunk,
- shaped blank,
- natural clast,
- recycled tool fragment.

Cortex:

- presence or absence,
- type of cortex (pebble cortex, fresh, abraded, natural surface)
- percentage of cortex or natural surface (intervals of 0-4).

Type of flake:

- flat flake (flakes with non conchoidal fracture, see text "Split pebbles and the bipolar technique"),
- bipolar flake,
- biface shaping flake and possible shaping flake [2, 3],

- tool retouch flake,
- flakes from pièces esquillées (flakes from scaled pieces [4]).
- flakes with unidirectional or bidirectional parallel scars on the dorsal face,
- flakes with unidirectional convergent scars,
- flakes with parallel and orthogonal scars,
- (the last three types of flakes are further subdivided if they have a cortex back, or cortex on the lateral or distal edge)
- Kombewa flake,
- Pseudo-Levallois,
- Débordant,
- With centripetal dorsal scars (but not Levallois).
- Ordinary flakes (the generic product of any kind of core) can be cortical, partly cortical or non- cortical.

Platform type:
- cortical,
- plain,
- dihedral,
- facetted,
- ridge (i.e. dihedral or facetted but no individual preparation),
- punctiform,
- shattered (on ventral face),
- broken at knapping,
- removed.

Knapping accident:
- Plunging termination
- Hinged termination
- Siret

Blades. They are quite rare in these assemblages**.** Their classification is in [5 and 6, Supplementary Online Materia].

Features specific to a particular artifacts are recorded under Observations, Photo, and Drawings.

Small tool classification

We follow a very simplified version of Bordes' typology [4]. Pieces with an irregular and discontinuous or very marginal retouch (marginal but continuous and regular retouch on a fresh piece, hence very probably not due to postdepositional damage) are classified as 'retouched piece". Retouched pieces on small pebbles, cores or negative blanks are classed following the system for small tools on flakes, when the retouched edge is clearly a scraper, a notch or a denticulate edge.

Cores

Levallois cores

- Recurrent centripetal
- Recurrent with parallel, unidirectional or bidirectional removals
- With a preferential (single invasive) removal
- Undetermined Levallois core

Non-Levallois cores

- With unidirectional parallel removals (debitage surface subparallel to the great plane of the volume)
- With unidirectional convergent removals (debitage surface subparallel to the great plane of the volume)
- With unidirectional removals, secant to the great plane of the volume
- With centripetal removals (non Levallois)
- Cores with non-conchoidal removals
- Bipolar core
- Multidirectional core (with removals on more than two debitage surfaces)
- Core with a single removal
- Undetermined core (irregular or too few negatives)
- Tested raw material

Bifaces

The bifaces were not classed using Bordes' typology or any other typology. They were studied in two ways:

1. With a simple Excel database containing the catalog number, the raw material, the blank, taphonomic observations, and the maximum length, breadth and thickness.

2. By detailed outline drawings of each biface, complete or broken, illustrating the order of removals to reconstruct the various stages of manufacture (shaping, finishing and a few cases of reworking). The symbolic conventions used to indicate the scar direction lines (arrows) and the presence or absence of negative bulbs (the presence is indicated by a dot at the end of the arrow) are those recommended by [7]. The graphic representation is enhanced by color:

a) the negatives of shaping flakes, which the knapper used to build the volume of the bifacial piece, are filled in grey;

b) the negatives of retouch flakes to sharpen and finish the biface cutting edge are filled in yellow, orange or red, from the oldest to the youngest.

When possible we order the negatives from the oldest to the most recent to follow the chronology of removals on each face and from one face to the other. This allows us to understand how shaping is related to the biface volume. This graphic representation illustrates the extent and the chronology of retouching and was designed by Sylvain Soriano. Almost all bifaces were photographed with a Nikon D800 connected to a laptop.

A similar though simpler procedure was used for the analysis of each Levallois and non-Levallois core. For bifaces and cores the chronology of flaking was established through observations of the overlap of negatives with a magnifying glass under oblique light.

References

1. Harding P, Gibbard PL, Lewin J, Macklin MG, Moss EH. The transport and abrasion of flint handaxes in a gravel-bed river. In: Sieveking G de G, Newcomer M, editors. The Human Uses of Flint and Chert. Cambridge: Cambridge University Press; 1987. pp. 115–126.

2. Bradley B, Sampson CG. Analysis by replication of two Acheulian artifact assemblages. In: Bailey G, Callow P, editors. Stone Age Prehistory: Studies in Memory of Charles McBurney. Cambridge: Cambridge University Press; 1986. pp. 29–45.

3. Newcomer MH. Some Quantitative Experiments in Handaxe Manufacture. World Archaeol. 1971; 3: 85–104.

4. Villa P, Delagnes A, Wadley L. A late Middle Stone Age artifact assemblage from Sibudu (KwaZulu-Natal) : comparisons with the European Middle Paleolithic. J Archaeol Sci. 2005; 32: 399–422.

5. Villa P, Soriano S, Teyssandier N, Wurz S. The Howiesons Poort and MSA III at Klasies River main site, cave 1A. J Archaeol Sci. 2010; 37: 630–655.

6. Soriano S, Villa P, Wadley L. Blade technology and tool forms in the Middle Stone Age of South Africa: the Howiesons Poort and post-Howiesons Poort at Rose Cottage Cave. J Archaeol Sci. 2007; 34: 681–703.

7. Inizan M-L, Reduron-Ballinger M, Roche H, Tixier J. Technology and Terminology of Knapped Stone. Nanterre: Cercle de Recherches et d'Etudes Préhistoriques; 1999.
